# Efficacy of online mindfulness for the treatment of insomnia in pregnancy: A randomized clinical trial

**DOI:** 10.1371/journal.pone.0322931

**Published:** 2025-05-09

**Authors:** Dahee Wi, Rachel Y. Lee, Ira Kantrowitz-Gordon

**Affiliations:** 1 Department of Human Development Nursing Science, College of Nursing, University of Illinois Chicago, Chicago, Illinois, United States of America; 2 Department of Biomedical Informatics, Columbia University Irving Medical Center, New York, New York, United States of America; 3 Child, Family, and Population Health Nursing, University of Washington, Seattle, Washington, United States of America; Galgotias University, INDIA

## Abstract

Sleep deficiency is common during pregnancy, with consequences for maternal and fetal health. This pilot study assessed the efficacy, feasibility, and acceptability of a six-week, online, mindfulness-based intervention (OPTIMISM) in pregnant women with sleep deficiency. Participants were randomized to either mindfulness (self-directed learning modules about mindfulness meditation, sleep challenges in pregnancy, and behavioral strategies) or education-only control conditions. Participants completed surveys and wore an actigraph with daily sleep diaries for 8 days at baseline and post-intervention. The primary outcome was sleep quality. Secondary outcomes included actigraphy, sleep-related impairment, sleep disturbance, fatigue, depression, anxiety, postnatal depressive symptoms, well-being, and quality of life. Exploratory outcomes included feasibility, acceptability, self-management, and behavior change. Efficacy was estimated with analysis of covariance, comparing mean post-test scores corrected for baseline. Of the 351 women screened, 163 were eligible, 64 enrolled, and 59 were randomized. 45 participants (OPTIMISM, 23; control, 22) completed post-intervention assessments and were included in the analysis. The mean satisfaction with the OPTIMISM was higher than the control group (OPTIMISM, 4.1; control, 3.8). The mean sleep quality score was significantly improved in the OPTIMISM group compared to that in the control group after adjusting for baseline score (OPTIMISM, 5.4; control, 7.6; *p* =.008; partial h^2^ =.157). There were similar improvements in sleep-related impairment, sleep disturbance, fatigue, and depressive symptoms, but no differences in other outcomes. Findings suggest that OPTIMISM improves subjective sleep quality and psychological distress, including fatigue, depression, and anxiety during pregnancy and should be tested in larger trials with longitudinal follow-up.

**Trial registration:** ClinicalTrials.gov, NCT04016428. Registered on 11 July 2019.

## Introduction

Insomnia is defined as difficulty initiating, maintaining sleep, and/or suffering from early awakenings for at least three nights per week over at least three months with associated clinically significant functional impairment [1]. Insomnia can begin as early as the first trimester and becomes more prevalent during the third trimester [2], affecting an estimated 40% of women in the third trimester of pregnancy [3]. Although pregnancy accounts for a relatively short part of an individual’s life, insomnia during pregnancy is associated with significant adverse health-related outcomes that may impact short and long-term health [4], increased systemic inflammation [5], and gestational diabetes mellitus [6]. Insomnia is also associated with psychological symptoms such as depression [7] and anxiety [8], resulting in reduced quality of life in this population [9].

Cognitive behavior therapy for insomnia (CBT-I) is recommended as the first-line treatment in the management of insomnia [10]. However, a meta-analysis suggested that only an estimated 36% of patients were in remission from insomnia at the post-assessment of CBT-I [11], and standard CBT-I has minimal effects on rumination [12]. As an alternative, emerging evidence has suggested that mindfulness interventions and an integrated intervention (combining mindfulness meditation with CBT-I) are useful in attenuating the cognitive and somatic arousals, which is a key complaint of insomnia [13,14]. Mindfulness-based interventions (MBIs) offer the practice of meditation to cultivate non-judgmental, present-moment awareness, self-compassion, and non-attachment to outcomes [15]. Previous research has suggested that MBIs were associated with decreased psychological distress (e.g., depression, anxiety, and stress) [16,17]; improved fatigue [18] and decreased subjective sleep deficiency in the general population [19]. Recent studies demonstrated that MBIs can potentially provide positive outcomes (e.g., improving insomnia, decreasing psychological distress, building stress tolerance, etc.), specifically for pregnant women [20–23].

Frequently reported barriers to the implementation of MBIs among pregnant women included lack of time, commuting distance to classes, and scheduling conflicts [24,25]. Digital MBIs (web- and mobile-based platforms) may overcome these barriers by saving commuting distance and time. Moreover, digital platforms are advantageous in terms of cost-effectiveness in addressing perinatal health and the potential to reach larger participants compared to traditional interventions [23,26], especially during the COVID-19 pandemic. Recent evidence suggests that digital MBIs are effective in reducing depression and anxiety symptoms in pregnant women [27]. However, despite the increasing use of digital MBIs for perinatal mental health, research specifically targeting insomnia during pregnancy remains limited [22]. It is still understudied whether digital MBI has effects on both subjectively- and objectively measured sleep, as well as psychological distress and quality of life for women during pregnancy. Therefore, the current study aimed to assess the efficacy, feasibility, and acceptability of a six-week online intervention combining mindfulness meditation and CBT-I for pregnant women with insomnia.

## Materials and methods

### Study design and participants

This study was a randomized controlled pretest-posttest study designed to determine the feasibility, acceptability, and preliminary efficacy of the online, mindfulness-based intervention (OPTIMISM) [28]. Assessments were conducted at baseline and post-intervention. All participants were provided with an electronic or paper copy of consent prior to participation in the study. This study received approval from the University of Washington Institutional Review Board (STUDY00005267). All participants provided written informed consent prior to enrollment in the study.

Study data were collected and managed using Research Electronic Data Capture (REDCap) hosted at a collaborating institution. REDCap is a secure, web-based software platform designed to support data capture for research studies [29]. Inclusion criteria included the following: 1) viable pregnancy in the second trimester (12–28 weeks gestation); 2) prior diagnosis of depression and currently in remission (PHQ-9 < 12); 3) self-report of insomnia (Score > 7 on the Insomnia Severity Index); 4) age 18 or older; 5) have access to an internet-enabled smartphone, tablet, or computer; 6) English fluency. Participants were allowed to participate regardless of whether they were receiving psychotropic medications or psychotherapy. Participants were excluded if they 1) had known severe congenital fetal anomalies, fetal demise, or expected neonatal death; 2) had a diagnosis of Major Depressive Disorder in the past 2 months; 3) had other significant psychiatric illnesses requiring treatment; 4) were currently hospitalized; 5) had a prior diagnosis of obstructive sleep apnea or restless leg syndrome; 6) had a positive self-report screen for restless leg syndrome; 7) were at high risk for obstructive sleep apnea based on a four-variable model (snoring frequency, chronic hypertension, age, and body mass index); 8) practiced mindfulness or meditation regularly (at least weekly); or 9) regularly worked night-shift.

### Modification to the protocol

During the study, a couple of modifications to the original protocol were made due to difficulties in participant recruitment. First, the inclusion criteria were broadened to include eligible participants without a history of depression because these criteria often excluded interested, otherwise qualified participants. Second, due to the COVID-19 outbreak during this study, all interactions with participants after March 2020 were conducted remotely via Zoom or phone calls. Since all interactions with participants, including the enrollment visits, were changed to online methods, the inclusion criteria for participants residing in the greater metropolitan area were broadened to include those in other geographical areas to increase recruitment success.

### Procedures

#### Recruitment, selection, and enrollment.

Participants were recruited between April 2019 and December 2020 via flyers, social media, email listservs, and snowballing methods. Recruitment flyers were distributed to community-based prenatal care clinics, high-risk pregnancy clinics, maternity service providers, and perinatal mental health providers in the metropolitan area. Recruitment posts were posted to social media platforms such as Facebook, Nextdoor, and Craigslist. Participants were also recruited through research recruitment sites such as Participate in Research. The electronic medical record system at a local university medical center was used to identify eligible participants, who were then contacted via email with study information and a link to the eligibility survey. Interested participants were screened by telephone or completing an eligibility survey on an online platform, REDCap, to determine whether they meet inclusion and exclusion criteria. Potential participants were notified of their eligibility immediately when screening was completed.

An enrollment visit was then scheduled with eligible and interested participants. At the enrollment visit, participants were provided with a detailed description and procedure of the study and were given consent forms to sign. Participants were also given verbal and written instructions on completing the baseline assessments, wearing the actigraph, and completing daily sleep diaries.

#### Baseline (T1) assessment.

After the enrollment visit and receiving consent, participants completed baseline assessments. Baseline assessments included self-reported questionnaires (demographics, pregnancy, and general health status, self-reported sleep, mood, and self-management variables) and objective sleep monitoring by wearing an actigraph and completing daily sleep diaries for 8 consecutive days (7 nights). Online questionnaires and daily sleep diaries were sent to participants using REDCap.

#### Randomization and intervention.

After completing the baseline assessment, participants were randomly assigned using block randomization with a 1:1 allocation ratio. A computer-generated randomization sequence with a block size of 4 was used to ensure balance across study arms. The randomization sequence was generated by a research assistant; the assignment was implemented using REDCap’s automated allocation system to prevent selection bias. Participants were told the study was testing two alternative online interventions to improve sleep during pregnancy and were not informed of the nature of the two interventions. Participants were blinded to their intervention allocation, but research staff responsible for assessment and data collection were aware of group assignments.Participants randomized to the intervention group received the OPTIMISM, consisting of six weekly online self-directed learning modules about mindfulness meditation, sleep challenges in pregnancy, and behavioral strategies to improve sleep. OPTIMISM includes components of two in-person MBIs: mindfulness-based childbirth and parenting and mindfulness-based therapy for insomnia. The intervention activities focused on using mindful awareness as a sleep self-management technique to increase total sleep time (TST) and sleep efficiency (SE). The intervention also integrates concepts of sleep restriction, sleep hygiene, and stimulus control. Intervention modules were developed using Articulate Storyline, Version 3.3, and published online through Articulate Online (Articulate Global, Inc.). Each weekly module was approximately 20 minutes long and included didactic content on sleep, pregnancy, and mindfulness meditations. Didactic contents were delivered using interactive text, video, and audio meditations. OPTIMISM program modules content by session are displayed in Table 1. Participants were able to choose a time to complete the weekly modules. During the six weeks of the intervention, participants completed daily sleep diaries either online or on paper. Daily sleep diaries asked about pregnancy symptoms interfering with sleep with reports of daily meditation practice. Participants also received an individualized recommended sleep schedule at the end of the second week of the program. From the third week, participants received weekly sleep reports that included a visualization of their diary-reported bedtime and wake time, along with their recommended sleep schedule. The sleep report also included any recommended changes to their sleep schedule for the coming week. Participants received reminders to complete daily sleep diaries and weekly modules sent by text or email at intervals agreeable to participants. Participants were also given access to an online anonymous discussion board to interact with research staff and other participants. Support from research staff was available during daytime hours by text message, email, or phone communication.

**Table 1:** Session content for OPTIMISM Group.

Module	Content	Mindfulness practices
Introduction	Facts about sleep Sleep and health Sleep in pregnancy Pregnancy sleep strategies Introduction to mindfulness	Mindfully eating a raisin Awareness of breathing
Timing	Diet and sleep Insomnia Sleep schedule and window Bedtime routines Mindfulness and sleep Mindfulness and labor	Body scan
Activity	Reviewing and adjusting sleep schedule Stimulus control Mindful movement Physical activity in pregnancy Movement and rest in labor	Mindful walking
Acceptance	Reviewing and adjusting sleep schedule Choices and control in labor Acceptance and letting go	Being with baby
Self-compassion	Reviewing and adjusting sleep schedule Mood and sleep Self-care and self-compassion	Loving kindness
Postpartum and beyond	Reviewing and adjusting sleep schedule Physical recovery postpartum Newborn sleep Postpartum sleep Postpartum mindfulness	Being with baby

**Note.** OPTIMISM = online prenatal trial in mindfulness sleep management

Participants who were randomized to the control group received education-only control (EOC) intervention which included weekly educational online modules and completion of daily sleep diaries (Table 2). Online modules were developed on Articulate Storyline and published on Articulate Online. Each module was approximately 20 minutes and could be completed at a time chosen by the participant. EOC modules did not include any content on sleep hygiene, sleep scheduling/bed restriction, stimulus control, or mindfulness and meditation. The control group also completed daily sleep diaries for the duration of the intervention. EOC participants were also given access to an online discussion board limited to other EOC participants and research staff. Instead of treatment as usual, an active control was chosen to control for non-specific attention and self-monitoring effects [30]. Having an EOC arm allows for any outcome differences between the two groups to be derived from the active treatment elements of the OPTIMISM intervention.

**Table 2:** Session content for education only control group.

Module	Content
Introduction to sleep	Facts about sleep Sleep and health Sleep in pregnancy
Sleep strategies	Diet and sleep Sleep and environment Bedtime routines Pregnancy sleep strategies
Physical activity	Benefits of physical activity in pregnancy How to exercise in pregnancy Positions, activity, and rest in labor
Mood and sleep	Poor sleep, emotions, and mood Depression in pregnancy Self-care
Medications and sleep	Over the counter medications Prescription medications Substances (caffeine, alcohol, nicotine) Labor pain medications
Sleep postpartum	Physical recovery postpartum Newborn sleep Postpartum sleep Physical activity postpartum

#### Post-Intervention (T2) assessments.

After completing the 6-week interventions, participants were asked to complete post-intervention assessments which included completing self-report questionnaires, completing daily sleep diaries, and wearing an actigraphy for 8 consecutive days (7 nights). Participants received a $50 retail gift card upon completion of the T2 assessment.

### Measures

Participants completed a survey including demographic information. Demographic characteristics, including age, gender, race, ethnicity, level of education, employment status, marital/partner status, and geographical location, were collected.

#### Primary outcome.

As a primary outcome, participants completed the Pittsburgh Sleep Quality Index (PSQI), a 19-item survey to assess sleep quality and usual sleep habits over the past month. PSQI consists of subscales for sleep quality, latency, duration, sleep efficiency, sleep disturbances, use of sleeping medications, and daytime dysfunction. Survey items are rated on a 0–3 Likert-type scale, with higher scores indicating poor sleep quality and poor sleep habits. Total scores range from 0 to 21. A global score > 5 indicates clinically significant sleep disturbances. A change of 3.0 suggests a clinically significant difference in the score [31].

#### Secondary outcomes.

Secondary clinical outcomes included objective and self-reported measures of sleep, mood, well-being, and quality of life. Measures were chosen to meet the funder’s requirements to adopt the common data elements in the Common Data Repository for Nursing Science [32]. Objective sleep was measured with wrist actigraphy (Actiwatch Spectrum Plus, Philips Respironics, Murrysville, PA). Self-reported sleep was assessed using online or paper sleep diaries. Participants were asked to wear the actigraph and keep sleep diaries for 8 days at baseline and post-intervention. For all self-report measures except the Edinburgh Postnatal Depression Scale (EPDS), raw scores were converted to standardized T-scores (mean = 50, SD = 10) according to PROMIS scoring guidelines.

Actigraphy-measured sleep variables included total sleep time (TST), defined as the number of minutes of all time spent asleep; sleep efficiency (SE), a measure of sleep quality calculated as the ratio of TST divided by total time in bed × 100, with values closer to 100 meaning more efficient (better) sleep; sleep onset latency (SOL), defined as the number of minutes from getting into bed to falling asleep; total wake time (TWT), defined as the total number of minutes spent awake during the rest period; and wake after sleep onset (WASO), defined as the number of minutes awake between time falling asleep and wake time.

Sleep-related impairment was measured using the Patient-Reported Outcomes Measurement Information System (PROMIS) Sleep-Related Impairment (v.8a), which is an eight-item questionnaire that uses a 5-point Likert-type scale ranging from 1 (not at all) to 5 (very much). This questionnaire measures self-reported perceptions of alertness, sleepiness, and tiredness during usual waking hours and perceived functional impairments during wakefulness associated with sleep problems or impaired alertness in the past 7 days [33]. Total scores range from 8 to 40, with higher scores meaning increased impairment.

PROMIS Sleep Disturbance Short Form (v.6a) is a six-item self-report questionnaire assessing the perception of sleep quality and difficulty falling asleep over the past 7 days [33]. Items are measured on a 5-point Likert-type scale with total scores ranging from 6 to 30. Higher scores indicate increased sleep disturbance.

Fatigue was measured using the PROMIS Fatigue-Short Form (v.6a). This six-item self-report questionnaire measures self-reported feelings of tiredness and the impact on the ability to function normally over the past 7 days [34]. Items are scored on 5-point Likert-like scales with total scores ranging from 6 to 30. Higher scores indicate increased fatigue.

PROMIS Depression Short Form (v.6a) was used to measure self-reported depression symptoms in the past week [35]. This questionnaire uses a 5-point Likert-like scale with total scores ranging from 6 to 30. Higher scores indicate increased depression symptoms.

Anxiety was measured using the PROMIS Emotional Distress- Anxiety Short Form (v.6a), a six-item self-report questionnaire assessing anxiety symptoms in the past week [35]. The questionnaire uses 5-point Likert-like scales with total scores ranging from 6 to 30. Higher scores indicate increased anxiety symptoms.

Postnatal depression symptoms were measured using the Edinburgh Postnatal Depression Scale (EPDS) [36]. EPDS is a ten-item self-report questionnaire validated for use in pregnant and postpartum populations [37]. The questionnaire asks questions about depressive symptoms in the past week using 3-point Likert-like scales with the sum score ranging from 0 to 30. Higher scores indicate increased depression symptoms, and a score of 10 or greater indicates an elevated risk of depression.

Positive affect and well-being were measured using the Neuro-QOL Positive Affect and Well-Being Short Form, a nine-item self-report questionnaire that measures the frequency of positive emotions [38]. Items are measured on a 5-point Likert-like scale with total scores ranging from 9 to 45. Higher scores indicate increased positive emotions.

Short Form (36) Health Survey (SF-36) is a 36-item self-reported questionnaire asking about current health status and quality of life. Subscales include vitality, physical functioning, bodily pain, general health perceptions, physical role functioning, emotional role functioning, social role functioning, and mental health [39]. Total scores range from 0 to 100, with higher scores indicating better health and quality of life.

#### Exploratory outcomes.

Exploratory outcomes included measures of self-efficacy, self-regulation, acceptance, and state mindfulness. Self-efficacy in emotion management was measured using the PROMIS Self-Efficacy Emotion Management Short Form (v.4a), a four-item self-report questionnaire that assesses confidence in handling emotions [40]. Items are scored on a 5-point Likert-like scale with total scores ranging from 4 to 20. Raw scores were converted to t-scores based on PROMIS guidelines, with higher scores indicate a greater ability to handle emotions.

Sleep problem acceptance was measured using the Sleep Problem Acceptance Questionnaire (SPAQ), an eight-item self-report questionnaire measuring insomnia acceptance [41]. Items are measured on 6-point Likert-type scales with total scores ranging from 0 to 48. Subscales include activities, engagement, and willingness. Higher scores indicate a higher level of acceptance of insomnia.

Self-regulation was measured using the Index of Self-Regulation (ISR). ISR is a nine-item self-report questionnaire that measures the level of motivation and self-regulation for health-related behavior change [42]. Items are measured on 6-point Likert-type scales with total scores ranging from 6 to 54, with higher scores indicating greater self-regulation.

Mindfulness was measured using the Five Facet Mindfulness Questionnaire Short Form (FFMQ-SF). FFMQ-SF is a 24-item self-report questionnaire that assesses the trait-like tendency to be mindful in daily life [43]. The questionnaire consists of five subscales that measure different aspects of mindfulness. The subscales include observing, describing, acting with awareness, non-judging, and nonreactivity. Items are measured on 5-point Likert-type scales, with four subscales scores ranging from 5 to 25 and one of the subscale ranges from 4 to 20. Higher scores indicate higher levels of mindfulness. The FFMQ-SF has been validated for use in pregnant populations with Cronbach’s alpha = .89 [44].

### Feasibility and acceptability

Feasibility measures included examination of recruitment, retention, intervention adherence, and acceptability. The feasibility of recruitment was measured by calculating the proportion of enrolled participants from those who have completed the eligibility survey. Retention was determined by the proportion of participants who have completed the study through the post-assessment surveys from those who have enrolled in the study. Intervention adherence was assessed by the number of online modules completed, with a range of 0–6. Intervention acceptability was measured using an investigator-developed self-report 8-item questionnaire adapted from a similar feasibility trial [45]. Questions assess satisfaction, usefulness for managing sleep and stress, and ease of use using a 5-point Likert-type scale running from 1 (strongly disagree) to 5 (strongly agree). The total scores range from 8 to 40, with higher scores meaning increased acceptability. Open-ended questions were also used to seek feedback about the helpful and unhelpful aspects of the intervention. The questions asked about the content and the process (Internet delivery, mobile device compatibility, and time involved) of the intervention: 1) Would you like to change anything about the modules? If yes, what would you like to change?, 2) What aspects of the program did you find helpful?, 3) What aspects of the program did you find unhelpful or difficult? Answers to the open-ended questions were analyzed using the inductive content analysis method. The analysis process involved open coding, grouping, categorization, and abstraction by two authors [46].

### Statistical analyses

Descriptive statistics were used to summarize participants’ demographic characteristics collected at baseline. Analysis of covariance (ANCOVA) was conducted to assess the differences in mean outcomes between intervention groups (intervention vs. control) at baseline and post-assessment, adjusting for baseline scores to test the efficacy of the intervention. Residual normality was examined using histograms and Q-Q plots, confirming that residuals were approximately normally distributed. Homogeneity of regression slopes was assessed by testing interaction terms between baseline scores and intervention groups, with no significant interactions detected, confirming that the assumption was met. Levene’s test indicated no violations of homogeneity of variance. Adjusted post-intervention means and 95% CIs were computed for each outcome to quantify group differences while controlling baseline scores. Partial η² was reported to estimate effect size, providing insight into the magnitude of group differences and informing future clinical trials. Additionally, McNemar’s test was used to evaluate changes in the proportion of participants with an EPDS score ≥10 between pre- and post-assessment. Statistical significance was set at p < .05.

## Results

### Feasibility

**Fig 1:**
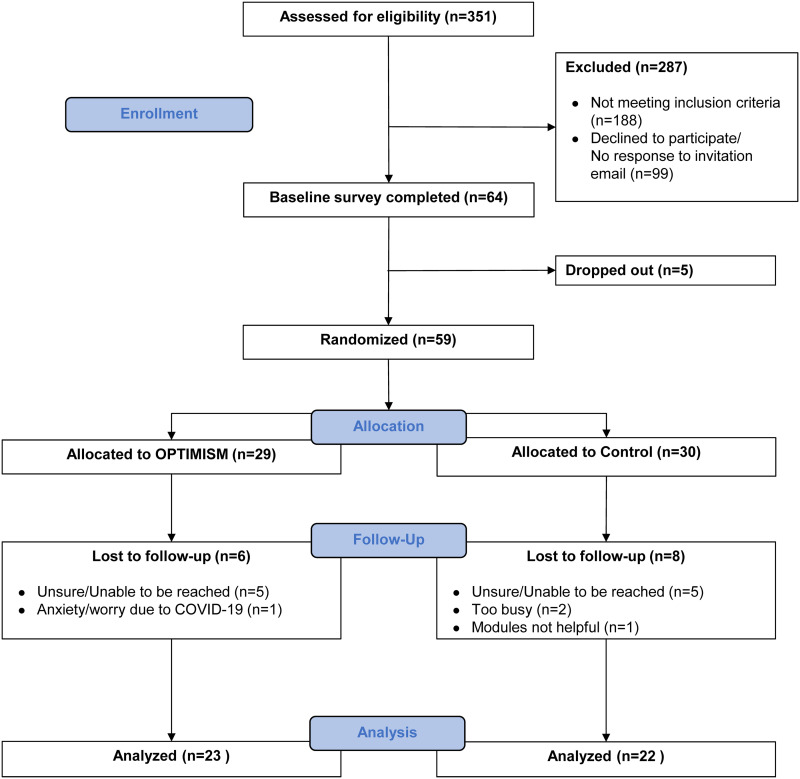
CONSORT flow chart.

Fig 1 displays the CONSORT diagram describing the flow of participants from enrollment through data analysis. Of the 351 participants who completed the initial eligibility survey, 188 did not meet the study inclusion criteria and 99 declined to participate or did not respond to the study invitation email. A total of 64 (18.2% of those who were screened for eligibility) were enrolled and completed the baseline questionnaires. Out of the 64 who were enrolled, 5 dropped out before randomization. Therefore, 59 were randomized to either the intervention or control group. Of the 29 who were randomized to the intervention group, 23 (79.3%) received the intervention, and 6 were lost to follow-up. Of the 30 who were allocated into the control group, 7 were lost to follow up, and a total of 22 participants (73.3%) were included in the analysis. From both groups, a total of 45 were included in the analysis. The average number of modules completed in both groups was 4.8 ± 2.3. Of the OPTIMISM group, the average number of modules completed before dropping was 1.0 ± 1.3; 2.1 ± 1.4 in the control group. The average number of days women in the OPTIMISM group practiced meditation was 4.7 ± 1.9 days per week.

### Acceptability

Of the participants, 73.3% used more than one device (e.g., smartphone, tablet PC, laptop, or desktop) to access the intervention. The average minutes to complete weekly modules were 25.8 ± 12.3 minutes in the OPTIMISM group and 16.8 ± 6.6 minutes in the control group. Table 3 describes the satisfaction with OPTIMISM. The total satisfaction score was higher in the OPTIMISM group versus the control group (OPTIMISM: 32.8 ± 5.1, 30.1 ± 6.9). Generally, those who were in OPTIMISM group reported higher satisfaction on most items except online delivery ease of use and internet device compatibility. Regarding the sleep diary, 91.1% used the online sleep diary exclusively, while 6.7% used both the online and paper versions. The average time to complete a sleep diary was 4.8 ± 2.3 minutes. The overall sleep diary satisfaction score was lower in the OPTIMISM group versus the control group (2.6 ± 0.8, 2.8 ± 0.9, respectively), while the score of incorporating sleep diary into one’s daily routine was higher in the OPTIMISM group than the control group (3.2 ± 0.1, 2.8 ± 0.9, respectively).

**Table 3:** Satisfaction with OPTIMISM.

	Total (n = 45)	OPTIMISM (n = 23)	Control (n = 22)
	**Mean (SD)**		
Overall, I am satisfied with the OPTIMISM program	3.96 (0.93)	4.13 (0.82)	3.77 (0.02)
I found the OPTIMISM program enjoyable	3.78 (0.97)	3.96 (0.88)	3.59 (1.05)
The educational information about sleep in pregnancy was useful.	4.07 (0.94)	4.35 (0.78)	3.77 (1.02)
I plan to use what I learned to sleep better now and after baby comes	4.16 (0.90)	4.43 (0.79)	3.86 (0.94)
The OPTIMISM program helped me improve my sleep	3.84 (1.02)	4.26 (0.86)	3.41 (1.01)
The program helped me manage stressors associated with pregnancy, delivery or parenting	3.51 (0.97)	3.78 (0.85)	3.23 (1.02)
I found the online delivery of the program easy to use	3.93 (1.05)	3.74 (1.14)	4.14 (0.94)
The online delivery of the program was compatible with my internet device	4.20 (0.97)	4.13 (0.92)	4.27 (1.03)
Total score	31.44 (6.17)	32.78 (5.14)	30.05 (6.94)

**Note.** OPTIMISM = Online Prenatal Trial in Mindfulness Sleep Management, SD = Standard Deviation.

Several themes from the open-ended qualitative questions about acceptability were presented in Table 4. Suggested improvements for intervention included the modification of the module interface (e.g., not user-friendly, narration with robotic voice), and resolving technical issues (e.g., streaming issues, tracking related glitches in the system). Participants in the OPTIMISM group reported mindfulness-related content and information about sleep were helpful. In terms of the unhelpful aspects of the intervention, the OPTIMISM group reported mindfulness practices were too long, while the control group reported survey burden for daily sleep diary.

**Table 4:** Themes from participant feedback on intervention acceptability.

Category	Group	Theme
Need for improvement	OPTIMISM	Module interface
		Robotic voice
	Control	Modules unhelpful
		Robotic voice
Helpful aspects	OPTIMISM	Mindfulness related contents
		Information/education about sleep
	Control	Sleep diary/tracking
		Information/education about sleep
Unhelpful aspects	OPTIMISM	Module interface/technical issues
		Mindfulness practice too long
	Control	Modules unhelpful
		Sleep diary burdensome

**Note.** OPTIMISM = Online Prenatal Trial in Mindfulness Sleep Management

### Baseline demographic characteristics

Baseline demographic characteristics of the participants are presented in Table 5. The average age for total participants was 33.6 ± 4.1 years (OPTIMISM = 34.1 ± 4.2 years, control = 33.01 ± 3.9 years), and average gestational weeks were 18.7 ± 4.6 weeks (OPTIMISM = 18.8 ± 4.4, control = 18.6 ± 4.8). Most participants in the total sample were White (68.9%) or Asian (22.2%); 95.6 of the total sample identified as non-Hispanic or Latino.

**Table 5:** Baseline characteristics of study participants.

Characteristic	Total (n = 45)	OPTIMISM (n = 23)	Control (n = 22)
**Age, mean (SD)**	33.6 (4.05)	34.13 (4.19)	33.05 (3.92)
**Gestational age (weeks), mean (SD)**	18.7 (4.6)	18.8 (4.4)	18.6 (4.8)
**Number of people in household, mean (SD)**	2.47(0.89)	2.48 (0.85)	2.45 (0.96)
**Gender (female), n (%)**	45 (100)	23 (100)	22 (100)
**Parity, n (%)**			
Nullipara	28 (62.2)	14 (60.9)	14 (63.6)
Multipara	17 (37.8)	9 (39.1)	8 (36.4)
**Race, n (%)**			
White	31(68.9)	14 (60.9)	17 (77.3)
Asian	10 (22.2)	7 (30.4)	3 (13.6)
Black/African American	1 (2.2)	0 (0)	1 (4.5)
More than one	3(6.7)	2 (8.7)	1 (4.5)
**Ethnicity, n (%)**			
Not Hispanic or Latino	43 (95.6)	21 (91.3)	22 (100)
Hispanic or Latino	1 (2.2)	1 (4.3)	0 (0)
Unknown	1 (2.2)	1 (4.3)	0 (0)
**Highest level of education, n (%)**			
Associate degree (academic program)	3 (6.7)	2 (8.7)	1 (4.5)
Associate degree: occupational/technical/vocational program	2 (4.4)	1 (4.3)	1(4.5)
Bachelor’s degree (e.g., BA, AB, BS, BBA)	10 (22.2)	5 (21.7)	5 (22.7)
Master’s degree (e.g., MA, MS, MEng, MEd, MBA)	24 (53.3)	12 (52.2)	12 (54.5)
Doctoral degree (e.g., PhD, EdD)	2 (4.4)	1 (4.3)	1 (4.5)
Professional school degree (e.g., MD, DDS, DVM, JD)	4 (8.9)	2 (8.7)	2 (9.1)
**Employment status, n (%)**			
Working now	33 (73.3)	17 (73.9)	16 (72.7)
Keeping house	5 (11.1)	3 (13)	2 (9.1)
Student	3 (6.7)	0 (0)	3 (13.6)
Other	4 (8.9)	3 (13)	1(4.5)
**Marital/partner status, n (%)**			
Married	37 (82.2)	21 (91.3)	16 (72.7)
Domestic Partnership	5 (11.1)	1 (4.3)	4 (18.2)
Never married	3(6.7)	1 (4.3)	2 (9.1)
**Geographical location, n (%)**			
Greater metropolitan area	27 (60.0)	14 (60.9)	13 (59.1)
Same state as study location	3 (6.7)	1 (4.3)	2 (9.1)
Other states	13 (28.9)	6 (26.1)	7 (31.8)
Not answered	2 (4.4)	2 (8.7)	0 (0)
**Medication use, n (%)**			
Sleep aid or antihistamines	10 (22.2)	3 (13)	7 (31.8)
Antibiotics	5 (11.1)	4 (17.4)	1(4.5)
Gastrointestinal medications	4(8.9)	1 (4.3)	3 (13.6)
NSAIDs	4 (8.9)	2 (8.7)	2 (9.1)
Others (e.g., immunosuppressant, hormone, stool softener, beta-blocker, etc.)	12 (26.7)	5 (21.7)	8 (36.4)
**Pre-pregnancy BMI, mean (SD)**	23.8 (5.4)	24.8 (6.4)	22.7 (3.9)
**Pre-pregnancy BMI ≥ 30, n (%)**	6 (13.3)	5 (21.7)	1 (4.5)
**Current BMI, mean (SD)**	25.1 (5.5)	26.3 (6.5)	23.9 (4.1)
**Current BMI ≥ 30, n (%)**	10 (22.2)	7 (30.4)	3 (13.6)

**Note.** BMI = Body Mass Index; NSAIDs = Nonsteroidal Anti-Inflammatory Drugs; OPTIMISM = Online Prenatal Trial in Mindfulness Sleep Management; SD = Standard Deviation.

### Primary and secondary outcomes

Table 6 presents the clinical outcomes for the control and OPTIMISM groups at baseline and post-intervention. Participants in the OPTIMISM intervention group experienced significant improvements in sleep quality (partial η² = 0.157).

**Table 6:** Primary and secondary outcomes at baseline and post-intervention.

Variable	Group	Baseline Mean (SD)	Post-intervention Mean (SD)	Adjusted post-intervention mean [95% CI]	*p*	Partial η^2^
PSQI	Control	9.3 (3.9)	7.7 (3.3)	7.6 [6.5, 8.7]	.008	0.157
	OPTIMISM	8.8 (3.5)	5.4 (2.5)	5.4 [4.4, 6.5]		
PROMIS Sleep disturbance	Control	58.3 (6.1)	53.2 (6.3)	52.8 [50.6, 54.9]	.008	0.155
	OPTIMISM	56.0 (6.9)	48.2 (4.3)	48.6 [46.5, 50.7]		
PROMIS Sleep-related impairment	Control	59.0 (7.3)	55.2(7.6)	54.8 [52.5, 57.0]	.002	0.202
	OPTIMISM	57.8 (7.5)	49.3 (6.6)	49.7 [47.5, 51.9]		
PROMIS Fatigue	Control	59.5 (6.7)	54.7 (6.6)	54.3 [51.3, 57.5]	.003	0.188
	OPTIMISM	57.3 (5.9)	47.3 (7.9)	47.6 [44.6, 50.6]		
Positive affect and Well-being	Control	52.8 (8.1)	53.7 (6.5)	54.1 [52.1, 56.1]	.866	0.001
	OPTIMISM	54.4 (4.9)	54.8 (4.9)	54.4 [52.4, 56.3]		
PROMIS Anxiety	Control	52.3 (9.6)	50.3 (8.2)	50.1 [47.5, 52.6]	.576	0.008
	OPTIMISM	52.3 (8.2)	49.1 (7.3)	49.0 [46.5, 51.6]		
PROMIS Depression	Control	49.1(9.2)	49.2 (6.6)	49.8 [47.9, 51.7]	.044	0.093
	OPTIMISM	51.1(8.3)	47.6 (6.6)	47.0 [45.1, 48.9]		
EPDS	Control	7.3 (5.0)	6.0 (4.3)	6.0 [4.8, 7.3]	.517	0.010
	OPTIMISM	7.5 (3.8)	5.5 (3.2)	5.5 [4.2, 6.7]		
SF-36 Total	Control	65.3 (20.3)	67.8(18.4)	69.3 [63.5-75.0]	.85	0.001
	OPTIMISM	70.2 (14.5)	71.5(15.3)	70.1 [64.4-75.7]		

**Note.** CI = Confidence Interval; EPDS = Edinburgh Postnatal Depression Scale; η² = Partial Eta Squared; OPTIMISM = Online Prenatal Trial in Mindfulness Sleep Management; PROMIS = Patient-Reported Outcomes Measurement Information System; PSQI = Pittsburgh Sleep Quality Index; SD = Standard Deviation; SF-36 = Short Form Health Survey 36.

### Objective and self-reported sleep outcomes

Analysis of Covariance revealed that the OPTIMISM group was not related to significant improvements in any of the objective sleep outcomes (SE, TWT, TST) measured by actigraphy (Table 7). Both the OPTIMISM group and control group had similar adjusted post-treatment mean of TST, SE, and TWT measured by actigraphy, with small effect sizes (partial η^2^, ranging from.002 to.025). Similar results were observed in the self-reported sleep diary outcomes (TST, SE, TWT), with no significant improvements by treatment (partial η^2^, ranging from.006 to.027). However, we found significant differences in sleep disturbance (partial η^2 ^= 0.155) and sleep related impairments (partial η^2 ^= 0.202), with large effect sizes.

**Table 7:** Actigraphy outcomes.

Variable	Group	Baseline Mean (SD)	Post-intervention Mean (SD)	Adjusted post-intervention mean [95% CI]	*p*	Partial η^2^
TST (min)	Control	460.2 (45.3)	454.5 (42.9)	444.7 [426.2, 463.3]	.332	0.025
	OPTIMISM	455.8 (55.3)	425.8 (68.5)	432.0 [413.9, 450.2]		
SE (%)	Control	82.3 (6.0)	82.2 (6.3)	81.3 [78.7, 83.8]	.517	0.011
	OPTIMISM	80.9 (6.1)	79.1 (9.7)	80.1 [77.6, 82.6]		
TWT (min)	Control	77.9 (30.8)	79.5 (36.2)	84.1 [70.2, 98.0]	.794	0.002
	OPTIMISM	88.8 (32.9)	85.8 (43.4)	81.5 [68.0, 95.1]		
SOL (min)	Control	25.2 (15.8)	29.8 (23.0)	32.3 [21.6, 43.0]	.416	0.017
	OPTIMISM	32.6 (19.3)	28.5 (27.4)	26.1 [15.7, 36.6]		
WASO (min)	Control	52.6 (18.4)	49.8 (18.6)	50.9 [44.9, 56.9]	.208	0.041
	OPTIMISM	56.1 (22.6)	57.3 (24.8)	56.2 [50.3, 62.1]		

**Note.** OPTIMISM = Online Perinatal Treatment Intervention for Mindfulness and Sleep Improvement; SD = Standard Deviation; SE = Sleep Efficiency; SOL = Sleep Onset Latency; TST = Total Sleep Time; TWT = Total Wake Time; WASO = Wake After Sleep Onset.

### Fatigue, psychological distress, and general health

In terms of fatigue, the OPTIMISM group showed a significantly lower adjusted post-intervention mean of 47.6 (95% CI [44.6, 50.6]), compared to 54.3 (95% CI [51.3, 57.5]) in the control group (*p* = .003, partial η² = .188). This indicates a substantial improvement in fatigue for participants in the OPTIMISM group compared to the control group. However, there were no significant differences between the two groups in their ratings of positive affect and well-being, anxiety, and depressive symptoms. There were no significant differences between groups in postnatal depression measured by EPDS. However, the percentage of participants at increased depression risk for depression (defined by EPDS score ≥ 10) decreased more in the OPTIMISM group (from 34.8% to 8.7%; *p* = .07) than in the control group (31.8% to 22.7%; *p* = .69). For general health, measured by the SF-36, there were no significant differences between groups.

### Exploratory outcomes

There were no significant differences between the control and OPTIMISM intervention groups in participants’ pre- to post-intervention changes in self-efficacy in emotion management, sleep problem acceptance, self-regulation, or mindfulness (Table 8).

**Table 8:** Exploratory outcomes at baseline and post-intervention.

Variable	Group	Baseline Mean (SD)	Post-intervention Mean (SD)	Adjusted Post-intervention Mean [95% CI]	*P*	Partial η^2^
Self-efficacy emotion management	Control	51.1 (8.4)	52.4 (9.3)	51.5 [48.7, 54.3]	.271	0.029
	OPTIMISM	48.7 (7.7)	48.5 (7.6)	49.3 [46.6, 52.1]		
Sleep problem acceptance questionnaire	Control	30.7 (8.9)	31.4 (8.2)	31.3 [29.0, 33.7]	.55	0.009
	OPTIMISM	30.4 (7.3)	32.2 (5.7)	32.3 [30.0, 34.6]		
Index of self-regulation	Control	34.8 (6.0)	35.9 (7.0)	35.4 [33.3, 37.5]	.279	0.028
	OPTIMISM	33.4 (6.6)	36.5 (5.6)	37.0 [34.9, 39.0]		
Five facet mindfulness questionnaire	Control	82.8 (12.4)	84.6 (13.06)	85.8 [51.4, 120.2]	.94	<0.001
	OPTIMISM	84.7(11.3)	86.6 (11.7)	85.6 [46.4,124.8]		

**Note.** CI = Confidence Interval; η² = Partial Eta Squared; OPTIMISM = Online Prenatal Trial in Mindfulness Sleep Management; SD = Standard Deviation.

No significant harms or unintended effects were observed in either the intervention or control group throughout the study period. Participants in both groups reported their experiences via feedback forms, and any issues were documented and reviewed by the research team. Minor issues, such as occasional technical difficulties in accessing online modules, were promptly addressed, and no adverse events related to intervention or control activities were reported.

## Discussion

The goal of this study was to test the feasibility and efficacy of an online digital treatment for insomnia in pregnancy that combined elements of CBT-I (sleep restriction, stimulus control) with mindfulness meditation. Participation in the intervention was feasible, with 76% of participants who were randomized completing the study. 97.8% of the participants completed all 6 weekly modules. These results were achieved despite the onset of the COVID-19 pandemic midway through the study. Overall satisfaction was greater than 4 on a 1–5 scale for the mindfulness intervention. We found support for the primary study hypothesis that OPTIMISM intervention would be associated with improved global sleep quality by the end of treatment. During the 6-week study period, PSQI scores of participants assigned to OPTIMISM improved significantly more compared to participants who were randomized to the education-only control. We also found a significant difference in depression symptoms as measured by the PROMIS Depression scale. However, we did not find significant differences between groups on several secondary outcomes such as postnatal depression (measured by EPDS), anxiety, positive affect and well-being, and eight subscales of SF-36, and exploratory outcomes including self-efficacy, self-regulation, sleep problem acceptance, and mindfulness.

Notably, there were no significant differences in objective sleep (TST, SE, TWT) measured by actigraphy and self-reported sleep diary outcomes. The lack of objective differences in sleep despite subjective improvement is consistent with documented differences between objective and subjective sleep measurement during pregnancy [47], well established discrepancies between subjective and objective sleep in people with insomnia [48], and studies that report improvement in subjective symptoms without objective improvement [49]. One possible explanation for this discrepancy is that mindfulness-based interventions primarily target cognitive and emotional responses to sleep disturbances, which may lead to improvements in perceived sleep quality before measurable changes in objective sleep occur. Additionally, behavioral changes, such as modifications in sleep patterns and habits, may require a longer duration to manifest in objective measures. Furthermore, actigraphy, while a valuable tool for sleep assessment, has limitations in detecting certain aspects of sleep continuity, which could contribute to the observed discrepancy. Future studies should explore whether extended intervention periods or follow-up assessments capture delayed objective sleep improvements.

### Comparison to other behavioral sleep interventions in pregnancy

Our findings add to a growing body of evidence that suggests the effectiveness of online digital interventions for the treatment of insomnia in pregnancy. In a recent randomized controlled trial, Manber and colleagues [50] found that women assigned to the 5-session individual cognitive-behavioral therapy experienced significantly greater reductions in insomnia severity (measured by Insomnia Severity Index) and experienced faster remission of insomnia disorder compared to those assigned in the control group.

Additionally, Felder and colleagues [51] found that women who were randomly assigned to receive digital cognitive behavioral therapy had statistically significantly greater improvements in insomnia symptom severity, depressive symptom severity, sleep efficiency, and global sleep quality compared to those in the control group who were given treatment as usual. In contrast to Felder et al.[51]’s findings, our study found no significant differences in outcomes except for sleep quality and depressive symptoms. Our study’s use of an active education-only control group may have reduced the difference between experimental and control groups, in comparison to Felder et al.[51], who used a control group that received treatment as usual.

In our study, we found that even participants in the control group participants reported better sleep quality (lower PSQI scores) after the 6-week education-only intervention. This finding is contrary to previous findings of decreased sleep quality during pregnancy in control groups as reported in descriptive studies and RCTs with treatment as usual control groups [52,53]. The improvements could be due to the content of the EOC intervention or to the attention effect associated with completing daily sleep diaries. Such results have implications for understanding the effects of different components of CBT-I (behavioral changes vs diaries). It may suggest that sleep diaries by themselves have some benefit or the sleep hygiene content in the control group modules.

Our findings add to an emerging body of research suggesting that mindfulness-based intervention is an effective strategy helping beyond self-report sleep quality including symptoms such as depression and fatigue during pregnancy. It is possible that mindfulness-based intervention is directly effective for treating depression and fatigue rather than indirect effect from improved sleep. Not only psychological distress but physical symptoms of pregnancy (e.g., fatigue, backache, pain) can also interfere with sleep [54], and mindfulness-based intervention may help these symptoms be less bothersome. However, as we couldn’t find significant differences in actigraphy-measured sleep (e.g., TST, SE, TWT) between groups, further studies are needed to replicate this to examine the effect of mindfulness on objectively measured sleep in pregnant women. Considering that standard CBT-I has minimal effects on rumination and less effective for patients with increased pre-sleep arousal [11,12], mindfulness-based interventions could be alternative to improve insomnia symptoms as well as comorbid symptoms of pregnancy.

Prior studies on the effect of mindfulness-based intervention [55–57] couldn’t find significant improvement in the level of mindfulness in pregnant women, which is similar to our findings. One study [17] reporting significant improvement of level of mindfulness included higher dose of mindfulness practices and longer period of intervention (three hours of weekly session for 8 weeks) versus early studies [55–58]. This suggests that mindfulness interventions may require longer hours of mindfulness protocol in each session and not always improve level of mindfulness in the short-term [58].

### Strengths and limitations

There are a few limitations to this pilot trial. First, the study sample was quite small, and participants were primarily White, non-Hispanic or Latino, employed and had master’s degree-level education. Due to the absence of demographic diversity in the study sample, it is difficult to evaluate whether and how results may differ between different social groups. Future research should implement targeted recruitment strategies to improve sample diversity, such as engaging community organizations, using multiple recruitment sites, and offering study materials in different languages. Ensuring broader representation will enhance the generalizability of findings. Second, the choice of an EOC group may have diluted the observed intervention effects. Unlike an active comparator, an EOC group does not control factors such as participant engagement or placebo effects, which could have influenced the results. Third, this study did not include long-term follow-up assessments, limiting the ability to determine the sustained benefits of the intervention. While our findings suggest preliminary efficacy in improving sleep outcomes, the durability of these effects beyond the immediate post-intervention period remains unclear. Future longitudinal studies should assess whether the observed improvements persist over time. Additionally, given the limited sample size of this pilot study, the p-values reported should be interpreted with caution, as the study may be underpowered to detect smaller effect sizes. Fourth, our analysis did not consider participants’ self-reported nighttime pregnancy symptoms interfering with sleep although there is evidence that pregnancy symptoms such as contractions, and having a full bladder interfere with sleep quality [52]. Additional studies are needed to better understand the association between nighttime pregnancy symptoms and their effects on sleep quality and the efficacy of the OPTIMISM program. Despite these limitations, this study has several strengths to be noted. OPTIMISM was delivered remotely, allowing the trial to continue even when the COVID-19 pandemic struck with no increased dropout rates. Although in-person interventions may have advantages such as promoting social connectedness, the benefits of digital interventions include high accessibility scalability.

Pregnancy is an opportunity and a challenge for implementing behavioral interventions to improve sleep quality. Pregnant individuals can have a great interest in improving their health to benefit themselves and their fetuses and newborns. As we saw in this study, some individuals are not able to engage in behavior change interventions, perhaps because of increased stress, life changes, or lack of fit with the style and mode of delivery. Future research should include modifications to OPTIMISM to increase acceptability and feasibility of participation. This intervention incorporated elements of CBT-I, mindfulness, and sleep diaries, all of which could have contributed to sleep improvement. Measuring the role of each component in effecting change will further our understanding of the mechanism of action for improving sleep and inform intervention design. Longitudinal studies should be conducted to determine the sustainability of sleep improvement during the additional strain of nighttime infant feeding.

## Conclusion

This pilot study demonstrates that the OPTIMISM, tailored for pregnant women with insomnia, shows promise in improving sleep quality and reducing psychological distress. These findings contribute to the emerging body of evidence supporting digital mindfulness interventions as a feasible and acceptable option for improving sleep and mental health during pregnancy. Future studies should aim to include larger, more diverse populations and further investigate the mechanisms through which mindfulness-based interventions impact sleep outcomes in this population.

## Supporting information

S1 File OPTIMISM IRB Protocol 1.27.20.
(DOCX)

S2 File OPTIMISM IRB Protocol 8.13.18 (1).
(DOCX)

S3 File OPTIMISM IRB Protocol 11.15.19 (1).
(DOCX)

S4 File S4 TIDieR checklist. (DOCX) S5 File. S5 CONSORT checklist. (DOCX)

## Abbreviations

CBT-I: cognitive behavioral therapy for insomnia
EPDS: Edinburgh postnatal depression scale
EOC: education-only control
FFMQ: five factor mindfulness questionnaire
OPTIMISM: online prenatal trial in mindfulness sleep management
PROMIS: patient-reported outcomes measurement information system
SE: sleep efficiency
SF-36: short form health survey
SOL: sleep onset latency
SPAQ: sleep problem acceptance questionnaire
TST: total sleep time
TWT: total wake time
WASO: wake after sleep onset

## References

[1] American Psychiatric Association. Diagnostic and statistical manual of mental disorders, Text Revision Dsm-5-tr. 5th ed. Washington, DC: Amer Psychiatric Pub Inc; 2022. p. 1142.

[2] Sedov ID, Anderson NJ, Dhillon AK, Tomfohr-Madsen LM. Insomnia symptoms during pregnancy: a meta-analysis. J Sleep Res. 2021;30(1):e13207. doi: 10.1111/jsr.13207 33140514

[3] Salari N, Darvishi N, Khaledi-Paveh B, Vaisi-Raygani A, Jalali R, Daneshkhah A, et al. A systematic review and meta-analysis of prevalence of insomnia in the third trimester of pregnancy. BMC Pregnancy Childbirth. 2021;21(1):284. doi: 10.1186/s12884-021-03755-z 33836686 PMC8034118

[4] Lu Q, Zhang X, Wang Y, Li J, Xu Y, Song X, et al. Sleep disturbances during pregnancy and adverse maternal and fetal outcomes: a systematic review and meta-analysis. Sleep Med Rev. 2021;58:101436. doi: 10.1016/j.smrv.2021.101436 33571887

[5] Blair LM, Porter K, Leblebicioglu B, Christian LM. Poor sleep quality and associated inflammation predict preterm birth: heightened risk among African Americans. Sleep. 2015 Aug 1;38(8):1259–67. 25845693 10.5665/sleep.4904 PMC4507731

[6] Rawal S, Hinkle SN, Zhu Y, Albert PS, Zhang C. A longitudinal study of sleep duration in pregnancy and subsequent risk of gestational diabetes: findings from a prospective, multiracial cohort. Am J Obstet Gynecol. 2017;216(4):399.e1–e8. doi: 10.1016/j.ajog.2016.11.1051 27939328 PMC6331053

[7] Tomfohr LM, Buliga E, Letourneau NL, Campbell TS, Giesbrecht GF. Trajectories of sleep quality and associations with mood during the perinatal period. Sleep. 2015 Aug 1;38(8):1237–45. 25845691 10.5665/sleep.4900 PMC4507729

[8] Okun ML, Mancuso RA, Hobel CJ, Schetter CD, Coussons-Read M. Poor sleep quality increases symptoms of depression and anxiety in postpartum women. J Behav Med. 2018;41(5):703–10. doi: 10.1007/s10865-018-9950-7 30030650 PMC6192841

[9] Mourady D, Richa S, Karam R, Papazian T, Hajj Moussa F, El Osta N, et al. Associations between quality of life, physical activity, worry, depression and insomnia: a cross-sectional designed study in healthy pregnant women. PLoS One. 2017;12(5):e0178181. doi: 10.1371/journal.pone.0178181 28542529 PMC5439948

[10] Qaseem A, Kansagara D, Forciea MA, Cooke M, Denberg TD, Clinical Guidelines Committee of the American College of Physicians. Management of chronic insomnia disorder in adults: a clinical practice guideline from the American College of Physicians. Ann Intern Med. 2016 Jul 19;165(2):125–33. 27136449 10.7326/M15-2175

[11] Wu JQ, Appleman ER, Salazar RD, Ong JC. Cognitive behavioral therapy for insomnia comorbid with psychiatric and medical conditions: a meta-analysis. JAMA Intern Med. 2015;175(9):1461–72. doi: 10.1001/jamainternmed.2015.3006 26147487

[12] Kalmbach DA, Cheng P, Sangha R, O’Brien LM, Swanson LM, Palagini L, et al. Insomnia, short sleep, and snoring in mid-to-late pregnancy: disparities related to poverty, race, and obesity. Nat Sci Sleep. 2019;11:301–15. doi: 10.2147/NSS.S226291 31807103 PMC6839586

[13] Ong JC, Moore C. What do we really know about mindfulness and sleep health? Curr Opin Psychol. 2020;34:18–22. doi: 10.1016/j.copsyc.2019.08.020 31539830

[14] Ree M, Junge M, Cunnington D. Australasian Sleep Association position statement regarding the use of psychological/behavioral treatments in the management of insomnia in adults. Sleep Med. 2017;36 Suppl 1:S43–7. doi: 10.1016/j.sleep.2017.03.017 28648226

[15] Ong JC, Smith CE. Using mindfulness for the treatment of insomnia. Curr Sleep Med Rep. 2017;3(2):57–65. doi: 10.1007/s40675-017-0068-1 30294523 PMC6171769

[16] Lever Taylor B, Cavanagh K, Strauss C. The effectiveness of mindfulness-based interventions in the perinatal period: a systematic review and meta-analysis. PLoS One. 2016;11(5):e0155720. doi: 10.1371/journal.pone.0155720 27182732 PMC4868288

[17] Pan W-L, Chang C-W, Chen S-M, Gau M-L. Assessing the effectiveness of mindfulness-based programs on mental health during pregnancy and early motherhood - a randomized control trial. BMC Pregnancy Childbirth. 2019;19(1):346. doi: 10.1186/s12884-019-2503-4 31601170 PMC6785846

[18] Zhang X, Lin P, Sun J, Sun Y, Shao D, Cao D, et al. Prenatal stress self-help mindfulness intervention via social media: a randomized controlled trial. J Ment Health. 2023;32(1):206–15. doi: 10.1080/09638237.2021.1952947 34264775

[19] Kanen J, Nazir R, Sedky K, Pradhan B. The effects of mindfulness-based interventions on sleep disturbance: a meta-analysis. APS. 2015;5(2):105–15. doi: 10.2174/2210676605666150311222928

[20] Abatemarco DJ, Gannon M, Short VL, Baxter J, Metzker KM, Reid L, et al. Mindfulness in pregnancy: a brief intervention for women at risk. Matern Child Health J. 2021;25(12):1875–83. doi: 10.1007/s10995-021-03243-y 34618309

[21] Dhillon A, Sparkes E, Duarte RV. Mindfulness-based interventions during pregnancy: a systematic review and meta-analysis. Mindfulness (N Y). 2017;8(6):1421–37. doi: 10.1007/s12671-017-0726-x 29201244 PMC5693962

[22] Smith RB, Mahnert ND, Foote J, Saunders KT, Mourad J, Huberty J. Mindfulness effects in obstetric and gynecology patients during the Coronavirus Disease 2019 (COVID-19) pandemic: a randomized controlled trial. Obstet Gynecol. 2021;137(6):1032–40. doi: 10.1097/AOG.0000000000004316 33957663 PMC8132566

[23] Sun Y, Li Y, Wang J, Chen Q, Bazzano AN, Cao F. Effectiveness of smartphone-based mindfulness training on maternal perinatal depression: randomized controlled trial. J Med Internet Res. 2021;23(1):e23410. doi: 10.2196/23410 33502326 PMC7875700

[24] Dimidjian S, Goodman SH, Felder JN, Gallop R, Brown AP, Beck A. An open trial of mindfulness-based cognitive therapy for the prevention of perinatal depressive relapse/recurrence. Arch Womens Ment Health. 2015;18(1):85–94. doi: 10.1007/s00737-014-0468-x 25298253

[25] Guardino CM, Dunkel Schetter C, Bower JE, Lu MC, Smalley SL. Randomised controlled pilot trial of mindfulness training for stress reduction during pregnancy. Psychol Health. 2014;29(3):334–49. doi: 10.1080/08870446.2013.852670 24180264 PMC4160533

[26] Müller M, Matthies LM, Goetz M, Abele H, Brucker SY, Bauer A, et al. Effectiveness and cost-effectiveness of an electronic mindfulness-based intervention (eMBI) on maternal mental health during pregnancy: the mindmom study protocol for a randomized controlled clinical trial. Trials. 2020;21(1):933. doi: 10.1186/s13063-020-04873-3 33203471 PMC7672841

[27] Mefrouche ML, Siegmann E-M, Böhme S, Berking M, Kornhuber J. The effect of digital mindfulness interventions on depressive, anxiety, and stress symptoms in pregnant women: a systematic review and meta-analysis. Eur J Investig Health Psychol Educ. 2023;13(9):1694–706. doi: 10.3390/ejihpe13090122 37754461 PMC10529137

[28] Kantrowitz-Gordon I, McCurry SM, Landis CA, Lee R, Wi D. Online prenatal trial in mindfulness sleep management (OPTIMISM): protocol for a pilot randomized controlled trial. Pilot Feasibility Stud. 2020;6:128. doi: 10.1186/s40814-020-00675-1 32944276 PMC7488736

[29] Harris PA, Taylor R, Minor BL, Elliott V, Fernandez M, O’Neal L, et al. The REDCap consortium: building an international community of software platform partners. J Biomed Inform. 2019;95:103208. doi: 10.1016/j.jbi.2019.103208 31078660 PMC7254481

[30] Balderson BH, McCurry SM, Vitiello MV, Shortreed SM, Rybarczyk BD, Keefe FJ, et al. Information without implementation: a practical example for developing a best practice education control group. Behav Sleep Med. 2016;14(5):514–27. doi: 10.1080/15402002.2015.1036271 26485203 PMC4838558

[31] Buysse DJ, Reynolds CF 3rd, Monk TH, Berman SR, Kupfer DJ. The Pittsburgh Sleep Quality Index: a new instrument for psychiatric practice and research. Psychiatry Res. 1989;28(2):193–213. doi: 10.1016/0165-1781(89)90047-4 2748771

[32] Data Dictionary [Internet]. Common data repository for nursing science. [cited 2023 Oct 25]. Available from: https://cdrns.nih.gov/

[33] Buysse DJ, Yu L, Moul DE, Germain A, Stover A, Dodds NE, et al. Development and validation of patient-reported outcome measures for sleep disturbance and sleep-related impairments. Sleep. 2010;33(6):781–92. doi: 10.1093/sleep/33.6.781 20550019 PMC2880437

[34] Cella D, Riley W, Stone A, Rothrock N, Reeve B, Yount S, et al. The Patient-Reported Outcomes Measurement Information System (PROMIS) developed and tested its first wave of adult self-reported health outcome item banks: 2005-2008. J Clin Epidemiol. 2010;63(11):1179–94. doi: 10.1016/j.jclinepi.2010.04.011 20685078 PMC2965562

[35] Pilkonis PA, Choi SW, Reise SP, Stover AM, Riley WT, Cella D, et al. Item banks for measuring emotional distress from the Patient-Reported Outcomes Measurement Information System (PROMIS®): depression, anxiety, and anger. Assessment. 2011;18(3):263–83. doi: 10.1177/1073191111411667 21697139 PMC3153635

[36] Cox JL, Holden JM, Sagovsky R. Detection of postnatal depression. Development of the 10-item Edinburgh Postnatal Depression Scale. Br J Psychiatry. 1987;150:782–6. doi: 10.1192/bjp.150.6.782 3651732

[37] Kozinszky Z, Dudas RB. Validation studies of the Edinburgh Postnatal Depression Scale for the antenatal period. J Affect Disord. 2015 May 1;176:95–105. 25704562 10.1016/j.jad.2015.01.044

[38] Salsman JM, Victorson D, Choi SW, Peterman AH, Heinemann AW, Nowinski C, et al. Development and validation of the positive affect and well-being scale for the neurology quality of life (Neuro-QOL) measurement system. Qual Life Res Int J Qual Life Asp Treat Care Rehabil. 2013 Nov;22(9):2569–80. 10.1007/s11136-013-0382-0 PMC3855608 23526093

[39] McHorney CA, Ware JE Jr, Lu JF, Sherbourne CD. The MOS 36-item Short-Form Health Survey (SF-36): III. Tests of data quality, scaling assumptions, and reliability across diverse patient groups. Med Care. 1994;32(1):40–66. doi: 10.1097/00005650-199401000-00004 8277801

[40] Gruber-Baldini AL, Velozo C, Romero S, Shulman LM. Validation of the PROMIS® measures of self-efficacy for managing chronic conditions. Qual Life Res. 2017;26(7):1915–24. doi: 10.1007/s11136-017-1527-3 28239781 PMC5479750

[41] Bothelius K, Jernelöv S, Fredrikson M, McCracken LM, Kaldo V. Measuring acceptance of sleep difficulties: the development of the Sleep Problem Acceptance Questionnaire. Sleep. 2015 Nov 1;38(11):1815–22. 26085302 10.5665/sleep.5170 PMC4813362

[42] Fleury J. The index of self-regulation: development and psychometric analysis. J Nurs Meas. 1998;6(1):3–17. doi: 10.1891/1061-3749.6.1.3 9769608

[43] Bohlmeijer E, ten Klooster PM, Fledderus M, Veehof M, Baer R. Psychometric properties of the five facet mindfulness questionnaire in depressed adults and development of a short form. Assessment. 2011;18(3):308–20. doi: 10.1177/1073191111408231 21586480

[44] Kantrowitz-Gordon I Factor structure and external validity of the Five Facet Mindfulness Questionnaire in pregnancy Mindfulness. 2018 Feb 1;9(1);243–57.

[45] Price C, Kantrowitz-Gordon I, Calhoun R. A pilot feasibility study of mindfulness childbirth education for women with a history of sexual trauma. Complement Ther Clin Pract. 2019 Nov 1;37:102–8. 31563834 10.1016/j.ctcp.2019.09.005

[46] Elo S, Kyngäs H. The qualitative content analysis process. J Adv Nurs. 2008 Apr;62(1):107–15. 18352969 10.1111/j.1365-2648.2007.04569.x

[47] Okun ML, Kohl V, Feliciano L. Comparison of longitudinal diary and actigraphy-assessed sleep in pregnant women. Sleep Med. 2021;88:149–56. doi: 10.1016/j.sleep.2021.09.015 34753041

[48] Valko PO, Hunziker S, Graf K, Werth E, Baumann CR. Sleep-wake misperception. a comprehensive analysis of a large sleep lab cohort. Sleep Med. 2021;88:96–103. doi: 10.1016/j.sleep.2021.10.023 34742039

[49] Mitchell LJ, Bisdounis L, Ballesio A, Omlin X, Kyle SD. The impact of cognitive behavioural therapy for insomnia on objective sleep parameters: a meta-analysis and systematic review. Sleep Med Rev. 2019;47:90–102. doi: 10.1016/j.smrv.2019.06.002 31377503

[50] Manber R, Bei B, Simpson N, Asarnow L, Rangel E, Sit A, et al. Cognitive behavioral therapy for prenatal insomnia: a randomized controlled trial. Obstet Gynecol. 2019;133(5):911–9. doi: 10.1097/AOG.0000000000003216 30969203 PMC6485299

[51] Felder JN, Epel ES, Neuhaus J, Krystal AD, Prather AA. Efficacy of digital cognitive behavioral therapy for the treatment of insomnia symptoms among pregnant women: a randomized clinical trial. JAMA Psychiatry. 2020;77(5):484–92. doi: 10.1001/jamapsychiatry.2019.4491 31968068 PMC6990703

[52] Mindell JA, Cook RA, Nikolovski J. Sleep patterns and sleep disturbances across pregnancy. Sleep Med. 2015;16(4):483–8. doi: 10.1016/j.sleep.2014.12.006 25666847

[53] Kim KT, Cho YW, Bae JG. Quality of sleep and quality of life measured monthly in pregnant women in South Korea. Sleep Breath. 2020;24(3):1219–22. doi: 10.1007/s11325-020-02041-0 32157477

[54] Kamysheva E, Skouteris H, Wertheim EH, Paxton SJ, Milgrom J. A prospective investigation of the relationships among sleep quality, physical symptoms, and depressive symptoms during pregnancy. J Affect Disord. 2010;123(1–3):317–20. doi: 10.1016/j.jad.2009.09.015 19822370

[55] Beattie J, Hall H, Biro MA, East C, Lau R. Effects of mindfulness on maternal stress, depressive symptoms and awareness of present moment experience: a pilot randomised trial. Midwifery. 2017;50:174–83. doi: 10.1016/j.midw.2017.04.006 28463789

[56] Matvienko-Sikar K, Dockray S. Effects of a novel positive psychological intervention on prenatal stress and well-being: a pilot randomised controlled trial. Women Birth. 2017;30(2):e111–8. doi: 10.1016/j.wombi.2016.10.003 27810284

[57] Woolhouse H, Mercuri K, Judd F, Brown SJ. Antenatal mindfulness intervention to reduce depression, anxiety and stress: a pilot randomised controlled trial of the MindBabyBody program in an Australian tertiary maternity hospital. BMC Pregnancy Childbirth. 2014 Oct 25;14:369. 25343848 10.1186/s12884-014-0369-z PMC4215015

[58] Lucena L, Frange C, Pinto ACA, Andersen ML, Tufik S, Hachul H. Mindfulness interventions during pregnancy: a narrative review. J Integr Med. 2020;18(6):470–7. doi: 10.1016/j.joim.2020.07.007 32798196

