# Supplementary material for: Efficacy of online mindfulness for the treatment of insomnia in pregnancy: A randomized clinical trial
Source: PLoS One. 2025 May 9;20(5):e0322931. doi: 10.1371/journal.pone.0322931 (PMC12063803; doi:10.1371/journal.pone.0322931)
Supplement: S1 File — OPTIMISM IRB Protocol 1.27.20. (DOCX) [file pone.0322931.s001.docx]

|  | ***ZIPLINE* APPLICATION: IRB Protocol** |
| --- | --- |
|  | |
|  | |

| **INSTRUCTIONS** |
| --- |
| - **If you are requesting a determination** about whether your activity is human subjects research or qualifies for exempt status, you may skip all questions except those marked with a . For example **1.1** must be answered. - **Answer all questions**. If a question is not applicable to your research or if you believe you have already answered a question elsewhere in the application, state “NA” (and if applicable, refer to the question where you provided the information). If you do not answer a question, the IRB does not know whether the question was overlooked or whether it is not applicable. This may result in unnecessary “back and forth” for clarification. Use non-technical language as much as possible. - To check a box, place an “X” in the box. To fill in a text box, make sure your cursor is within the gray text box bar before typing or pasting text. - The word “you” refers to the researcher and all members of the research team, unless otherwise specified. - For collaborative research, describe only the information that is relevant to you unless you are requesting that the UW IRB provide the review and oversight for your collaborators as well. - You may reference other documents (such as a grant application) if they provide the requested information in non-technical language. Be sure to provide the document name, page(s), and specific sections, and upload it to ***Zipline***. Also, describe any changes that may have occurred since the document was written (for example, changes that you’ve made during or after the grant review process). In some cases, you may need to provide additional details in the answer space as well as referencing a document. |

| **INDEX** | | |
| --- | --- | --- |
| **1** [Overview](#Overview) | **6** [Children (Minors) and Parental Permission](#ChildrenPermission) | **10** [Risk / Benefit Assessment](#RiskBenefit) |
| **2** [Participants](#Participants) | **7** [Assent of Children (Minors)](#Assent) | **11** [Economic Burden to Participants](#EconomicBurden) |
| **3** [Non-UW Research Setting](#International) | **8** [Consent of Adults](#Consent) | **12** [Resources](#Resources) |
| **4** [Recruiting and Screening Participants](#RecruitScreen) | **9** [Privacy and Confidentiality](#Privacy) | **13** [Other Approvals, Permissions, and Regulatory Issues](#Other) |
| **5** [Procedures](#Procedures) |  |  |

| **1 OVERVIEW** | | | | | | | | | | | | | | | | | | | |
| --- | --- | --- | --- | --- | --- | --- | --- | --- | --- | --- | --- | --- | --- | --- | --- | --- | --- | --- | --- |
| **Study Title:** | | | | | | | | | | | | | | | Online Prenatal Trial in Mindfulness Sleep Management (OPTIMISM) | | | |  |
| **1.1 Home institution**. Identify the institution through which the lead researcher listed on the IRB application will conduct the research. Provide any helpful explanatory information. | | | | | | | | | | | | | | | | | | | |
| *In general, the home institution is the institution (1) that provides the researcher’s paycheck and that considers him/her to be a paid employee, or (2) at which the researcher is a matriculated student. Scholars, faculty, fellows, and students who are visiting the UW and who are the lead researcher: identify your home institution and describe the purpose and duration of your UW visit, as well as the UW department/center with which you are affiliated while at the UW.*  *Note that many UW clinical faculty members are paid employees of non-UW institutions.*  *The UW IRB provides IRB review and oversight for only those researchers who meet the criteria described in the* ***POLICY: Use of the UW IRB****.* | | | | | | | | | | | | | | | | | | | |
|  | University of Washington School of Nursing | | | | | | | | | | | | | | | | | | |
| **1.2 Consultation history**. Have you consulted with anyone at HSD about this study? | | | | | | | | | | | | | | | | | | | |
| *It is not necessary to obtain advance consultation. If you have: answering this question will help ensure that the IRB is aware of and considers the advice and guidance you were provided.* | | | | | | | | | | | | | | | | | | | |
|  | **x** | | | **No** | | | | | | |  | | | | | | | | |
|  |  | | | **Yes** | | | | | | | 🡪 If yes, briefly describe the consultation: approximate date, with whom, and method (e.g., by email, phone call, in-person meeting). | | | | | | | | |
|  |  | | |  | | | | | | |  |  |  |  |  |  |  |  |  |
|  | | | | | | | | | | | | | | |  | | | | |
| **1.3 Similar and/or related studies**. Are there any related IRB applications that provide context for the proposed activities? | | | | | | | | | | | | | | | | | | | |
| *Examples of studies for which there is likely to be a related IRB application: Using samples or data collected by another study; recruiting subjects from a registry established by a colleague’s research activity; conducting Phase 2 of a multi-part project, or conducting a continuation of another study; serving as the data coordinating center for a multi-site study that includes a UW site.*  *Providing this information (if relevant) may significantly improve the efficiency and consistency of the IRB’s review.* | | | | | | | | | | | | | | | | | | | |
|  | | **x** | | | | **No** | | | | | | | |  | | | | | |
|  | |  | | | | **Yes** | | | | | | | | 🡪 If yes, briefly describe the other studies or applications and how they relate to the proposed activities. If the other applications were reviewed by the UW IRB, please also provide: the UW IRB number, the study title, and the lead researcher’s name. | | | | | |
|  | |  | | | | | | | | | | | |  |  |  |  |  |  |
|  | | | | | | | | | | | | | | | | |  | | |
| **1.4** **Externally-imposed urgency or time deadlines**. Are there any externally-imposed deadlines or urgency that affect your proposed activity? | | | | | | | | | | | | | | | | | | | |
| *HSD recognizes that everyone would like their IRB applications to be reviewed as quickly as possible. To ensure fairness, it is HSD policy to review applications in the order in which they are received. However, HSD will assign a higher priority to research with externally-imposed urgency that is beyond the control of the researcher. Researchers are encouraged to communicate as soon as possible with their HSD staff contact person when there is an urgent situation (in other words, before submitting the IRB application). Examples: a researcher plans to test an experimental vaccine that has just been developed for a newly emerging epidemic; a researcher has an unexpected opportunity to collect data from students when the end of the school year is only four weeks away.*  *HSD may ask for documentation of the externally-imposed urgency. A higher priority should not be requested to compensate for a researcher’s failure to prepare an IRB application in a timely manner. Note that IRB review requires a certain minimum amount of time; without sufficient time, the IRB may not be able to review and approve an application by a deadline.* | | | | | | | | | | | | | | | | | | | |
|  | | | **x** | | | | | **No** | | | |  | | | | | | | |
|  | | |  | | | | | **Yes** | | | | 🡪 If yes, briefly describe the urgency or deadline as well as the reason for it. | | | | | | | |
|  | | |  | | | |  | | | | | | | | |  | | | |
| **1.5 Objectives** Using lay language, describe the purpose, specific aims, or objectives that will be met by this specific project. If hypotheses are being tested, describe them. You will be asked to describe the specific procedures in a later section.  If your application involves the use of a HUD “humanitarian” device: describe whether the use is for “on-label” clinical patient care, “off-label” clinical patient care, and/or research (collecting safety and/or effectiveness data). | | | | | | | | | | | | | | | | | | | |
|  | | The **purpose** of this study is to test the feasibility and estimate the efficacy of an online mindfulness meditation intervention to help pregnant women with a history of depression to self-manage sleep.  **The specific aims are to:**   1. To identify the feasibility and acceptability of the Online Prenatal Trial in Mindfulness   Sleep Management (OPTIMISM) intervention to improve sleep in pregnant women at risk for  depression. Adherence to the online intervention will be assessed through rates of completion of the  educational components, participation in daily meditation activities, and completion of data collection.  Acceptability will be determined through participant feedback after program completion (satisfaction,  perceived benefits, and barriers) and usability testing of the intervention interface.   1. To estimate the efficacy of an online intervention to improve sleep and depression   symptoms in pregnant women with history of major depression. Our hypothesis is that the 6-week  mobile intervention will show significantly greater improvements in sleep quality and depression  symptoms compared to education control. Using a randomized study design, this pilot study will  provide preliminary estimates of intervention effect size magnitude in pregnant women on the following  outcomes:   1. The primary study outcome of global sleep quality will be measured at baseline and post-intervention and compared between the intervention and education-only control. 2. The secondary sleep and mood outcomes of actigraphy and self-reported sleep and mood will be measured at baseline and post-intervention and compared between the intervention and education-only control. 3. Exploratory measures of self-efficacy, activation, motivation, and mindfulness will be compared pre and post intervention between the intervention and education-only control. | | | | | | | | | | | | | | | | | |
| **1.6** **Study design**. Provide a one-sentence description of the general study design and/or type of methodology. | | | | | | | | | | | | | | | | | | | |
| *Your answer will help HSD in assigning applications to reviewers and in managing workload. Examples: a longitudinal observational study; a double-blind, placebo-controlled randomized study; ethnographic interviews; web scraping from a convenience sample of blogs; medical record review; coordinating center for a multi-site study.* | | | | | | | | | | | | | | | | | | | |
|  | | Randomized controlled study of an online mindfulness sleep intervention compared to education-only control. | | | | | | | | | | | | | | | | | |
| **1.7 Intent**. Check all the descriptors that apply to your activity. You must place an “X” in at least one box. | | | | | | | | | | | | | | | | | | | |
| *This question is essential for ensuring that your application is correctly reviewed. Please read each option carefully.* | | | | | | | | | | | | | | | | | | | |
|  | | **Descriptor** | | | | | | | | | | | | | | | | | |
|  | |  | | | 1. Class project or other activity whose purpose is to provide an educational experience for the researcher (for example, to learn about the process or methods of doing research). | | | | | | | | | | | | | | |
|  | |  | | |  |  |  |  |  |  |  |  |  |  |  |  |  |  |  |
|  | |  | | |  |  |  |  |  |  |  |  |  |  |  |  |  |  |  |
|  | |  | | | 2. Part of an institution, organization, or program’s own internal operational monitoring. | | | | | | | | | | | | | | |
|  | |  | | |  |  |  |  |  |  |  |  |  |  |  |  |  |  |  |
|  | |  | | |  |  |  |  |  |  |  |  |  |  |  |  |  |  |  |
|  | |  | | | 3. Improve the quality of service provided by a specific institution, organization, or program. | | | | | | | | | | | | | | |
|  | |  | | |  |  |  |  |  |  |  |  |  |  |  |  |  |  |  |
|  | |  | | |  |  |  |  |  |  |  |  |  |  |  |  |  |  |  |
|  | |  | | | 4. Designed to expand the knowledge base of a scientific discipline or other scholarly field of study, and produce results that:   - Are expected to be applicable to a larger population beyond the site of data collection or the specific subjects studied, or - Are intended to be used to develop, test, or support theories, principles, and statements of relationships, or to inform policy beyond the study. | | | | | | | | | | | | | | |
|  | | **x** | | |  |  |  |  |  |  |  |  |  |  |  |  |  |  |  |
|  | |  | | |  |  |  |  |  |  |  |  |  |  |  |  |  |  |  |
|  | |  | | | 5. Focus directly on the specific individuals about whom the information or biospecimens are collected through oral history, journalism, biography, or historical scholarship activities, to provide an accurate and evidence-based portrayal of the individuals. | | | | | | | | | | | | | | |
|  | |  | | |  |  |  |  |  |  |  |  |  |  |  |  |  |  |  |
|  | |  | | |  |  |  |  |  |  |  |  |  |  |  |  |  |  |  |
|  | |  | | | 6. A quality improvement or program improvement activity conducted to improve the implementation (delivery or quality) of an accepted practice, or to collect data about the implementation of the practice for clinical, practical, or administrative purposes. This does not include the evaluation of the efficacy of different accepted practices, or a comparison of their efficacy. | | | | | | | | | | | | | | |
|  | |  | | |  |  |  |  |  |  |  |  |  |  |  |  |  |  |  |
|  | |  | | |  |  |  |  |  |  |  |  |  |  |  |  |  |  |  |
|  | |  | | | 7. Public health surveillance activities conducted, requested, or authorized by a public health authority for the sole purpose of identifying or investigating potential public health signals or timely awareness and priority setting during a situation that threatens public health. | | | | | | | | | | | | | | |
|  | |  | | |  |  |  |  |  |  |  |  |  |  |  |  |  |  |  |
|  | |  | | |  |  |  |  |  |  |  |  |  |  |  |  |  |  |  |
|  | |  | | | 8. Preliminary, exploratory, or research development activities (such as pilot and feasibility studies, or reliability/validation testing of a questionnaire) | | | | | | | | | | | | | | |
|  | | **x** | | |  |  |  |  |  |  |  |  |  |  |  |  |  |  |  |
|  | |  | | |  |  |  |  |  |  |  |  |  |  |  |  |  |  |  |
|  | |  | | | 9. Expanded access use of a drug or device not yet approved for this purpose | | | | | | | | | | | | | | |
|  | |  | | |  |  |  |  |  |  |  |  |  |  |  |  |  |  |  |
|  | |  | | |  |  |  |  |  |  |  |  |  |  |  |  |  |  |  |
|  | |  | | | 10. Use of a Humanitarian Use Device | | | | | | | | | | | | | | |
|  | |  | | |  |  |  |  |  |  |  |  |  |  |  |  |  |  |  |
|  | |  | | |  |  |  |  |  |  |  |  |  |  |  |  |  |  |  |
|  | |  | | | 11. Other. Explain: | | | | | | | | | | | | | | |
|  | |  | | |  |  |  |  |  |  |  |  |  |  |  |  |  |  |  |
|  | |  | | |  |  |  |  |  |  |  |  |  |  |  |  |  |  |  |
|  | |  | | |  | | | | | | | |  | | | | | | |
| **1.8** **Background, experience, and preliminary work**. Answer this question **only** if your proposed activity has one or more of the following characteristics. The purpose of this question is to provide the IRB with information that is relevant to its risk/benefit analysis.   - Involves more than minimal risk (physical or non-physical) - Is a clinical trial, or - Involves having the subjects use a drug, biological, botanical, nutritional supplement, or medical device. | | | | | | | | | | | | | | | | | | | |
| *“Minimal risk” means that the probability and magnitude of harm or discomfort anticipated in the research are not greater than those ordinarily encountered in daily life or during the performance of routine physical or psychological examinations or tests.* | | | | | | | | | | | | | | | | | | | |
|  | | **a.** Background. Provide the rationale and the scientific or scholarly background for your proposed activity, based on existing literature (or clinical knowledge). Describe the gaps in current knowledge that your project is intended to address. | | | | | | | | | | | | | | | | | |
|  | | *This should be a plain language description. Do not provide scholarly citations. Limit your answer to less than one page, or refer to an attached document with background information that is no more than three pages long.* | | | | | | | | | | | | | | | | | |
|  | | | | | Poor sleep quality is experienced by 76% of women during pregnancy. Sleep deficiency, including an inadequate amount of sleep and poor sleep quality, has been associated with poor maternal and fetal outcomes, such as gestational diabetes, preterm birth, and cesarean delivery. There is a strong association between sleep quality and depression symptoms during pregnancy and postpartum. A majority of pregnant women prefer non-pharmacological treatments for symptoms like insomnia and depression, possibly because of concerns about fetal or neonatal health during pregnancy and lactation. Mindfulness Based Stress Reduction (MBSR) is a non-pharmacological treatment approach that teaches the ability to pay attention in the moment without judgment through daily meditation practice. MBSR has been shown effective at reducing depressive and anxiety symptoms in multiple contexts including pregnancy and postpartum, but sleep quality was not a key outcome in these studies. The theoretical basis for mindfulness as a self-management strategy for sleep and depression is that principles of mindfulness (acceptance, awareness, and nonjudgment) can reduce sleep-related arousal and rumination that are common both to insomnia and depression. Mindfulness interventions have been correlated with components of self-management, including self-efficacy and intrinsic motivation, and may correlate with increased patient activation. Promising results have been shown with programs that combine elements of MBSR with behavioral strategies to address insomnia, although the evidence for interventions that integrate self-management components to address poor sleep *during pregnancy* is limited. | | | | | | | | | | | | | | |
|  | | **b.** Experience and preliminary work. Briefly describe experience or preliminary work or data (if any) that you or your team have that supports the feasibility and/or safety of this study. | | | | | | | | | | | | | | | | | |
|  | | *It is not necessary to summarize all discussion that has led to the development of the study protocol. The IRB is interested only in short summaries about experiences or preliminary work that suggest the study is feasible and that risks are reasonable relative to the benefits. Examples: You have already conducted a Phase 1 study of an experimental drug which supports the Phase 2 study you are now proposing to do; you have already done a small pilot study showing that the reading skills intervention you plan to use is feasible in an after-school program with classroom aides; you have experience with the type of surgery that is required to implant the study device; you have a study coordinator who is experienced in working with subjects who have significant cognitive impairment.* | | | | | | | | | | | | | | | | | |
|  | |  | | | The PI is a licensed Certified Nurse-Midwife with 20 years of experience caring for pregnant women, and was co-Investigator in a feasibility trial of a classroom-delivered mindfulness-based intervention for pregnant women with history of sexual trauma. That intervention was shown to be feasible and accepted by participants with no reported adverse effects. The PI’s mentoring team has expertise in intervention methodology and sleep research. | | | | | | | | | | | | | | |
| **1.9** **Supplements**. Check all boxes that apply, to identify Supplements you should complete and upload to the **Supporting Documents** SmartForm in ***Zipline***. | | | | | | | | | | | | | | | | | | | |
| *This section is here instead of at the end of the form to reduce the risk of duplicating information in this IRB Protocol form that you will need to provide in these Supplements.* | | | | | | | | | | | | | | | | | | | |
|  | | **Check all That Apply** | | | | | | | | | | | | | **Type of Research** | | | **Supplement Name** | |
|  | |  | | | | | | |  | |  | | | | **Department of Defense**  The research involves Department of Defense funding, facilities, data, or personnel. | | | [*ZIPLINE* SUPPLEMENT: Department of Defense](https://www.washington.edu/research/forms-and-templates/zipline-supplement-department-of-defense-involvement/) | |
|  | |  | | | | | | |  | |  | | | |  |  |  |  |  |
|  | |  | | | | | | |  | |  | | | |  |  |  |  |  |
|  | |  | | | | | | |  | |  | | | | **Department of Energy**  The research involves Department of Energy funding, facilities, data, or personnel. | | | [*ZIPLINE* SUPPLEMENT: Department of Energy](https://www.washington.edu/research/forms-and-templates/zipline-and-paper-supplement-department-of-energy/) | |
|  | |  | | | | | | |  | |  | | | |  |  |  |  |  |
|  | |  | | | | | | |  | |  | | | |  |  |  |  |  |
|  | |  | | | | | |  | |  | | | | | **Drug, biologic, botanical, supplement**  Procedures involve the use of any drug, biologic, botanical or supplement, even if the item is not the focus of your research | | | [*ZIPLINE* SUPPLEMENT: Drugs](https://www.washington.edu/research/forms-and-templates/zipline-supplement-drugs-biologics-botanicals-supplements/) | |
|  | |  | | | | | |  | |  | | | | |  |  |  |  |  |
|  | |  | | | | | |  | |  | | | | |  |  |  |  |  |
|  | |  | | | | | |  | |  | | | | | **Emergency exception to informed consent**  Research that requires this special consent waiver for research involving more than minimal risk | | | [*ZIPLINE* SUPPLEMENT: Exception from Informed Consent for Emergency Research (EFIC)](https://www.washington.edu/research/forms-and-templates/zipline-supplement-exception-from-informed-consent-for-emergency-research-efic/) | |
|  | |  | | | | | |  | |  | | | | |  |  |  |  |  |
|  | |  | | | | | |  | |  | | | | |  |  |  |  |  |
|  | |  | | | | | |  | |  | | | | | **Genomic data sharing**  Genomic data are being collected and will be deposited in an external database (such as the NIH dbGaP database) for sharing with other researchers, and you are asking the UW to provide the required certification or to ensure that the consent forms can be certified | | | [*ZIPLINE* SUPPLEMENT: Genomic Data Sharing](https://www.washington.edu/research/forms-and-templates/zipline-supplement-genomic-data-sharing/) | |
|  | |  | | | | | |  | |  | | | | |  |  |  |  |  |
|  | |  | | | | | |  | |  | | | | |  |  |  |  |  |
|  | |  | | | | | |  | |  | | | | | **Medical device**  Procedures involve the use of any medical device, even if the device is not the focus of your research, except when the device is FDA-approved and is being used through a clinical facility in the manner for which it is approved | | | [*ZIPLINE* SUPPLEMENT: Devices](https://www.washington.edu/research/forms-and-templates/zipline-supplement-devices/) | |
|  | |  | | | | | |  | |  | | | | |  |  |  |  |  |
|  | |  | | | | | |  | |  | | | | |  |  |  |  |  |
|  | |  | | | | | |  | |  | | | | | **Multi-site study**  (You are asking the UW IRB to review one or more sites in a multi-site study.) | | | [*ZIPLINE* SUPPLEMENT: Participating Site in Multi-Site Research](https://www.washington.edu/research/forms-and-templates/zipline-supplement-participating-site-in-multi-site-research/) | |
|  | |  | | | | | |  | |  | | | | |  |  |  |  |  |
|  | |  | | | | | |  | |  | | | | |  |  |  |  |  |
|  | |  | | | | | |  | |  | | | | | **Participant results sharing**  Individual research results will be shared with subjects. | | | [*ZIPLINE* SUPPLEMENT: Participant Results Sharing](https://www.washington.edu/research/forms-and-templates/zipline-supplement-participant-results-sharing/) | |
|  | |  | | | | | | **x** | |  | | | | |  |  |  |  |  |
|  | |  | | | | | |  | |  | | | | |  |  |  |  |  |
|  | |  | | | | | |  | |  | | | | | None of the above | | |  | |
|  | |  | | | | | |  | |  | | | | |  |  |  |  |  |
|  | |  | | | | | |  | |  | | | | |  |  |  |  |  |

| **2 PARTICIPANTS** | | | | | | | | | | | | | | | | | | | | | | |
| --- | --- | --- | --- | --- | --- | --- | --- | --- | --- | --- | --- | --- | --- | --- | --- | --- | --- | --- | --- | --- | --- | --- |
| **2.1 Participants**. Describe the general characteristics of the subject populations or groups, including age range, gender, health status, and any other relevant characteristics. | | | | | | | | | | | | | | | | | | | | | | |
|  | Participants will be pregnant women age ≥ 18 years with self-identified sleep difficulties.  Every effort will be made to recruit racial and ethnic minorities; sleep difficulty in pregnancy affects all races and ethnicities, and may be a pathway toward health disparities in pregnancy outcomes. To ensure inclusion of racial and ethnic minority participants, advertising will particularly target diverse population areas as identified by census tract data in King, Pierce, and Snohomish Counties. | | | | | | | | | | | | | | | | | | | | | |
| **2.2 Inclusion and exclusion criteria.** | | | | | | | | | | | | | | | | | | | | | | |
| **a. Inclusion criteria.** Describe the specific criteria you will use to decide who will be included in your study from among interested or potential subjects. Define any technical terms in lay language. | | | | | | | | | | | | | | | | | | | | | | |
|  | | 1) Viable pregnancy in second trimester (12 - 28 weeks gestation); 2) subjective report of insomnia (score > 7 on the Insomnia Severity Index); 3) age 18 or older; 4) access to an Internet-enabled smartphone, tablet, or computer; and 5) English fluency. Participants will not be excluded from participation if they are currently receiving psychotropic medications or psychotherapy. | | | | | | | | | | | | | | | | | | | | |
| **b. Exclusion criteria.** Describe the specific criteria you will use to decide who will be excluded from your study from subjects who meet the inclusion criteria listed above. Define any technical terms in lay language. | | | | | | | | | | | | | | | | | | | | | | |
|  | | 1) Known severe congenital fetal anomalies, fetal demise, or expected neonatal death; 2) diagnosis of major depressive disorder within past 2 months or current symptoms of depression (score ≥ 3 on the PHQ-2 depression screening questionnaire; 3) other significant psychiatric illness requiring current treatment; 4) current hospitalization; 5) prior diagnosis of obstructive sleep apnea or restless leg syndrome; 6) positive self-report screen for restless leg syndrome or obstructive sleep apnea; 7) regular mindfulness or meditative practice (at least weekly); and 8) regular night-shift work. | | | | | | | | | | | | | | | | | | | | |
| **2.3 Prisoners**. IRB approval is required in order to include prisoners in research, even when prisoners are not an intended target population.  **a.** Will you recruit or obtain data from individuals that you know to be prisoners? | | | | | | | | | | | | | | | | | | | | | | |
| *For records reviews: if the records do not indicate prisoner status and prisoners are not a target population, select “No”. See the* [*WORKSHEET: Prisoners*](https://www.washington.edu/research/forms-and-templates/worksheet-prisoners/) *for the definition of “prisoner”.* | | | | | | | | | | | | | | | | | | | | | | |
|  | | **x** | | | | | **No** | | | |  | | | | | | | | | | | |
|  | |  | | | | | **Yes** | | | | 🡪 If yes, answer the following questions (i – iv). | | | | | | | | | | | |
|  | | | | | | | | | | | i. Describe the type of prisoners, and which prisons/jails: | | | | | | | | | | | |
|  | | | | | | | | | | | | | | |  | | | | | | | |
|  | | | | | | | | | | | ii. One concern about prisoner research is whether the effect of participation on prisoners’ general living conditions, medical care, quality of food, amenities, and opportunity for earnings in prison will be so great that it will make it difficult for prisoners to adequately consider the research risks. What will you do to reduce the chances of this? | | | | | | | | | | | |
|  | | | | | | | | | | | | | | |  | | | | | | | |
|  | | | | | | | | | | | iii. Describe what you will do to make sure that (a) your recruitment and subject selection procedures will be fair to all eligible prisoners and (b) prison authorities or other prisoners will not be able to arbitrarily prevent or require particular prisoners from participating. | | | | | | | | | | | |
|  | | | | | | | | | | | | | | |  | | | | | | | |
|  | | | | | | | | | | | iv. If your research will involve prisoners in federal facilities or in state/local facilities outside of Washington State: check the box below to provide your assurance that you will (a) not encourage or facilitate the use of a prisoner’s participation in the research to influence parole decisions, and (b) clearly inform each prisoner in advance (for example, in a consent form) that participation in the research will have no effect on his or her parole. | | | | | | | | | | | |
|  | | | | | | | | | | | | | | |  | | **Confirmed** | | | | | |
|  | **b.** Is your research likely to have subjects who become prisoners while participating in your study? | | | | | | | | | | | | | | | | | | | | | |
| *For example, a longitudinal study of youth with drug problems is likely to have subjects who will be prisoners at some point during the study.* | | | | | | | | | | | | | | | | | | | | | | |
|  | | | **x** | | | | | **No** | | | | |  | | | | | | | | | |
|  | | |  | | | | | **Yes** | | | | | 🡪 If yes, if a subject becomes a prisoner while participating in your study, will you continue the study procedures and/or data collection while the subject is a prisoner? | | | | | | | | | |
|  | | |  | | | | | | | | | |  |  |  |  |  |  |  |  |  |  |
|  | | | | | | | | | | | | | |  | | **No** | |  | | | | |
|  | | | | | | | | | | | | | |  | | **Yes** | | 🡪 If yes, describe the procedures and/or data collection you will continue with prisoner subjects | | | | |
|  | | | | | | | | | | | | | |  | | | |  |  |  |  |  |
|  | | | | | | | | | | | | | | | | | | |  | | | |
| **2.4 Protected populations**. IRB approval is required for the use of the subject populations listed here. Check the boxes for any of these populations that you will purposefully include in your research. (In other words, being a part of the population is an inclusion criterion for your study.) | | | | | | | | | | | | | | | | | | | | | | |
| *The WORKSHEETS describe the criteria for approval but do not need to be completed and should not be submitted.* | | | | | | | | | | | | | | | | | | | | | | |
|  | **Population** | | | | | | | | | | | | | | | | | | | **Worksheet** | | |
|  |  | | Fetuses in utero | | | | | | | | | | | | | | | | | | [WORKSHEET: Pregnant Women](https://www.washington.edu/research/forms-and-templates/worksheet-pregnant-women/) | |
|  |  | |  |  |  |  |  |  |  |  |  |  |  |  |  |  |  |  |  |  |  |  |
|  |  | |  |  |  |  |  |  |  |  |  |  |  |  |  |  |  |  |  |  |  |  |
|  |  | | Neonates of uncertain viability | | | | | | | | | | | | | | | | | | [WORKSHEET: Neonates](https://www.washington.edu/research/forms-and-templates/worksheet-neonates/) | |
|  |  | |  |  |  |  |  |  |  |  |  |  |  |  |  |  |  |  |  |  |  |  |
|  |  | |  |  |  |  |  |  |  |  |  |  |  |  |  |  |  |  |  |  |  |  |
|  |  | | Non-viable neonates | | | | | | | | | | | | | | | | | | [WORKSHEET: Neonates](https://www.washington.edu/research/forms-and-templates/worksheet-neonates/) | |
|  |  | |  |  |  |  |  |  |  |  |  |  |  |  |  |  |  |  |  |  |  |  |
|  |  | |  |  |  |  |  |  |  |  |  |  |  |  |  |  |  |  |  |  |  |  |
|  |  | | Pregnant women | | | | | | | | | | | | | | | | | | [WORKSHEET: Pregnant Women](https://www.washington.edu/research/forms-and-templates/worksheet-pregnant-women/) | |
|  | **x** | |  |  |  |  |  |  |  |  |  |  |  |  |  |  |  |  |  |  |  |  |
|  |  | |  |  |  |  |  |  |  |  |  |  |  |  |  |  |  |  |  |  |  |  |
| **a.** If you check any of the boxes above, use this space to provide any information you think may be relevant for the IRB to consider. | | | | | | | | | | | | | | | | | | | | | | |
|  | | Behavioral interventions to treat insomnia are considered first-line treatments. The proposed intervention has the potential for direct benefit to the mother in improving sleep and this may indirectly provide benefit to the fetus. Individuals engaged in the research will not be providing prenatal or postnatal care to the participants. No inducements, monetary or otherwise, will be offered to participants to terminate pregnancy. Individuals engaged in the research will have no part in any decisions as to the timing, methods, or decisions to terminate a pregnancy. Individuals engaged in the research will have no part in determining the viability of a neonate. | | | | | | | | | | | | | | | | | | | | |
| **2.5** **Native Americans or non U.S. indigenous populations.** Will you actively recruit from Native American or non-U.S. indigenous populations through a tribe, tribe-focused organization, or similar community-based organization? | | | | | | | | | | | | | | | | | | | | | | |
| *Indigenous people are defined in international or national legislation as having a set of specific rights based on their historical ties to a particular territory and their cultural or historical distinctiveness from other populations that are often politically dominant.*  *Examples: a reservation school or health clinic; recruiting during a tribal community gathering* | | | | | | | | | | | | | | | | | | | | | | |
|  | **x** | | | | **No** | | | |  | | | | | | | | | | | | | |
|  |  | | | | **Yes** | | | | 🡪 If yes, name the tribe, tribal-focused organization, or similar community based organization. The UW IRB expects that you will obtain tribal/indigenous approval before beginning your research. | | | | | | | | | | | | | |
|  |  | | | |  | | | |  |  |  |  |  |  |  |  |  |  |  |  |  |  |
|  | | | | | | | | | | | |  | | | | | | | | | | |
| **2.6** **Third party subjects.** Will you collect private identifiable information about *other individuals* from your subjects? Common examples include: collecting medical history information or contact information about family members, friends, co-workers. | | | | | | | | | | | | | | | | | | | | | | |
| *“Identifiable” means any direct or indirect identifier that, alone or in combination, would allow you or another member of your research team to readily identify the person. For example, suppose that you are studying immigration history. If you ask your subjects several questions about their grandparents but you do not obtain names or other information that would allow you to readily identify the grandparents, then you are not collecting private identifiable information about the grandparents.* | | | | | | | | | | | | | | | | | | | | | | |
|  | **x** | | | | | **No** | | | |  | | | | | | | | | | | | |
|  |  | | | | | **Yes** | | | | 🡪 If yes, these individuals are considered human subjects in your study. Describe them and what data you will collect about them. | | | | | | | | | | | | |
|  |  | | | | |  | | | |  |  |  |  |  |  |  |  |  |  |  |  |  |
|  | | | | | | | | | | | |  | | | | | | | | | | |
| **2.7** **Number of subjects.** Can you predict or describe the maximum number of subjects (or subject units) you need to complete your study, for each subject group? | | | | | | | | | | | | | | | | | | | | | | |
| *Subject units mean units within a group. For most research studies, a group will consist of individuals. However, the unit of interest in some research is not the individual. Examples:*   - *Dyads such as caregiver-and-Alzheimer’s patient, or parent and child* - *Families* - *Other units, such as student-parent-teacher*   *Subject group means categories of subjects that are meaningful for your research. Some research has only one subject group – for example, all UW students taking Introductory Psychology. Some common ways in which subjects are grouped include:*   - *By intervention – for example, an intervention group and a control group.* - *By subject population or setting – for example, urban versus rural families* - *By age – for example, children who are 6, 10, or 14 years old.*   *The IRB reviews the number of subjects you plan to study in the context of risks and benefits. You may submit a Modification to increase this number at any time after you receive IRB approval. If the IRB determines that your research involves no more than minimal risk: you may exceed the approved number and it will not be considered non-compliance. If your research involves more than minimal risk: exceeding the approved number will be considered non-compliance.* | | | | | | | | | | | | | | | | | | | | | | |
|  |  | | | | | **No** | | | 🡪 If no, provide your rationale in the box below. Also, provide any information you can about the scope/size of the research. You do not need to complete the table. | | | | | | | | | | | | | |
|  |  | | | | | | | |  |  |  |  |  |  |  |  |  |  |  |  |  |  |
|  |  | | | | | | | | *Example: you may not be able to predict the number of subjects who will complete an online survey advertised through Craigslist, but you can state that you will post your survey for two weeks and the number who respond is the number who will be in your study.* | | | | | | | | | | | | | |
|  |  | | | | | | | | | | |  | | | | | | | | | | |
|  | **x** | | | **Yes** | | | | | 🡪 If yes, for each subject group, use the table below to provide your estimate of the maximum desired number of individuals (or other subject unit, such as families) who will complete the research. | | | | | | | | | | | | | |
|  |  | | | | | | | |  |  |  |  |  |  |  |  |  |  |  |  |  |  |
|  | **Group name/description** | | | | | | | | | | | | | | | | | | | | | **Maximum desired number of individuals (or other subject unit, such as families) who will complete the research**  ****For clinical trials: provide numbers for your site and for the study-wide total number*** |
|  | Intervention Group (mindfulness) | | | | | | | | | | | | | | | | | | | | | 25 |
|  | Education-only Control Group | | | | | | | | | | | | | | | | | | | | | 25 |
|  |  | | | | | | | | | | | | | | | | | | | | |  |
|  |  | | | | | | | | | | | | | | | | | | | | |  |
|  |  | | | | | | | | | | | | | | | | | | | | |  |
|  |  | | | | | | | | | | | | | | | | | | | | |  |

| **3 NON-UW RESEARCH SETTING**  ***Complete this section only if your research will take place outside of UW and Harborview*** | |
| --- | --- |
| **3.1 Reason for sites**. Describe the reason(s) why you selected the sites where you will conduct the research. | |
|  | n/a |
| **3.2 Local context**. Culturally-appropriate procedures and an understanding of local context are an important part of protecting subjects. Describe any site-specific cultural issues, customs, beliefs, or values that may affect your research or how it is conducted. | |
| *Examples: It would be culturally inappropriate in some international settings for a woman to be directly contacted by a male researcher; instead, the researcher may need to ask a male family member for permission before the woman can be approached. It may be appropriate to obtain permission from community leaders prior to obtaining consent from individual members of a group.*  *This federal site maintains an international list of human research standards and requirements:*  [*http://www.hhs.gov/ohrp/international/index.html*](http://www.hhs.gov/ohrp/international/index.html) | |
|  | n/a |
| **3.3 Site-specific laws**. Describe any local laws that may affect your research (especially the research design and consent procedures). The most common examples are laws about:   - **Specimens** – for example, some countries will not allow biospecimens to be taken out of the country. - **Age of consent** – laws about when an individual is considered old enough to be able to provide consent vary across states, and across countries. - **Legally authorized representative** – laws about who can serve as a legally authorized representative (and who has priority when more than one person is available) vary across states and countries. - **Use of healthcare records** – many states (including Washington State) have laws that are similar to the federal HIPAA law but that have additional requirements. | |
|  | n/a |
| **3.4 Site-specific administrative or ethical requirements**. Describe local administrative or ethical requirements that affect your research. | |
| *Example: A school district may require you to obtain permission from the head district office as well as school principals before approaching teachers or students; a factory in China may allow you to interview factory workers but not allow you to pay them.* | |
|  | n/a |

| **4 RECRUITING and SCREENING PARTICIPANTS** | | | | | | | | | | | | | | | | | |
| --- | --- | --- | --- | --- | --- | --- | --- | --- | --- | --- | --- | --- | --- | --- | --- | --- | --- |
| **4.1 Recruiting and Screening**. Describe how you will identify, recruit, and screen subjects. Include information about: how, when, where, and in what setting. Identify who (by position or role, not name) will approach and recruit subjects, and who will screen them for eligibility. | | | | | | | | | | | | | | | | | |
|  | | **Recruitment**  Recruitment will use several methods: posted recruitment flyers, social media posts and advertisements, and recruitment websites.   - Recruitment flyers will be distributed to community-based prenatal care clinics, high-risk pregnancy clinics (maternal-fetal medicine), maternity service providers (such as First Steps, WIC, Nurse Family Partnership), and perinatal mental health providers. - Social media posts will be made periodically to UW School of Nursing webpage/social media accounts, Facebook, Nextdoor, and Meetup groups. Posts will guide potential participants to the study webpage, phone number, and email. - The study will be posted on research recruitment sites such as ITHS and ClinicalTrials.gov. - We will be use the Biomedical Informatics (BMI) services group affiliated with the ITHS and UW Medicine IT to identify our target population (pregnant women at least 18 years old who are less than 28 weeks pregnant) from within the UW Medicine medical records. They will provide us with basic contact information plus current pregnancy status. We’ll load that information into a dedicated REDCap database and sent out email invitations via the REDCap survey mechanism. The survey will contain an invite to join the study and a link to the online screening survey. We’ll remind potential participants up to 3 times of our study or until they fill out the survey (whatever comes first). The BMI services group will assist with completing regulatory requirements to access the limited protected health information (PHI) of potential participants.   Interested women will contact the PI or research assistant by phone or email or begin the screening process through the study webpage. If interested, potential participants will then be screened for eligibility.  **Screening**  Interested potential participants may choose to visit the study webpage or contact the PI by phone or email. For those who contact the PI by phone or email, the PI will screen potential participants initially over the phone (see Recruitment Script and Section 4.7). For those who are eligible and interested, they will be scheduled for the enrollment/baseline session.  Potential participants who initially visit the study webpage may choose to complete online screening (hosted by REDCap). The screening questions and instruments will be automatically scored by REDCap and will immediately indicate whether participants meet eligibility criteria. Those that are eligible and indicate continued interest will be advised that a member of the study team will contact them to schedule the enrollment/baseline session. | | | | | | | | | | | | | | | |
| **4.2 Recruitment materials**.  **a.** What materials (if any) will you use to recruit and screen subjects? | | | | | | | | | | | | | | | | | |
| *Examples: talking points for phone or in-person conversations; video or audio presentations; websites; social media messages; written materials such as letters, flyers for posting, brochures, or printed advertisements; questionnaires filled out by potential subjects.* | | | | | | | | | | | | | | | | | |
|  | | | | | | Talking points for phone or in-person conversations, recruitment flyers, emails, social media posts and advertisements, and the study webpage will be used to recruit and screen subjects (see Recruitment Script). | | | | | | | | | | | |
| **b.** Upload descriptions of each type of material (or the materials themselves) to the **Consent Forms and Recruitment Materials** SmartForm of ***Zipline***. If you will send letters to the subjects, the letter should include a statement about how you obtained the subject’s name, contact information, and any other subject-specific information (such as a health condition) that is mentioned in the letter. | | | | | | | | | | | | | | | | | |
| *HSD encourages researchers to consider uploading descriptions of most recruitment and screening materials instead of the materials themselves. The goal is to provide the researchers with the flexibility to change some information on the materials without submitting a Modification for IRB approval of the changes. Examples:*   - *You could provide a list of talking points that will be used for phone or in-person conversations instead of a script.* - *For the description of a flyer, you might include the information that it will provide the study phone number and the name of a study contact person (without providing the actual phone number or name). In doing so, you would not need to submit a Modification if/when the study phone number or contact person changes. Also, instead of listing the inclusion/exclusion criteria, you might state that the flyer will list one or a few of the major inclusion/exclusion criteria.* - *For the description of a video or a website, you might include a description of the possible visual elements and a list of the content (e.g., study phone number; study contact person; top three inclusion/exclusion criteria; payment of $50; study name; UW researcher).* | | | | | | | | | | | | | | | | | |
| **4.3 Relationship with participant population**. Do any members of the study team have an existing relationship with the study population(s)? | | | | | | | | | | | | | | | | | |
| *Examples: a study team member may have a dual role with the study population (for example, being their clinical care provider, teacher, laboratory directory or tribal leader in addition to recruiting them for his/her research).* | | | | | | | | | | | | | | | | | |
|  | | | | **x** | | | | **No** |  | | | | | | | | |
|  | | | |  | | | | **Yes** | 🡪 If yes, describe the nature of the relationship. | | | | | | | | |
|  | | | | |  | | |  | | | |  | | | | | |
| **4.4 Payment to participants**. Describe any payment you will provide, including:   - The total amount/value - Whether payment will be “pro-rated” so that participants who are unable to complete the research may still receive some part of the payment | | | | | | | | | | | | | | | | | |
| *The IRB expects the consent process or study information provided to the subjects to include information about the number and amount of payments, and especially the time when subjects can expect to receive payment. One of the most frequent complaints received by HSD is from subjects who expected to receive cash or a check on the day that they completed a study and who were angry or disappointed when payment took 6-8 weeks to reach them.*  *Do not include a description of any expenses that will be reimbursed.* | | | | | | | | | | | | | | | | | |
|  | Participants who agree to participate in the study will receive a monetary incentive of up to $100 (in retail gift cards). The incentives will be divided into two portions such that:   - $50 gift card will be given after the completion of the baseline assessment and actigraphy, at the time point when research staff conduct a home visit to explain the procedures for group assignment (intervention or control) - $50 gift card will be given after completion of the post-intervention assessment and actigraphy, either in-person when retrieving actigraphy equipment or by mail. | | | | | | | | | | | | | | | | |
| **4.5 Non-monetary compensation**. Describe any non-monetary compensation you will provide. Example: extra credit for students; a toy for a child. If you will be offering class credit to students, you must provide (and describe) an alternate way for the students to earn the extra credit without participating in your research. | | | | | | | | | | | | | | | | | |
|  | | | n/a | | | | | | | | | | | | | | |
| **4.6 Will you access or obtain data or specimens for recruiting and screening procedures prior to enrollment?** | | | | | | | | | | | | | | | | | |
| *Examples: names and contact information; the information gathered from records that were screened; results of screening questionnaires or screening blood tests; Protected Health Information (PHI) from screening medical records to identify possible subjects.* | | | | | | | | | | | | | | | | | |
|  | | | | **x** | | | | **No** | 🡪 If no, skip the rest of this section; go to [question **5.1**](#q5point1). | | | | | | | | |
|  | | | | **x** | | | | **Yes** | 🡪 If yes, describe any data and/or specimens (including PHI) you will access or obtain for recruiting and screening and whether you will retain it as part of the study data. | | | | | | | | |
|  | | | | |  | | |  | | | | See 4.1 | | | | | |
| **4.7 Consent for recruiting and screening**. Will you obtain consent for any of the recruiting and screening procedures? ([Section 8: Consent of Adults](#Consent) asks about consent for the main study procedures). | | | | | | | | | | | | | | | | | |
| *“Consent” includes: consent from individuals for their own participation; parental permission; assent from children; consent from a legally authorized representative for adult individuals who are unable to provide consent.*  *Examples:*   - *For a study in which names and contact information will be obtained from a registry: the registry should have consent from the registry participants to release their names and contact information to researchers.* - *For a study in which possible subjects are identified by screening records: there will be no consent process.* - *For a study in which individuals respond to an announcement and call into a study phone line: the study team person talking to the individual may obtain non-written consent to ask eligibility questions over the phone.* | | | | | | | | | | | | | | | | | |
|  | |  | | | | | **No** | | | 🡪 If no, skip the rest of this section; go to [question **5.1**](#q5point1). | | | | | | | |
|  | | **x** | | | | | **Yes** | | | 🡪 If yes, describe the consent process. | | | | | | | |
|  |  | | | | | |  | | | | The study team member talking to potential research participants on the phone or in-person will ask permission from the individual to ask questions about their health before initiating the screening questions described above. | | | | | | |
|  | | | | | | | | | | | **a**. Documentation of consent. Will you obtain a written or verifiable electronic signature from the subject on a consent form to document consent for all of the **recruiting and screening procedures**? | | | | | |  |
|  | | | | | | | | | | | | | **x** | **No** | 🡪 If no, describe the information you will provide during the consent process and for which procedures. | |  |
|  | | | | | | | | | | | | |  |  |  | The research team member conducting the screening interview will explain that questions about the potential participant’s health will be necessary to determine whether she is eligible to participate in the study. The screener will explain that: 1) the information provided will not be connected to any personal identifiers if the potential participant is determined to be ineligible or does not enroll in the study; and 2) if the potential participant is determined to be eligible and completes study enrollment then the screening data will be incorporated into study data. |  |
|  | | | | | | | | | | | | |  | **Yes** | 🡪 If yes, upload the consent form to the **Consent Forms and Recruitment Materials** page of ***Zipline***. | |  |
|  | | | | | | | | | | | | |  |  |  |  |  |

| **5 PROCEDURES** | | | | | | | | | | | | | | | | | | | | | | | | | | | | | | | | | | | | | | | | | | | | | | | | | | |
| --- | --- | --- | --- | --- | --- | --- | --- | --- | --- | --- | --- | --- | --- | --- | --- | --- | --- | --- | --- | --- | --- | --- | --- | --- | --- | --- | --- | --- | --- | --- | --- | --- | --- | --- | --- | --- | --- | --- | --- | --- | --- | --- | --- | --- | --- | --- | --- | --- | --- | --- |
| **5.1** **Study procedures**. Using lay language, provide a complete description of the study procedures, including the sequence, intervention or manipulation (if any), drug dosing information (if any), use of records, time required, and setting/location. If it is available and you think it would be helpful to the IRB: Upload a study flow sheet or table to the **Supporting Documents** SmartForm in ***Zipline***. | | | | | | | | | | | | | | | | | | | | | | | | | | | | | | | | | | | | | | | | | | | | | | | | | | |
| *For studies comparing standards of care: It is important to accurately identify the research procedures. See UW IRB* [***POLICY: Risks of Harm from Standard Care***](https://www.washington.edu/research/policies/risks-of-harm-from-standard-care/) *and the draft guidance from the federal Office of Human Research Protections,* [***“Guidance on Disclosing Reasonably Foreseeable Risks in Research Evaluating Standards of Care”;***](http://www.hhs.gov/ohrp/newsroom/rfc/comstdofcare.html) *October 20, 2014.* | | | | | | | | | | | | | | | | | | | | | | | | | | | | | | | | | | | | | | | | | | | | | | | | | | |
|  | T1: Baseline assessment. Once initial eligibility criteria are met based on telephone screening, participants will schedule a time to meet the PI at their home to discuss the study, answer questions, and provide written informed consent. After receiving consent, verbal and written instructions will be provided for wearing an ActiWatch (wrist actigraphic device) on the non-dominant wrist for 8 consecutive days (7 nights) and participants will commence daily online sleep diaries. A link to the online assessment on REDCap will be sent to participants by email after the home visit. Baseline assessments will include completion of self-reported questionnaires (demographics, pregnancy characteristics, height and weight [pre-pregnant and current], sleep [PSQI and PROMIS measures], and measures of self-management [self-efficacy, motivation, activation], mood [depression, anxiety, positive affect], and general health status). The estimated time to complete the online baseline assessment on REDCap is 60 minutes. After completion of the baseline assessment, eligible women will be block-randomized to receive OPTIMISM or active control. Participants will not be informed about the general content of the other online intervention. After completion of one 8 days and 7 nights of actigraphy data collection, participants will meet with a research assistant to retrieve the Actiwatch and receive instruction in the intervention or control condition. Daily diaries will continue for the duration of the study.  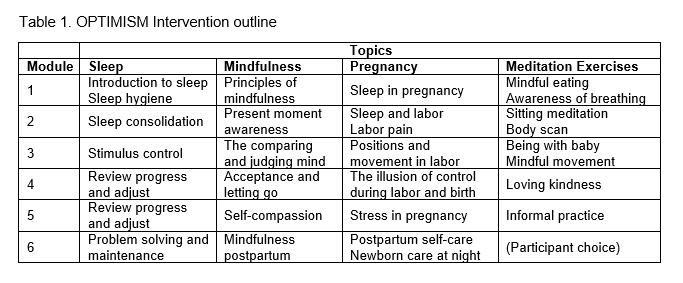Intervention. The OPTIMISM intervention consists of six weekly online self-directed learning modules about mindfulness meditation, sleep challenges in pregnancy, and behavioral strategies to improve sleep (Table 1). Building on experience developing mindfulness interventions for at-risk pregnant populations, the program will adapt elements of two in-person mindfulness-based interventions: Mindfulness-Based Childbirth and Parenting (MBCP) and Mindfulness-Based Therapy for Insomnia (MBTI). The emphasis of intervention activities will be the use of mindful awareness as a sleep self-management technique to increase total sleep time (TST) and sleep efficiency (SE). Intervention activities and assessments will be delivered through Research Electronic Data Capture (REDCap) and linked web-pages. Each weekly module will contain linked didactic content on sleep, pregnancy, and mindfulness meditation using interactive text and video, audio meditations (short and long versions), discussion-board for interaction with a certified Mindfulness instructor (who is not the PI) and other participants, and weekly reflective exercises. Integration of the intervention into REDCap surveys will allow automatic data capture on module completion and access to meditations. Support during daytime hours will be available through text or email communication with the mindfulness instructor. The mindfulness instructor will provide weekly feedback to participants through email about their assignments and progress toward sleep improvement. Participants will also have opportunities to ask questions and provide feedback within each module of the didactic content. Daily reminders about practicing mindfulness and/or short messages to reinforce course content will be sent by text and/or email. Each participant’s progress in the course will be monitored weekly through REDCap, which will provide ongoing feasibility data. Every day during the course participants will complete the CISSM sleep diary on REDCap, modified to include pregnancy symptoms interfering with sleep, along with daily meditation practice. The diary will help participants self-manage their sleep and monitor progress in meeting sleep-related goals. Weekly progress on sleep goals will be calculated and provided to participants by an emailed report and displayed on a dashboard on the intervention website.  Usability testing of the intervention platform and dashboard will be offered as an optional procedure to participants assigned to the intervention group. Usability testing will be scheduled at two times: early during the intervention (after orientation to the website); and during week 5 or 6. The portable Usability Lab (Noldus Information Technology, Lesburg, VA) includes an electronic tablet loaded with the application, Morae recorder software (version 3.3), and a video-camera. This portable system supports synchronous recordings of human-computer interactions, video and audio, high resolution screen capturing, and is operating system independent. We will directly observe (and audio- and video-record) users as they interact with the intervention in their homes or offices. In addition, all users will be asked to verbally describe their actions and strategies when interacting with the online intervention, “thinking aloud” about how and why they make specific choices in their interactions. These interactions include navigating within the intervention website, viewing and understanding the sleep dashboard, and completing online sleep diaries, Participants will also provide feedback on different dashboard mockup designs.  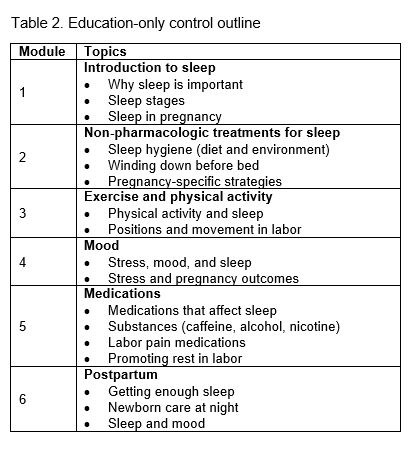Control. The education-only control (EOC) intervention (Table 2) will deliver education on sleep hygiene, pregnancy, and childbirth in a modular format. This will not include any mindfulness content or meditations, sleep scheduling (bed restriction or stimulus control recommendations), reflective exercises, or development of sleep goals. Control participants will complete CISSM sleep diaries and have access to an online discussion board limited to control group participants and an instructor/moderator. Control group participants will not receive feedback from the instructor on any changes in their sleep during the study.  T2: Post-intervention assessment. After intervention completion, participants will complete assessments online through REDCap. The estimated time to complete the baseline assessment on REDCap is 60 minutes. A research assistant will arrange to meet the participant to deliver the Actiwatches for another session of actigraphy monitoring. The participant will for wear an ActiWatch on the non-dominant wrist for 8 consecutive days (7 nights) Sleep diaries will continue through the end of T_2_ actigraphy data collection for Intervention and Control Group participants. After the actigraphy monitoring is complete the research assistant will arrange retrieval of the ActiWatch.  Table 2. Education-only control outline  Postpartum assessment. Approximately four weeks after the expected birth, participants will complete a postpartum questionnaire online through REDCap. The estimated time to complete this assessment will be 15 minutes. The participant will receive an email with a link to the online assessment. At the end of the questionnaire, information about the key differences in content between the two interventions will be provided to all participants. This will include;   - The mindfulness intervention focused on use of mindfulness meditation, sleep restriction, and stimulus control - The active control condition focused on education about sleep and strategies to improve sleep that do NOT include meditation, sleep restriction, or stimulus control.   This will conclude participant's involvement in the research study although they will be provided contact information for research study staff for any further questions or comments. | | | | | | | | | | | | | | | | | | | | | | | | | | | | | | | | | | | | | | | | | | | | | | | | | |
| **5.2 Data variables.** Describe the specific data you will obtain (including a description of the most sensitive items). If you would prefer, you may upload a list of the data variables to the **Supporting Documents** SmartForm instead of describing the variables below. | | | | | | | | | | | | | | | | | | | | | | | | | | | | | | | | | | | | | | | | | | | | | | | | | | |
|  | | | **Prescreening**  Age, gestational age, mental health history (recent diagnosis of major depression, past history of depression, other significant psychiatric illness), Patient Health Questionnaire (PHQ-9), Insomnia Severity Index (ISI), self-report of height and pre-pregnancy and current weight, and snoring frequency.  **Data collected only at baseline (T1)**  Demographic Survey – education, race/ethnicity, parity, household income, marital status, household composition, and sleep partners.  **Data collected at baseline (T1) and post-intervention (T2) assessment**  **Pregnancy health**: current height and weight, gestational age, pregnancy complications, medical and psychiatric co-morbidities, medications, and substance use.  **Pittsburgh Sleep Quality Index (PSQI)** is a 19-item survey to assess sleep quality and usual sleep habits over the previous month.  **PROMIS Sleep Related Impairment – Short Form** has 8 items related to consequences from poor sleep to daily function, on a 5-point Likert-type scale ranging from 1 (not at all) to 5 (very much).  **PROMIS Sleep Disturbance – Short Form** has 8 items related to sleep quality and difficulty falling asleep over the past 7 days, on a 5-point Likert-type scale.  **PROMIS Fatigue -- Short Form** has 8 items related to decreased energy and activity secondary to fatigue over the past 7 days. Items are scored on a 5-point Likert-type scale.  **Edinburgh Postnatal Depression Scale (EPDS)** is a 10-item self-report questionnaire that assesses symptoms of depression in the past 7 days and has been validated for use in pregnancy and postpartum.  **PROMIS Emotional Distress -- Anxiety Short Form** has 8 items on feelings related to anxiety felt over the past 7 days. Items are scored on a 5-point Likert-type scale ranging from 1 (never) to 5 (always).  **PROMIS Positive Affect – SF** has 15 items that reflect a level of pleasurable engagement with the environment, such as happiness, joy, and enthusiasm.  **SF-36** is an indicator of overall health status across vitality, physical functioning, bodily pain, general health perceptions, physical role functioning, emotional role functioning, social role functioning, and mental health..  **Patient Activation Measure** has 13 items that measures a patient’s level of activation (capacity for self-management and involvement) in health care actions and decisions.  **Index of Self-Regulation** has 9 items that measure how a person feels about taking care of themselves.  **PROMIS Self-Efficacy for Managing Emotions SF** has 4 items that measure current level of confidence in handling stress and negative emotions.  **Five Facet Mindfulness Questionnaire (FFMQ) Short Form** is a 24-item measure of mindfulness. Subscales include observing, describing, acting with awareness, nonjudging, and nonreactivity. Higher scores indicate higher levels of mindfulness. The FFMQ-SF has been validated in pregnancy.  **Sleep diaries** (collected daily from baseline through study completion)  Sleep diaries will provide subjective measurement of sleep variables such as bed/rise times, sleep onset latency (SOL), wake after sleep onset (WASO), number of awakenings (NWAK), total sleep time (TST), time in bed (TIB), and sleep efficiency (SE). Sleep diaries will also include questions on sleep schedule; caffeine, nicotine, alcohol, and medication use; frequency, type, and duration of meditation practice (Intervention group).    **Actigraphy**  Wrist actigraphy with Actiwatches (Phillips Respironics, Bend, OR) will be used to measure sleep/wake patterns at home for 8 days (7 nights) at baseline and post-intervention. Sleep variables obtained via actigraphy include sleep onset, sleep offset, sleep period time, mean activity during sleep, mean number of minutes of sleep and wake, percentage of sleep and wake within the sleep period, and number of waking episodes during the sleep period. These variables are used to derive total sleep time, percent sleep efficiency (SE, sleep time/time in bed x 100), percent wake after sleep onset (WASO, wake min/sleep time x 100), and a fragmentation index (of waking episodes/hr).  **Intervention adherence**  Intervention adherence will be measured on REDCap through completion of intervention modules, frequency of meditation practice, and frequency of completion of 8 weeks of daily sleep diary collection in pregnancy.  **Data collected only at post-intervention (T2) assessment**  Intervention acceptability will be measured with questionnaires administered at T2 using 5-point Likert-type scale items about their experiences with using the intervention web-platform and perceived benefits and challenges of the program. Open-ended qualitative questions will solicit general feedback on the intervention, and explanation of quantitative responses. Acceptability questions will focus both on the substance of the intervention as well as Internet delivery and mobile device compatibility.  **Data collected at postpartum assessment**  **Obstetric outcomes** (preterm birth, mode of delivery, NICU admission, birth weight, use of pain medications in labor, delayed discharge from hospital, and lactation. No identifiers will be collected for the infants (such as birthdate).  **Main outcomes** (Pittsburgh Sleep Quality Index and Edinburgh Postnatal Depression Scale)  **Intervention sustainability** (continued use of the intervention, satisfaction, and feedback) | | | | | | | | | | | | | | | | | | | | | | | | | | | | | | | | | | | | | | | | | | | | | | | |
| **5.3 Data sources.** For all types of data that you will access or collect for this research: Identify whether you are obtaining the data from the subjects (or subjects’ specimens) or whether you are obtaining the data from some other source (and identify the source). | | | | | | | | | | | | | | | | | | | | | | | | | | | | | | | | | | | | | | | | | | | | | | | | | | |
| *If you have already provided this information in Question 5.1, you do not need to repeat the information here.* | | | | | | | | | | | | | | | | | | | | | | | | | | | | | | | | | | | | | | | | | | | | | | | | | | |
|  | | | Direct measurement: actigraphy  Self-report: all surveys/questionnaires  REDCap OPTIMISM Website use – site tracks unique ID logon date, time, duration, and module access | | | | | | | | | | | | | | | | | | | | | | | | | | | | | | | | | | | | | | | | | | | | | | | |
| **5.4 Retrospective/prospective.** For all types of data and specimens that you will access or collect for this research: do all data and specimens to be used in the research exist (for example, in subjects’ medical records) at the time this application is being submitted for initial review? | | | | | | | | | | | | | | | | | | | | | | | | | | | | | | | | | | | | | | | | | | | | | | | | | | |
|  | | | **x** | | | | | | **No** | | | | | | |  | | | | | | | | | | | | | | | | | | | | | | | | | | | | | | | | | | |
|  | | |  | | | | | | **Yes** | | | | | | |  | | | | | | | | | | | | | | | | | | | | | | | | | | | | | | | | | | |
| Include any necessary comments or explanation below (Note that for most studies this can be left blank): | | | | | | | | | | | | | | | | | | | | | | | | | | | | | | | | | | | | | | | | | | | | | | | | | | |
|  | | | |  | | | | | | | | | | | | | | | | | | | | | | | | | | | | | | | | | | | | | | | | | | | | | | |
| **5.5** **Identifiability of data and specimens**. Answer these questions carefully and completely. This will allow HSD to accurately determine the type of review that is required and to assist you in identifying relevant compliance requirements. Review the following definitions before answering the questions: | | | | | | | | | | | | | | | | | | | | | | | | | | | | | | | | | | | | | | | | | | | | | | | | | | |
| ***Access*** *means to view or perceive data, but not to possess or record it. See, in contrast, the definition of “obtain”.*  ***Identifiable*** *means that the identity of an individual is or may be readily (1) ascertained by the researcher or any other member of the study team from specific data variables or from a combination of data variables, or (2) associated with the information.*  ***Direct identifiers*** *are direct links between a subject and data/specimens. Examples include (but are not limited to): name, date of birth, medical record number, email or IP address, pathology or surgery accession number, student number, or a collection of your data that is (when taken together) identifiable.*  ***Indirect identifiers*** *are information that links between direct identifiers and data/specimens. Examples: a subject code or pseudonym.*  ***Key*** *refers to a single place where direct identifiers and indirect identifiers are linked together so that, for example, coded data can be identified as relating to a specific person. Example: a master list that contains the data code and the identifiers linked to the codes.*  ***Obtain*** *means to possess or record in any fashion (writing, electronic document, video, email, voice recording, etc.) for research purposes and to retain for any length of time. This is different from* ***accessing****, which means to view or perceive data.* | | | | | | | | | | | | | | | | | | | | | | | | | | | | | | | | | | | | | | | | | | | | | | | | | | |
| **a.** Will you or any members of your team have access to any direct or indirect identifiers? | | | | | | | | | | | | | | | | | | | | | | | | | | | | | | | | | | | | | | | | | | | | | | | | | | |
|  | | | | | | | | | | | | **x** | | | | | | **Yes** | | | | | | | | | | 🡪 If yes, describe which identifiers and for which data/specimens. | | | | | | | | | | | | | | | | | | | | | | |
|  | | | | | | | | | | | |  | | | | |  | | | | | | | | | | | | | | | | | | | We will have access to name and contact information for participants. | | | | | | | | | | | | | | |
|  | | | | | | | | | | | |  | | | | **No** | | | | | | | | | | | | 🡪 If no, select the reason(s) why you (and all members of your team) will not have access to direct or indirect identifiers. | | | | | | | | | | | | | | | | | | | | | | |
|  | | | | | | | | | | | |  | | | |  | | | | | | | | | | | |  | | | | | | | | | | | | | | | | | | | | | | |
|  | | | | | | | | | | | | | | | | | | | | | | | | | | | | | | | | | | |  | | | | | | | | There will be no identifiers. | | | | | | | |
|  | | | | | | | | | | | | | | | | | | | | | | | | | | | | | | | | | | |  | | | | | | | |  | | | | | | | |
|  | | | | | | | | | | | | | | | | | | | | | | | | | | | | | | | | | | |  | | | | | | | |  | | | | | | | |
|  | | | | | | | | | | | | | | | | | | | | | | | | | | | | | | | | | | |  | | | | | | | | Identifiers or the key have been (or will have been) destroyed before you have access. | | | | | | | |
|  | | | | | | | | | | | | | | | | | | | | | | | | | | | | | | | | | | |  | | | | | | | |  | | | | | | | |
|  | | | | | | | | | | | | | | | | | | | | | | | | | | | | | | | | | | |  | | | | | | | |  | | | | | | | |
|  | | | | | | | | | | | | | | | | | | | | | | | | | | | | | | | | | | |  | | | | | | | | You have (or will have) entered into an agreement with the holder of the identifiers (or key) that prohibits the release of the identifiers (or key) to you under any circumstances. | | | | | | | |
|  | | | | | | | | | | | | | | | | | | | | | | | | | | | | | | | | | | |  | | | | | | | |  | | | | | | | |
|  | | | | | | | | | | | | | | | | | | | | | | | | | | | | | | | | | | |  | | | | | | | |  | | | | | | | |
|  | | | | | | | | | | | | | | | | | | | | | | | | | | | | | | | | | | |  | | | | | | | | *You should be able to produce this agreement for IRB upon request. Examples: a Data Use Agreement, Repository Gatekeeping form, or documented email.* | | | | | | | |
|  | | | | | | | | | | | | | | | | | | | | | | | | | | | | | | | | | | |  | | | | | | | | There are written policies and procedures for the repository/database/data management center that prohibit the release of the identifiers (or identifying link). This includes situations involving an Honest Broker. | | | | | | | |
|  | | | | | | | | | | | | | | | | | | | | | | | | | | | | | | | | | | |  | | | | | | | |  | | | | | | | |
|  | | | | | | | | | | | | | | | | | | | | | | | | | | | | | | | | | | |  | | | | | | | |  | | | | | | | |
|  | | | | | | | | | | | | | | | | | | | | | | | | | | | | | | | | | | |  | | | | | | | | There are other legal requirements prohibiting the release of the identifiers or key to you. Describe them below. | | | | | | | |
|  | | | | | | | | | | | | | | | | | | | | | | | | | | | | | | | | | | |  | | | | | | | |  | | | | | | | |
|  | | | | | | | | | | | | | | | | | | | | | | | | | | | | | | | | | | |  | | | | | | | |  | | | | | | | |
|  | | | | | | | | | | | | | | | | | | | | | | | | | | | | | | | | | | |  | | | | | | | |  | | | | | | | |
| **b.** Will you obtain any direct or indirect identifiers? | | | | | | | | | | | | | | | | | | | | | | | | | | | | | | | | | | | | | | | | | | | | | | | | | | |
|  | | | | | | | | | | | | **x** | | | | | **Yes** | | | | | | | | | | | 🡪 If yes, describe which identifiers and for which data/specimens. | | | | | | | | | | | | | | | | | | | | | | |
|  | | | | | | | | | | | |  | | | |  | | | | | | | | | | | |  | | | | | | | | | Legal first, middle (if applicable), last name; date of birth; city/country of birth; gender at birth; email address; telephone number; home address; for study communication, study mailings  OPTIMISM website will track IP address for website login dates, usage | | | | | | | | | | | | | |
|  | | | | | | | | | | | |  | | | | **No** | | | | | | | | | | | | 🡪 If no, select the reason(s) why you (and all members of your team) will not obtain direct or indirect identifiers. | | | | | | | | | | | | | | | | | | | | | | |
|  | | | | | | | | | | | |  | | | |  | | | | | | | | | | | |  | | | | | | | | | | | | | | | | | | | | | | |
|  | | | | | | | | | | | | | | | | | | | | | | | | | | | | | | | | | | |  | | | | | | | | There will be no identifiers. | | | | | | | |
|  | | | | | | | | | | | | | | | | | | | | | | | | | | | | | | | | | | |  | | | | | | | |  | | | | | | | |
|  | | | | | | | | | | | | | | | | | | | | | | | | | | | | | | | | | | |  | | | | | | | |  | | | | | | | |
|  | | | | | | | | | | | | | | | | | | | | | | | | | | | | | | | | | | |  | | | | | | | | Identifiers or the key have been (or will have been) destroyed before you have access. | | | | | | | |
|  | | | | | | | | | | | | | | | | | | | | | | | | | | | | | | | | | | |  | | | | | | | |  | | | | | | | |
|  | | | | | | | | | | | | | | | | | | | | | | | | | | | | | | | | | | |  | | | | | | | |  | | | | | | | |
|  | | | | | | | | | | | | | | | | | | | | | | | | | | | | | | | | | | |  | | | | | | | | You have (or will have) entered into an agreement with the holder of the identifiers (or key) that prohibits the release of the identifiers (or key) to you under any circumstances. | | | | | | | |
|  | | | | | | | | | | | | | | | | | | | | | | | | | | | | | | | | | | |  | | | | | | | |  | | | | | | | |
|  | | | | | | | | | | | | | | | | | | | | | | | | | | | | | | | | | | |  | | | | | | | |  | | | | | | | |
|  | | | | | | | | | | | | | | | | | | | | | | | | | | | | | | | | | | |  | | | | | | | | *You should be able to produce this agreement for IRB upon request. Examples: a Data Use Agreement, Repository Gatekeeping form, or documented email.* | | | | | | | |
|  | | | | | | | | | | | | | | | | | | | | | | | | | | | | | | | | | | |  | | | | | | | | There are written policies and procedures for the repository/database/data management center that prohibit the release of the identifiers (or identifying link). This includes situations involving an Honest Broker. | | | | | | | |
|  | | | | | | | | | | | | | | | | | | | | | | | | | | | | | | | | | | |  | | | | | | | |  | | | | | | | |
|  | | | | | | | | | | | | | | | | | | | | | | | | | | | | | | | | | | |  | | | | | | | |  | | | | | | | |
|  | | | | | | | | | | | | | | | | | | | | | | | | | | | | | | | | | | |  | | | | | | | | There are other legal requirements prohibiting the release of the identifiers or key to you. Describe them below. | | | | | | | |
|  | | | | | | | | | | | | | | | | | | | | | | | | | | | | | | | | | | |  | | | | | | | |  | | | | | | | |
|  | | | | | | | | | | | | | | | | | | | | | | | | | | | | | | | | | | |  | | | | | | | |  | | | | | | | |
|  | | | | | | | | | | | | | | | | | | | | | | | | | | | | | | | | | | |  | | | | | | | | | |  | | | | | |
| **c.** If you obtain any identifiers, indicate how the identifiers will be stored (and for which data). NOTE: Do not describe your data security plan here – we will ask for that information in section 9.6. | | | | | | | | | | | | | | | | | | | | | | | | | | | | | | | | | | | | | | | | | | | | | | | | | | |
|  | | | | | | | | | | | | | | | | | | | | | | | | | | | | | | | | | | |  | | | | | | | | You will store the identifiers with the data. Describe the data to which this applies: | | | | | | | |
|  | | | | | | | | | | | | | | | | | | | | | | | | | | | | | | | | | | |  | | | | | | | |  | | | | | | | |
|  | | | | | | | | | | | | | | | | | | | | | | | | | | | | | | | | | | |  | | | | | | | |  | | | | | | | |
|  | | | | | | | | | | | | | | | | | | | | | | | | | | | | | | | | | | |  | | | | | | | |  | | | | | | | |
|  | | | | | | | | | | | | | | | | | | | | | | | | | | | | | | | | | | |  | | | | | | | | You will store identifiers and study data separately but you will maintain a link between the identifiers and the study data (for example, through the use of a code). Describe the data to which this applies: | | | | | | | |
|  | | | | | | | | | | | | | | | | | | | | | | | | | | | | | | | | | | | **x** | | | | | | | |  | | | | | | | |
|  | | | | | | | | | | | | | | | | | | | | | | | | | | | | | | | | | | |  | | | | | | | |  | | | | | | | |
|  | | | | | | | | | | | | | | | | | | | | | | | | | | | | | | | | | | |  | | | | | | | | All participant raw data will be coded and stored separately from identifying information on a password protected server to which only study staff have access. Online data will be saved in a secure password protected REDCap database that is protected by the highest standards for electronic data safety. The data stored on the central server will be automatically coded with participant ID and no personal identifiers will be associated with these data. | | | | | | | |
|  | | | | | | | | | | | | | | | | | | | | | | | | | | | | | | | | | | |  | | | | | | | | You will store identifiers separately from the study data, with no link between the identifiers and the study data. Describe the data to which this applies: | | | | | | | |
|  | | | | | | | | | | | | | | | | | | | | | | | | | | | | | | | | | | |  | | | | | | | |  | | | | | | | |
|  | | | | | | | | | | | | | | | | | | | | | | | | | | | | | | | | | | |  | | | | | | | |  | | | | | | | |
|  | | | | | | | | | | | | | | | | | | | | | | | | | | | | | | | | | | |  | | | | | | | |  | | | | | | | |
| **d.** **Research collaboration**. Will individuals who provide you with coded information or specimens for your research also collaborate on other activities for this research? If yes, identify the activities and provide the name of the collaborator’s institution/organization. | | | | | | | | | | | | | | | | | | | | | | | | | | | | | | | | | | | | | | | | | | | | | | | | | | |
| *Examples include but are not limited to: (1) study, interpretation, or analysis of the data that results from the coded information or specimens; and (2) authorship on presentations or manuscripts related to this work.* | | | | | | | | | | | | | | | | | | | | | | | | | | | | | | | | | | | | | | | | | | | | | | | | | | |
|  | | | | | | | | | | | n/a | | | | | | | | | | | | | | | | | | | | | | | | | | | | | | | | | | | | | | | |
| **5.6 Newborn dried blood spots.** Will you use newborn dried bloodspots collected in the United States on or after March 18, 2015? | | | | | | | | | | | | | | | | | | | | | | | | | | | | | | | | | | | | | | | | | | | | | | | | | | |
|  | | **x** | | | | | | | | **No** | | | | |  | | | | | | | | | | | | | | | | | | | | | | | | | | | | | | | | | | | |
|  | |  | | | | | | | | **Yes** | | | | | 🡪 If yes, is this research supported by any federal funding (including any fellowship or career development award that provides salary support)? | | | | | | | | | | | | | | | | | | | | | | | | | | | | | | | | | | | |
|  | |  | | | | | | | |  | | | | |  | | | | | | | | | | | | | | | | | | | | | | | | | | | | | | | | | | | |
|  | | | | | | | | | | | | | | | | | | | | | |  | | | | | | | | | **No** | | | | | | | | | | |  | | | | | | | | |
|  | | | | | | | | | | | | | | | | | | | | | |  | | | | | | | | | **Yes** | | | | | | | | | | | 🡪 If yes, describe how you will ensure that the bloodspots were collected with parental permission (in compliance with a 2015 law that applies to federal-funded research). | | | | | | | | |
|  | | | | | | | | | | | | | | | | | | | | | |  | | | | | | | | | | | | | | | | | | | |  | | | | | | | | |
|  | | | | | | | | | | | | | | | | | | | | | | | | | | | | | | | | | | | | | | | | | | | | | |  | | | | |
| **5.7 Protected Health Information (PHI).** Will you access, obtain, use, or disclose a participant’s identifiable PHI for any reason (for example, to identify or screen potential subjects, to obtain study data or specimens, for study follow-up) that does not involve the creation or obtaining of a Limited Data Set? | | | | | | | | | | | | | | | | | | | | | | | | | | | | | | | | | | | | | | | | | | | | | | | | | | |
| *PHI is individually-identifiable healthcare record information or clinical specimens from an organization considered a “covered entity” by federal HIPAA regulations, in any form or media, whether electronic, paper, or oral.* ***If you will use UW Medical Records, you must answer yes to this question.*** | | | | | | | | | | | | | | | | | | | | | | | | | | | | | | | | | | | | | | | | | | | | | | | | | | |
|  |  | | | | | | | | | **No** | | | | | | 🡪 If no, skip the rest of this question; go to [question](#q5poin7) **5.8** | | | | | | | | | | | | | | | | | | | | | | | | | | | | | | | | | | |
|  | **x** | | | | | | | | | **Yes** | | | | | | 🡪 If yes, answer all of the questions below. | | | | | | | | | | | | | | | | | | | | | | | | | | | | | | | | | | |
|  |  | | | | | | | | | | | | | | | **a.** Describe the PHI you will access or obtain, and the reason for obtaining it. *Be specific.* | | | | | | | | | | | | | | | | | | | | | | | | | | | | | | | | | | |
|  | | | | | | | | | | | | | | | | | | | | | | | | | We will screen the UW Medicine electronic health record database for potentially eligible participants who meet criteria of pregnancy < 28 weeks gestation and at least 18 years old. This screening will provide the name and email address for these potential participants which will be loaded into REDCap for an automated email invitation to participate in the research study. | | | | | | | | | | | | | | | | | | | | | | | | | |
| **b.** Is any of the PHI located in Washington State? | | | | | | | | | | | | | | | | | | | | | | | | | | | | | | | | | | | | | | | | | | | | | | | | | | |
|  | | | | | | | | | | | | | | | | | | | | | | | | |  | | | | | | | | | **No** | | | | | | | | | |  | | | | | | |
|  | | | | | | | | | | | | | | | | | | | | | | | | | **x** | | | | | | | | | **Yes** | | | | | | | | | |  | | | | | | |
| **c.** Describe how you will access or obtain the PHI. *Be specific.* | | | | | | | | | | | | | | | | | | | | | | | | | | | | | | | | | | | | | | | | | | | | | | | | | | |
|  | | | | | | | | | | | | | | | | | | | | | | | | | We will be use the Biomedical Informatics (BMI) services group affiliated with the ITHS and UW Medicine IT to identify our target population (pregnant women at least 18 years old who are less than 28 weeks pregnant) from within the UW Medicine medical records. They will provide us with basic contact information plus current pregnancy status. They will load that information into a dedicated REDCap database and email invitations will be automatically sent via the REDCap survey mechanism. | | | | | | | | | | | | | | | | | | | | | | | | | |
| **d.** For which PHI will you obtain HIPAA authorization from the subjects by having them sign a HIPAA Authorization form, before obtaining and using the PHI? | | | | | | | | | | | | | | | | | | | | | | | | | | | | | | | | | | | | | | | | | | | | | | | | | | |
|  | | | | | | | | | | | | | | | | | | | | | | | | | | None | | | | | | | | | | | | | | | | | | | | | | | | |
|  | | | | | | | | | | | | | | | | | | | | | | | | | | Confirm by checking the box that you will use the UW Medicine [HIPAA Authorization](https://www.washington.edu/research/forms-and-templates/template-hipaa-authorization/) form maintained on the HSD website if you will access, obtain, use, or disclose UW Medicine PHI. | | | | | | | | | | | | | | | | | | | | | | | | |
|  | | | | | | | | | | | | | | | | | | | | | | | | | |  | | | | | | | **Confirmed** | | | | | | | | | | | | | | | | |  |
| **e**. For which PHI will you NOT obtain HIPAA authorization from the subjects? | | | | | | | | | | | | | | | | | | | | | | | | | | | | | | | | | | | | | | | | | | | | | | | | | | |
|  | | | | | | | | | | | | | | | | | | | | | | | | | | name, email address | | | | | | | | | | | | | | | | | | | | | | | | |
|  | | | | | | | | | | | | | | | | | | | | | | | | | | Provide the following assurances by checking the boxes. | | | | | | | | | | | | | | | | | | | | | | | | |
|  | | | | | | | | | | | | | | | | | | | | | | | | | |  | | | | | | The PHI will not be reused or disclosed to any other person or entity, except as required by law, for authorized oversight of the research study, or for other research for which the use or disclosure of PHI would be permitted. | | | | | | | | | | | | | | | | | | |
|  | | | | | | | | | | | | | | | | | | | | | | | | | | **x** | | | | | |  | | | | | | | | | | | | | | | | | | |
|  | | | | | | | | | | | | | | | | | | | | | | | | | |  | | | | | |  | | | | | | | | | | | | | | | | | | |
|  | | | | | | | | | | | | | | | | | | | | | | | | | |  | | | | | | You will fulfill the HIPAA “accounting for disclosures” requirement. See [UW Medicine Privacy Policy #25](http://depts.washington.edu/comply/docs/PP_25.pdf). THIS IS ONLY FOR UW RECORDS. | | | | | | | | | | | | | | | | | | |
|  | | | | | | | | | | | | | | | | | | | | | | | | | | **x** | | | | | |  | | | | | | | | | | | | | | | | | | |
|  | | | | | | | | | | | | | | | | | | | | | | | | | |  | | | | | |  | | | | | | | | | | | | | | | | | | |
|  |  | | | | | | | | | | | |  | | | | | | | | | | | | |  | | | | | | There will be reasonable safeguards to protect against identifying, directly or indirectly, any patient in any report of the research. | | | | | | | | | | | | | | | | | | |
|  |  | | | | | | | | | | | |  | | | | | | | | | | | | | **x** | | | | | |  | | | | | | | | | | | | | | | | | | |
|  |  | | | | | | | | | | | |  | | | | | | | | | | | | |  | | | | | |  | | | | | | | | | | | | | | | | | | |
| **5.8 Genomic data sharing**. Will you obtain or generate genomic data (as defined at  <http://osp.od.nih.gov/scientific-sharing/genomic-data-sharing-faqs/> )? | | | | | | | | | | | | | | | | | | | | | | | | | | | | | | | | | | | | | | | | | | | | | | | | | | |
|  | **x** | | | | | | | **No** | | | | | | | |  | | | | | | | | | | | | | | | | | | | | | | | | | | | | | | | | | | |
|  |  | | | | | | | **Yes** | | | | | | | | 🡪 If yes, answer the question below. | | | | | | | | | | | | | | | | | | | | | | | | | | | | | | | | | | |
|  | | | | | | | | | | | | | | | | | | | | | **a.** Do you plan to send genomic data from this research to a national database (for example, NIH’s dbGaP database)? | | | | | | | | | | | | | | | | | | | | | | | | | | | | | |
|  | | | | | | | | | | | | | | | | | | | | | | | | | | | | |  | | | | | | | | | | | **No** | | | | | | |  | | | |
|  | | | | | | | | | | | | | | | | | | | | | | | | | | | | |  | | | | | | | | | | | **Yes** | | | | | | | 🡪 If yes, complete the [***ZIPLINE*** **SUPPLEMENT Genomic Data Sharing**](https://www.washington.edu/research/forms-and-templates/zipline-supplement-genomic-data-sharing/) and upload it to the **Supporting Documents** SmartForm of ***Zipline***. | | | |
|  | | | | | | | | | | | | | | | | | | | | | | | | | | | | |  | | | | | | | | | | | | | | | | | |  | | | |
| **5.9 Whole genome sequencing.** For research involving biospecimens: Will the research include whole genome sequencing? | | | | | | | | | | | | | | | | | | | | | | | | | | | | | | | | | | | | | | | | | | | | | | | | | | |
| *Whole genome sequencing is sequencing of a human germline or somatic specimen with the intent to generate the genome or exome sequence of that specimen.* | | | | | | | | | | | | | | | | | | | | | | | | | | | | | | | | | | | | | | | | | | | | | | | | | | |
|  | **x** | | | | | **No** | | | | | | |  | | | | | | | | | | | | | | | | | | | | | | | | | | | | | | | | | | | | | |
|  |  | | | | | **Yes** | | | | | | |  | | | | | | | | | | | | | | | | | | | | | | | | | | | | | | | | | | | | | |
| **5.10 Data and specimen sharing/banking**. Are you likely to share some or all of the data, specimens, or subject contact information with other researchers or a repository/database for research purposes not related to this study, or to bank them for your own future unspecified research uses? **You are strongly encouraged to consider the broadest possible future plans you might have, and whether you will obtain consent now from the subjects for future sharing or unspecified uses.** Answer **YES** even if you will only share information without identifiers. Answer **NO** if you are unlikely to do any sharing, or if your only sharing will be through the NIH Genomic Data Sharing described in [question 5.8](#q5poin7). | | | | | | | | | | | | | | | | | | | | | | | | | | | | | | | | | | | | | | | | | | | | | | | | | | |
| *Many federal grants and contracts now require data or specimen sharing as a condition of funding, and many journals require data sharing as a condition of publication. “Sharing” may include: informal arrangements to share your banked data/specimens with other investigators; establishing a repository from which you formally share with others through written agreements; or sending your data/specimens to a third party repository/archive/entity such as the Social Science Open Access Repository (SSOAR), or the UCLA Ethnomusicology Archive.* | | | | | | | | | | | | | | | | | | | | | | | | | | | | | | | | | | | | | | | | | | | | | | | | | | |
|  |  | | | | | **No** | | | | | | |  | | | | | | | | | | | | | | | | | | | | | | | | | | | | | | | | | | | | | |
|  | **x** | | | | | **Yes** | | | | | | | 🡪 If yes, answer all of the questions below. | | | | | | | | | | | | | | | | | | | | | | | | | | | | | | | | | | | | | |
|  | | | | | | | | | | | | | | | | | | | | **a.** Describe what will be stored, including whether any direct or indirect (e.g., subject codes) identifiers will be stored. | | | | | | | | | | | | | | | | | | | | | | | | | | | | | | |
|  | | | | | | | | | | | | | | | | | | | | | | | | | | | | Funding requires use of NIH common data elements Biomedical Research Informatics Computing System (BRICS). This system uses a computer program that researchers download onto their university computer. Researchers then enter personally identifiable subject information to derive a series of one-way hashes which securely encrypt the subject information. One way hashes are sent to the GUID server for reference and storage. No personally identifiable information is sent to the system. The GUID server returns a GUID identifier. If the one way hashes match a known subject, an existing GUID will be returned; if it is a new subject, a new GUID will be returned. PI will store GUIDs for subjects. | | | | | | | | | | | | | | | | | | | | | | |
|  | | | | | | | | | | | | | | | | | | | | **b.** Describe what will be shared, including whether direct identifiers will be shared and (for specimens) what data will be released with the specimens. | | | | | | | | | | | | | | | | | | | | | | | | | | | | | | |
|  | | | | | | | | | | | | | | | | | | | | | | | | | | | Once the researcher has a GUID, s/he may submit data associated with this subject. Researchers are then able to access data in the BRICS database across studies without revealing personally identifiable information. Use of GUID is required by the NIH/NINR funding mechanism for this study.  Common Data Elements shared will not contain PHI, but rather the GUID, and relate to PROMIS measures (sleep disturbance, global health, fatigue, anxiety, depression scores). | | | | | | | | | | | | | | | | | | | | | | | |
|  | | | | | | | | | | | | | | | | | | | | **c.** Who will oversee and/or manage the sharing? | | | | | | | | | | | | | | | | | | | | | | | | | | | | | | |
|  | | | | | | | | | | | | | | | | | | | | | | | | | | | Principal Investigator | | | | | | | | | | | | | | | | | | | | | | | |
|  | | | | | | | | | | | | | | | | | | | **d.** Describe the possible future uses, including limitations or restrictions (if any) on future uses or users. As stated above, consider the broadest possible uses. | | | | | | | | | | | | | | | | | | | | | | | | | | | | | | | |
|  | | | | | | | | | | | | | | | | | | | | | | | | | | | *Examples: data will be used only for cardiovascular research; data will not be used for research on population origins.* | | | | | | | | | | | | | | | | | | | | | | | |
|  | | | | | | | | | | | | | | | | | | | | | | | | | | | Common data elements will be accessible by researchers to potentially pool and analyze data across studies. | | | | | | | | | | | | | | | | | | | | | | | |
|  | | | | | | | | | | | | | | | | | | | | **e.** Consent. Will you obtain consent now from subjects for the banking and/or future sharing? | | | | | | | | | | | | | | | | | | | | | | | | | | | | | | |
|  | | | | | | | | | | | | | | | | | | | | | | | | | | | | |  | | | | | | | | | | | | **No** | | | | | |  | | | |
|  | | | | | | | | | | | | | | | | | | | | | | | | | | | | | **x** | | | | | | | | | | | | **Yes** | | | | | | 🡪 If yes, be sure to include the information about this consent process in the consent form (if there is one) and in your answers to the consent questions in [Section 8](#section8). | | | |
|  | | | | | | | | | | | | | | | | | | | | | | | | | | | | |  | | | | | | | | | | | | | | | | | |  | | | |
|  | | | | | | | | | | | | | | | | | | | | | | | **f.** Withdrawal. Will subjects be able to withdraw their data/specimens from banking or sharing? | | | | | | | | | | | | | | | | | | | | | | | | | | | |
|  | | | | | | | | | | | | | | | | | | | | | | | | | | | | | **x** | | | | | | | | | | **No** | | | | | | | |  | | | |
|  | | | | | | | | | | | | | | | | | | | | | | | | | | | | |  | | | | | | | | | | **Yes** | | | | | | | | 🡪 If yes, describe how, and whether there are any limitations on withdrawal. | | | |
|  | | | | | | | | | | | | | | | | | | | | | | | | | | | | | | | | | | | | | | | | | | | | | | | | *Example: data can be withdrawn from the repository but cannot be retrieved after they are released.* | | |
|  | | | | | | | | | | | | | | | | | | | | | | | | | | | | | | | | | | | | | | | | | | | | | | | |  | | |
|  | | | | | | | | | | | | | | | | | | | | | | | | **g.** Agreements for sharing or release. Confirm by checking the box that you will comply with UW (and, if applicable, UW Medicine) policies that require a formal agreement between you and the recipient for release of data or specimens to individuals or entities other than federal databases. | | | | | | | | | | | | | | | | | | | | | | | | | | |
|  | | | | | | | | | | | | | | | | | | | | | | | | | | | | | | *Data Use Agreements or Gatekeeping forms are used for data; Material Transfer Agreements are used for specimens (or specimens plus data. Do not attach your template agreement forms; the IRB neither reviews nor approves them* | | | | | | | | | | | | | | | | | | | | |
|  | | | | | | | | | | | | | | | | | | | | | | | | | | | | | **x** | | | | | | | | | **Confirmed** | | | | | | | | | | |  | |
| **5.11 Communication with subjects during the study**. Describe the types of communication (if any) you will have with already-enrolled subjects during the study. Provide a description instead of the actual materials themselves. | | | | | | | | | | | | | | | | | | | | | | | | | | | | | | | | | | | | | | | | | | | | | | | | | | |
| *Examples: email, texts, phone, or letter reminders about appointments or about returning study materials such as a questionnaire; requests to confirm contact information.* | | | | | | | | | | | | | | | | | | | | | | | | | | | | | | | | | | | | | | | | | | | | | | | | | | |
|  | | | Email will be used to provide links to the study assessments, reminders on participation in the intervention, and weekly progress on sleep goals. An online discussion board will provide participants with opportunities to connect with other participants and the mindfulness instructor and to ask/answer questions. Short messages or reminders about content or participation will also be sent by text message. Research staff may telephone participants if requested to help with completion of assessment or access to study materials. The PI may contact participants by telephone if there are potential concerns about participant wellbeing. | | | | | | | | | | | | | | | | | | | | | | | | | | | | | | | | | | | | | | | | | | | | | | | |
| **5.12** **Future contact with subjects**. Do you plan to retain any contact information you obtain for your subjects so that they can be contacted in the future? | | | | | | | | | | | | | | | | | | | | | | | | | | | | | | | | | | | | | | | | | | | | | | | | | | |
|  | **x** | | | | | | | **No** | | | | | | | |  | | | | | | | | | | | | | | | | | | | | | | | | | | | | | | | | | | |
|  |  | | | | | | | **Yes** | | | | | | | | 🡪 If yes, describe the purpose of the future contact, and whether use of the contact information will be limited to your team; if not, describe who else could be provided with the contact information. Describe your criteria for approving requests for the information. | | | | | | | | | | | | | | | | | | | | | | | | | | | | | | | | | | |
|  |  | | | | | | | | | | | | | | |  | | | | | | | | | | | | | | | | | | | | | | | | | | | | | | | | | | |
|  | | | | | | | | | | | | | | | | | | | | *Examples: inform subjects about other studies; ask subjects for additional information or medical record access that is not currently part of the study proposed in this application; obtain another sample.* | | | | | | | | | | | | | | | | | | | | | | | | | | | | | | |
|  | | | | | | | | | | | | | | | | | | | |  | | | | | | | | | | | | | | | | | | | | | | | | | | | | | | |
| **5.13** **Alternatives to participation.** Are there any alternative procedures or treatments that might be advantageous to the subjects? | | | | | | | | | | | | | | | | | | | | | | | | | | | | | | | | | | | | | | | | | | | | | | | | | | |
| *If there are no alternative procedures or treatments, select “No”. Examples of advantageous alternatives: earning extra class credit in some time-equivalent way other than research participation; obtaining supportive care or a standard clinical treatment from a health care provider instead of participating in research with an experimental drug.* | | | | | | | | | | | | | | | | | | | | | | | | | | | | | | | | | | | | | | | | | | | | | | | | | | |
|  |  | | | | | | **No** | | | | | | |  | | | | | | | | | | | | | | | | | | | | | | | | | | | | | | | | | | | | |
|  | **x** | | | | | | **Yes** | | | | | | | 🡪 If yes, describe the alternatives. | | | | | | | | | | | | | | | | | | | | | | | | | | | | | | | | | | | | |
|  | |  | | | | | |  | | | | | | | | | | | | Subjects may seek care sleep difficulty from a primary care provider, prenatal care provider, psychiatric or mental health provider, or sleep specialist. | | | | | | | | | | | | | | | | | | | | | | | | | | | | | | |
| **5.14** **Upload to the Supporting Documents** SmartForm of ***Zipline*** all data collection forms (if any) that will be directly used by or with the subjects, and any scripts/talking points you will use to collect the data. Do not include data collection forms that will be used to abstract data from other sources (such as medical or academic records, or video recordings. | | | | | | | | | | | | | | | | | | | | | | | | | | | | | | | | | | | | | | | | | | | | | | | | | | |
| - ***Examples****: survey, questionnaires, subject logs or diaries, focus group questions.* - ***NOTE:*** *Sometimes the IRB can approve the general content of surveys and other data collection instruments rather than the specific form itself. This prevents the need to submit a modification request for future minor changes that do not add new topics or increase the sensitivity of the questions. To request this general approval, use the text box below to identify the questionnaires/surveys/ etc. for which you are seeking this more general approval. Then briefly describe the scope of the topics you will cover and the most personal and sensitive questions. The HSD staff person who screens this application will let you know whether this is sufficient or whether you will need to provide more information.* - ***For materials that cannot be uploaded****: upload screenshots or written descriptions that are sufficient to enable the IRB to understand the types of data that will be collected and the nature of the experience for the participant. You may also provide URLs (website addresses) or written descriptions below. Examples of materials that usually cannot be uploaded: mobile apps; computer-administered test; licensed and restricted standardized tests.* - ***For data that will be gathered in an evolving way****: This refers to data collection/questions that are not pre-determined but rather are shaped during interactions with participants in response to observations and responses made during those interactions. If this applies to your research, provide a description of the process by which you will establish the data collection/questions as you interact with subjects, how you will document your data collection/questions, the topics you plan to address, the most sensitive type of information you will plan to gather, and the limitations (if any) on topics you will raise or pursue.* | | | | | | | | | | | | | | | | | | | | | | | | | | | | | | | | | | | | | | | | | | | | | | | | | | |
| Use this text box (if desired) to provide:   - Short written descriptions of materials that cannot be uploaded, such as URLs - A description of the process you will use for data that will be gathered in an evolving way. - The general content of questionnaires, surveys and similar instruments for which you are seeking general approval. (See the **NOTE** bullet point in the instructions above.) | | | | | | | | | | | | | | | | | | | | | | | | | | | | | | | | | | | | | | | | | | | | | | | | | | |
|  | | | | | Primary data collection method will be via REDCap surveys – PDF versions of these surveys will be attached. | | | | | | | | | | | | | | | | | | | | | | | | | | | | | | | | | | | | | | | | | | | | | |
| **5.15** **Send HSD a** [**Confidentiality Agreement**](https://www.washington.edu/research/forms-and-templates/zipline-and-paper-template-confidentiality-agreement/) if you will obtain or use any private identifiable UW records without subject’s written consent (for example, screening medical records or class grades to identify possible subjects). | | | | | | | | | | | | | | | | | | | | | | | | | | | | | | | | | | | | | | | | | | | | | | | | | | |
| *The Confidentiality Agreement form must be completed, printed, signed, and mailed to the Human Subjects Division at Box 359470. Your IRB application cannot be approved until we receive the Confidentiality Agreement.* | | | | | | | | | | | | | | | | | | | | | | | | | | | | | | | | | | | | | | | | | | | | | | | | | | |

| **6 CHILDREN (MINORS) and PARENTAL PERMISSION** | | | | | | | | | | | | | | | | | | | | | | |
| --- | --- | --- | --- | --- | --- | --- | --- | --- | --- | --- | --- | --- | --- | --- | --- | --- | --- | --- | --- | --- | --- | --- |
| **6.1 Involvement of minors**. Does your research include minors (children)? | | | | | | | | | | | | | | | | | | | | | | |
| **Minor or child** means someone who has not yet attained the legal age for consent for the research procedures, as described in the applicable laws of the jurisdiction in which the research will be conducted. This may or may not be the same as the definition used by funding agencies such as the National Institutes of Health.   - In Washington State the generic age of consent is 18, meaning that anyone under the age of 18 is considered a child. - There are some procedures for which the age of consent is much lower in Washington State. - The generic age of consent may be different in other states, and in other countries. | | | | | | | | | | | | | | | | | | | | | | |
|  | | **x** | | **No** | | | | | 🡪 If no, go to [Section 8](#Consent). | | | | | | | | | | | | | |
|  | |  | | **Yes** | | | | | 🡪 If yes, provide the age range of the minor subjects for this study and the legal age for consent in your population(s). If there is more than one answer, explain. | | | | | | | | | | | | | |
|  | |  | | | | | | |  | | | | | | | | | | | | | |
|  |  | | | | | | | | | | |  | | | | | | | | | | |
|  | |  | | **Don’t know** | | | | | | | | | 🡪This means is it not possible to know the age of your subjects. For example, this may be true for some research involving social media, the Internet, or a dataset that you obtain from another researcher or from a government agency. Go to [Section 8](#Consent). | | | | | | | | | |
|  | |  | |  | | | | | | | | |  | | | | | | | | | |
|  | |  | |  | | | | | | | | |  | | | | | | | | | |
| **6.2 Parental permission.** **Parental permission** means actively obtaining the permission of the parents. This is not the same as “passive” or “opt out” permission where it is assumed that parents are allowing their children to participate because they have been provided with information about the research and have not objected or returned a form indicating they don’t want their children to participate. | | | | | | | | | | | | | | | | | | | | | | |
| **a.** Will you obtain parental permission for: | | | | | | | | | | | | | | | | | | | | | | |
|  | | |  | | | All of your research procedures | | | | | | | | 🡪 Go to [question **6.2b.**](#q6point2b) | | | | | | | | |
|  | | |  | | |  | | | | | | | |  | | | | | | | | |
|  | | |  | | |  | | | | | | | |  | | | | | | | | |
|  | | |  | | | None of your research procedures | | | | | | | | 🡪 Use the table below to provide your justification, and skip question 6.2b. | | | | | | | | |
|  | | |  | | |  | | | | | | | |  | | | | | | | | |
|  | | |  | | |  | | | | | | | |  | | | | | | | | |
|  | | |  | | | Some of your research procedures | | | | | | | | 🡪 Use the table below to identify the procedures for which you will not obtain written parental permission. | | | | | | | | |
|  | | |  | | |  | | | | | | | |  | | | | | | | | |
|  | | |  | | |  | | | | | | | |  | | | | | | | | |
| *Be sure to consider all research procedures and plans, including screening, future contact, and sharing/banking of data and specimens for future work.* | | | | | | | | | | | | | | | | | | | | | | |
| **Children Group^1^** | | | | | | | | | | **Describe the procedures or data/specimen collection (if any) for which there will be NO parental permission^2^** | | | | | **Reason why you will not obtain parental permission** | **Will you inform them about the research?^3^** | | | | | | |
|  | | | | | | | | | |  | | | | |  | **YES** | | | | **NO** | | |
|  | | | | | | | | | |  | | | | |  |  |  |  |  | |  |  |
|  | | | | | | | | | |  | | | | |  |  |  |  |  | |  |  |
|  | | | | | | | | | |  | | | | |  |  |  |  |  | |  |  |
|  | | | | | | | | | |  | | | | |  |  |  |  |  | |  |  |
|  | | | | | | | | | |  | | | | |  |  |  |  |  | |  |  |
|  | | | | | | | | | |  | | | | |  |  |  |  |  | |  |  |
|  | | | | | | | | | |  | | | | |  |  |  |  |  | |  |  |
|  | | | | | | | | | |  | | | | |  |  |  |  |  | |  |  |
|  | | | | | | | | | |  | | | | |  |  |  |  |  | |  |  |
|  | | | | | | | | | |  | | | | |  |  |  |  |  | |  |  |
|  | | | | | | | | | |  | | | | |  |  |  |  |  | |  |  |
|  | | | | | | | | | |  | | | | |  |  |  |  |  | |  |  |
|  | | | | | | | | | |  | | | | |  |  |  |  |  | |  |  |
|  | | | | | | | | | |  | | | | |  |  |  |  |  | |  |  |
|  | | | | | | | | | |  | | | | |  |  |  |  |  | |  |  |
|  | | | | | | | | | |  | | | | |  |  |  |  |  | |  |  |
|  | | | | | | | | | |  | | | | |  |  |  |  |  | |  |  |
|  | | | | | | | | | |  | | | | |  |  |  |  |  | |  |  |
| *Table footnotes*   1. *If your answer is the same for all children groups or all procedures, you can collapse your answer across the groups and/or procedures.* 2. *If you plan to obtain identifiable information or biospecimens without parent permission, any waiver granted by the IRB does not override parents’ refusal to provide broad consent (for example, through the Northwest Biotrust).* 3. *Will you inform them about the research beforehand even though you are not obtaining active permission?* | | | | | | | | | | | | | | | | | | | | | | |
| **b.** Indicate by checking the appropriate box(es) your plan for obtaining parental permission | | | | | | | | | | | | | | | | | | | | | | |
|  | | |  | | | | Both parents, unless one parent is deceased, unknown, incompetent, or not reasonably available; or when only one parent has legal responsibility for the care and custody of the child | | | | | | | | | | | | | | | |
|  | | |  | | | |  | | | | | | | | | | | | | | | |
|  | | |  | | | |  | | | | | | | | | | | | | | | |
|  | | |  | | | | One parent, even if the other parent is alive, known, competent, reasonably available, and shares legal responsibility for the care and custody of the child. | | | | | | | | | | | | | | | |
|  | | |  | | | |  | | | | | | | | | | | | | | | |
|  | | |  | | | |  | | | | | | | | | | | | | | | |
|  | | |  | | | | *This is all that is required for minimal risk research.* | | | | | | | | | | | | | | | |
|  | | | | | | | | If you checked both boxes, explain: | | | | | | | | | | | | | | |
|  | | | | | | | |  | | | | | | | | | | | | | | |
| **6.3 Children who are wards.** Will any of the children be wards of the State or any other agency, institution, or entity? | | | | | | | | | | | | | | | | | | | | | | |
|  | |  | | | **No** | | | |  | | | | | | | | | | | | | |
|  | |  | | | **Yes** | | | | 🡪 If yes, an advocate may need to be appointed for each child who is a ward. The advocate must be in addition to any other individual acting on behalf of the child as guardian or in loco parentis. The same individual can serve as advocate for all children who are wards. | | | | | | | | | | | | | |
|  | |  | | | | | | |  | | | | | | | | | | | | | |
|  | | | | | | | | | | Describe who will be the advocate(s). Your answer must address the following points:   - Background and experience - Willingness to act in the best interests of the child for the duration of the research - Independence of the research, research team, and any guardian organization | | | | | | | | | | | | |
|  | | | | | | | | | | |  | | | | | | | | | | | |

| **7 ASSENT OF CHILDREN (MINORS)** | | | | | | | | | | | |
| --- | --- | --- | --- | --- | --- | --- | --- | --- | --- | --- | --- |
| *Go to* [*Section 8*](#section8) *if your research does not involve children (minors).* | | | | | | | | | | | |
| **7.1 Assent of children (minors)**. Though children do not have the legal capacity to “consent” to participate in research, they should be involved in the process if they are able to “assent” by having a study explained to them and/or by reading a simple form about the study, and then giving their verbal choice about whether they want to participate. They may also provide a written assent if they are older. See [**WORKSHEET: Children**](https://www.washington.edu/research/forms-and-templates/worksheet-children/) for circumstances in which a child’s assent may be unnecessary or inappropriate. | | | | | | | | | | | |
| **a.** Will you obtain assent for: | | | | | | | | | | | |
|  | | |  | | | | All of your research procedures and child groups | | | | 🡪 Go to [question **7.2**](#q7point2)**.** |
|  | | |  | | | |  |  |  |  |  |
|  | | |  | | | |  |  |  |  |  |
|  | | |  | | | | None of your research procedures and child groups | | | | 🡪 Use the table below to provide your justification, then skip to question 7.5. |
|  | | |  | | | |  |  |  |  |  |
|  | | |  | | | |  |  |  |  |  |
|  | | |  | | | | Some of your research procedures and child groups | | | | 🡪 Use the table below to identify the procedures for which you will not obtain assent. |
|  | | |  | | | |  |  |  |  |  |
|  | | |  | | | |  |  |  |  |  |
| *Be sure to consider all research procedures and plans, including screening, future contact, and sharing/banking of data and specimens for future work.* | | | | | | | | | | | |
| **Children Group^1^** | | | | | | | | **Describe the procedures or data/specimen collection (if any) for which assent will NOT be obtained** | **Reason why you will not obtain assent** | | |
|  | | | | | | | |  |  | | |
|  | | | | | | | |  |  | | |
|  | | | | | | | |  |  | | |
|  | | | | | | | |  |  | | |
|  | | | | | | | |  |  | | |
| *Table footnotes*   1. *If your answer is the same for all children groups or all procedures, you can collapse your answer across the groups and/or procedures.* | | | | | | | | | | | |
| **7.2 Assent process.** Describe how you will obtain assent, for each child group. If your research involves children of different ages, answer separately for each group. If the children are non-English speakers, include a description of how you will ensure that they comprehend the information you provide. | | | | | | | | | | | |
|  |  | | | | | | | | | | |
| **7.3 Dissent or resistance.** Describe how you will identify a child’s objection or resistance to participation (including non-verbal indications) during the research, and what you will do in response. | | | | | | | | | | | |
|  |  | | | | | | | | | | |
| **7.4 Documentation of assent.**  Which of the following statements describes whether you will obtain documentation of assent? | | | | | | | | | | | |
|  |  | | | None of your research procedures and child groups | | | | | | 🡪 Use the table below to provide your justification, then go to question 7.4.a. | |
|  |  | | |  |  |  |  |  |  |  |  |
|  |  | | |  |  |  |  |  |  |  |  |
|  |  | | | All of your research procedures and child groups | | | | | | 🡪 Go to [question **7.4.a**](#q7point4a), do not complete the table | |
|  |  | | |  |  |  |  |  |  |  |  |
|  |  | | |  |  |  |  |  |  |  |  |
|  |  | | | Some of your research procedures and/or child groups | | | | | | 🡪 Complete the table below and then to go question 7.4.a | |
|  |  | | |  |  |  |  |  |  |  |  |
|  |  | | |  |  |  |  |  |  |  |  |
| **Children**  **Group^1^** | | | | | | | | **Describe the procedures or data/specimen collection (if any) for which assent will NOT be documented** | | | |
|  | | | | | | | |  | | | |
|  | | | | | | | |  | | | |
|  | | | | | | | |  | | | |
|  | | | | | | | |  | | | |
|  | | | | | | | |  | | | |
| *Table footnotes*   1. *If your answer is the same for all children groups or all procedures, you can collapse your answer across the groups and/or procedures.* | | | | | | | | | | | |
|  | **a.** **Describe how you will document assent**. If the children are functionally illiterate or are not fluent in English, include a description of what you will do. | | | | | | | | | | |
|  |  | | |  | | | | | | | |
|  | **b.** **Upload all assent materials** (talking points, videos, forms, etc.) to the **Consent Form and Recruitment Materials** SmartForm of ***Zipline***. Assent materials are not required to provide all of the standard elements of adult consent; the information should be appropriate to the age, population, and research procedures. The documents should be in Word, if possible. | | | | | | | | | | |
| **7.5 Children who reach the legal age of consent during participation in longitudinal research.** | | | | | | | | | | | |
| Children who were enrolled at a young age and continue for many years: It is best practice to re-obtain assent (or to obtain it for the first time, if you did not at the beginning of their participation).  Children who reach the legal age of consent: You must obtain informed consent from the now-adult subject for (1) any ongoing interactions or interventions with the subjects, or (2) the continued analysis of specimens or data for which the subject’s identify is readily identifiable to the researcher, unless the IRB waives this requirement. | | | | | | | | | | | |
|  | | **a.** Describe your plans (if any) to re-obtain assent from children. | | | | | | | | | |
|  | | | | |  | | | | | | |
|  | | **b.** Describe your plans (if any) to obtain consent for children who reach the legal age of consent.   - If you plan to obtain consent, describe what you will do about now-adult subjects whom you are unable to contact. - If you do not plan to obtain consent or think that you will be unable to do so, explain why. | | | | | | | | | |
|  | | | | | |  | | | | | |
| **7.6 Other regulatory requirements**. (This is for your information only; no answer or response is required.) Researchers are responsible for determining whether their research conducted in schools, with student records, or over the Internet comply with permission, consent, and inspection requirements of the following federal regulations:   - PPRA – Protection of Pupil Rights Amendment - FERPA – Family Education Rights and Privacy Act - COPPA – Children’s Online Privacy Protection Act | | | | | | | | | | | |

| **8** **CONSENT OF ADULTS** | | | | | | | | | | | | | | | | | | | | | | | | | | | | | | | | | |
| --- | --- | --- | --- | --- | --- | --- | --- | --- | --- | --- | --- | --- | --- | --- | --- | --- | --- | --- | --- | --- | --- | --- | --- | --- | --- | --- | --- | --- | --- | --- | --- | --- | --- |
| **Review the following definitions before answering the questions in this section.** | | | | | | | | | | | | | | | | | | | | | | | | | | | | | | | | | |
| **CONSENT** | | | | | | | | | | | | | | | | | | | | | | | is the process of informing potential subjects about the research and asking them whether they want to participate. It usually (but not always) includes an opportunity for subjects to ask questions. It does not necessarily include the signing of a consent form. This question is about the consent process. | | | | | | | | | | |
| **CONSENT DOCUMENTATION** | | | | | | | | | | | | | | | | | | | | | | | refers to how a subject’s decision to participate in the research is documented. This is typically obtained by having the subject sign a consent form. | | | | | | | | | | |
| **CONSENT FORM** | | | | | | | | | | | | | | | | | | | | | | | is a document signed by subjects, by which they agree to participate in the research as described in the consent form and in the consent process. | | | | | | | | | | |
| **ELEMENTS OF CONSENT** | | | | | | | | | | | | | | | | | | | | | | | are specific information that is required to be provided to subjects. | | | | | | | | | | |
| **PARENTAL PERMISSION** | | | | | | | | | | | | | | | | | | | | | | | is the parent’s active permission for the child to participate in the research. Parental permission is subject to the same requirements as consent, including written documentation of permission and required elements. | | | | | | | | | | |
| **SHORT FORM CONSENT** | | | | | | | | | | | | | | | | | | | | | | | is an alternative way of obtaining written documentation of consent that is most commonly used with individuals who are illiterate or whose language is one for which translated consent forms are not available. | | | | | | | | | | |
| **WAIVER OF CONSENT** | | | | | | | | | | | | | | | | | | | | | | | means there is IRB approval for not obtaining consent or for not including some of the elements of consent in the consent process.  **NOTE**: If you plan to obtain identifiable information or identifiable biospecimens without consent, any waiver granted by the IRB does not override a subject’s refusal to provide broad consent (for example, the Northwest Biotrust). | | | | | | | | | | |
| **WAIVER OF DOCUMENTATION OF CONSENT** | | | | | | | | | | | | | | | | | | | | | | | means that there is IRB approval for not obtaining written documentation of consent. | | | | | | | | | | |
| **8.1 Groups** Identify the groups to which your answers in this section apply. | | | | | | | | | | | | | | | | | | | | | | | | | | | | | | | | | |
|  | | **x** | | | | | | | Adult subjects | | | | | | | | | | | | | | | | | | | | | | | | |
|  | |  | | | | | | | Parents who are providing permission for their children to participate in research | | | | | | | | | | | | | | | | | | | | | | | | |
|  | | | | | | | | | 🡪 *If you selected* ***PARENTS****, the word “consent” below should also be interpreted as applying to parental permission and “subjects” should also be interpreted as applying to the parents.* | | | | | | | | | | | | | | | | | | | | | | | | |
| **8.2** **The consent process**. This series of questions is about whether you will obtain consent for all procedures except recruiting and screening and, if yes, how. | | | | | | | | | | | | | | | | | | | | | | | | | | | | | | | | | |
| *The issue of consent for recruiting and screening activities is addressed in* [*question 4.6*](#q4point6)*. You do not need to repeat your answer to question 4.6.* | | | | | | | | | | | | | | | | | | | | | | | | | | | | | | | | | |
|  | **a**. Are there any procedures for which you will not obtain consent? | | | | | | | | | | | | | | | | | | | | | | | | | | | | | | | | |
|  | **x** | | | | | | | **No** | | | |  | | | | | | | | | | | | | | | | | | | | | |
|  |  | | | | | | | **Yes** | | | | 🡪 If yes, use the table below to identify the procedures for which you will not obtain consent. “All” is an acceptable answer for some studies. | | | | | | | | | | | | | | | | | | | | | |
|  |  | | | | | | | | | | |  |  |  |  |  |  |  |  |  |  |  |  |  |  |  |  |  |  |  |  |  |  |
| *Be sure to consider all research procedures and plans, including future contact, and sharing/banking of data and specimens for future work.* | | | | | | | | | | | | | | | | | | | | | | | | | | | | | | | | | |
| **Group^1^** | | | | | | | | | | | | **Describe the procedures or data/specimen collection (if any) for which there will be NO consent process** | | | | | | | | | | | | | | **Reason why you will not obtain consent** | **Will you provide subjects with info about the research after they finish?** | | | | | | |
|  | | | | | | | | | | | |  | | | | | | | | | | | | | |  | **YES** | | | **NO** | | | |
|  | | | | | | | | | | | |  | | | | | | | | | | | | | |  |  |  |  | |  |  |  |
|  |  |  |  |  |  |  |  |  |  |  |  |  |  |  |  |  |  |  |  |  |  |  |  |  |  |  |  |  |  | |  |  |  |
|  |  |  |  |  |  |  |  |  |  |  |  |  |  |  |  |  |  |  |  |  |  |  |  |  |  |  |  |  |  | |  |  |  |
|  | | | | | | | | | | | |  | | | | | | | | | | | | | |  |  |  |  | |  |  |  |
|  |  |  |  |  |  |  |  |  |  |  |  |  |  |  |  |  |  |  |  |  |  |  |  |  |  |  |  |  |  | |  |  |  |
|  |  |  |  |  |  |  |  |  |  |  |  |  |  |  |  |  |  |  |  |  |  |  |  |  |  |  |  |  |  | |  |  |  |
|  | | | | | | | | | | | |  | | | | | | | | | | | | | |  |  |  |  | |  |  |  |
|  |  |  |  |  |  |  |  |  |  |  |  |  |  |  |  |  |  |  |  |  |  |  |  |  |  |  |  |  |  | |  |  |  |
|  |  |  |  |  |  |  |  |  |  |  |  |  |  |  |  |  |  |  |  |  |  |  |  |  |  |  |  |  |  | |  |  |  |
|  | | | | | | | | | | | |  | | | | | | | | | | | | | |  |  |  |  | |  |  |  |
|  |  |  |  |  |  |  |  |  |  |  |  |  |  |  |  |  |  |  |  |  |  |  |  |  |  |  |  |  |  | |  |  |  |
|  |  |  |  |  |  |  |  |  |  |  |  |  |  |  |  |  |  |  |  |  |  |  |  |  |  |  |  |  |  | |  |  |  |
|  | | | | | | | | | | | |  | | | | | | | | | | | | | |  |  |  |  | |  |  |  |
|  |  |  |  |  |  |  |  |  |  |  |  |  |  |  |  |  |  |  |  |  |  |  |  |  |  |  |  |  |  | |  |  |  |
|  |  |  |  |  |  |  |  |  |  |  |  |  |  |  |  |  |  |  |  |  |  |  |  |  |  |  |  |  |  | |  |  |  |
| *Table footnotes*   1. *If your answer is the same for all groups you can collapse your answer across the groups and/or procedures.* | | | | | | | | | | | | | | | | | | | | | | | | | | | | | | | | | |
| **b.** Describe the consent process**,** if you will obtain consent for any or all procedures, for any or all groups. Address groups and procedures separately if the consent processes are different. | | | | | | | | | | | | | | | | | | | | | | | | | | | | | | | | | |
| *Be sure to include:*   - *The location/setting where consent will be obtained* - *Who will obtain consent (refer to positions, roles, or titles, not names).* - *Whether/how you will provide an opportunity for questions* - *How you will provide an adequate opportunity for the subjects to consider all options* | | | | | | | | | | | | | | | | | | | | | | | | | | | | | | | | | |
|  | | | | | The PI or another member of the research team will meet with potential participants that are eligible after initial screening either at their home or at a convenient location (such as a community library). There, she/he will describe the study and review the consent form with the participant, answering any questions, and emphasizing that their participation is voluntary. Should the participant wish to delay signing the consent form, they may keep the form and reschedule the baseline visit.  Once signed, the participant will be provided a copy of the consent form. | | | | | | | | | | | | | | | | | | | | | | | | | | | | |
| **c.** Comprehension**.** Describe how you will ensure or test the subjects’ understanding of the information during the consent process. | | | | | | | | | | | | | | | | | | | | | | | | | | | | | | | | | |
|  | | | | | Ask if the subject has any questions. Ask the subject to review the basic study procedure, providing prompts if needed (baseline visits, study duration, follow-up visits). Intervention involves weekly online activities. | | | | | | | | | | | | | | | | | | | | | | | | | | | | |
| **d.** Influence**.** Does your research involve any subject groups that might find it difficult to say “no” to your research because of the setting or their relationship with you, even if you don’t pressure them to participate? | | | | | | | | | | | | | | | | | | | | | | | | | | | | | | | | | |
| *Examples: Student participants being recruited into their teacher’s research; patients being recruited into their healthcare provider’s research, study team members who are participants; outpatients recruited from an outpatient surgery waiting room just prior to their surgery.* | | | | | | | | | | | | | | | | | | | | | | | | | | | | | | | | | |
|  | **x** | | | | | | | **No** | | | |  | | | | | | | | | | | | | | | | | | | | | |
|  |  | | | | | | | **Yes** | | | | 🡪 If yes, describe what you will do, for each of these subject groups, to reduce any effect of the setting or relationship on their decision. | | | | | | | | | | | | | | | | | | | | | |
|  |  | | | | | | | | | | |  |  |  |  |  |  |  |  |  |  |  |  |  |  |  |  |  |  |  |  |  |  |
|  |  | | | | | | | | | | | *Examples: a study coordinator will obtain consent instead of the subjects’ physician; the researcher will not know which subjects agreed to participate; subjects will have two days to decide after hearing about the study.* | | | | | | | | | | | | | | | | | | | | | |
|  | | | | | | | | | | | | | |  | | | | | | | | | | | | | | | | | | | |
| **e.** Ongoing process. For research that involves multiple or continued interaction with subjects over time, describe the opportunities (if any) you will give subjects to ask questions or to change their minds about participating. | | | | | | | | | | | | | | | | | | | | | | | | | | | | | | | | | |
|  | | | | Prior to any subject interaction, study team will ask participants if they have any questions or concerns about participating. They will be reminded that they may stop at any time. During online participation, written prompts remind that they may stop at any time. | | | | | | | | | | | | | | | | | | | | | | | | | | | | | |
| **8.3 Written documentation of consent.** Which of the statements below describe whether you will obtain documentation of consent? NOTE: This question does not apply to screening and recruiting procedures which have already been addressed in [question 4.6](#q4point6). | | | | | | | | | | | | | | | | | | | | | | | | | | | | | | | | | |
| *Documentation of consent that is obtained electronically is not considered written consent unless it is obtained by a method that allows verification of the individual’s signature. In other words, saying “yes” by email is rarely considered to be written documentation of consent* | | | | | | | | | | | | | | | | | | | | | | | | | | | | | | | | | |
| **a.** Are you obtaining written documentation of consent for: | | | | | | | | | | | | | | | | | | | | | | | | | | | | | | | | | |
|  | |  | | | | | | None of your research procedures | | | | | | | | | | | | | | | | | 🡪 Use the table below to provide your justification then go to question **8.4.** | | | | | | | | |
|  | |  | | | | | |  |  |  |  |  |  |  |  |  |  |  |  |  |  |  |  |  |  |  |  |  |  |  |  |  |  |
|  | |  | | | | | |  |  |  |  |  |  |  |  |  |  |  |  |  |  |  |  |  |  |  |  |  |  |  |  |  |  |
|  | |  | | | | | | All of your research procedures | | | | | | | | | | | | | | | | | 🡪 Do not complete the table; go to [question **8.4.**](#q8point4) | | | | | | | | |
|  | | **x** | | | | | |  |  |  |  |  |  |  |  |  |  |  |  |  |  |  |  |  |  |  |  |  |  |  |  |  |  |
|  | |  | | | | | |  |  |  |  |  |  |  |  |  |  |  |  |  |  |  |  |  |  |  |  |  |  |  |  |  |  |
|  | |  | | | | | | Some of your research procedures | | | | | | | | | | | | | | | | | 🡪 Use the table below to identify the procedures for which you will not obtain written documentation of consent from your adult subjects. | | | | | | | | |
|  | |  | | | | | |  |  |  |  |  |  |  |  |  |  |  |  |  |  |  |  |  |  |  |  |  |  |  |  |  |  |
|  | |  | | | | | |  |  |  |  |  |  |  |  |  |  |  |  |  |  |  |  |  |  |  |  |  |  |  |  |  |  |
| **Adult subject group^1^** | | | | | | | | | | | | **Describe the procedures or data/specimen collection (if any) for which there will be NO documentation of consent** | | | | | | | | | | | | | | | **Will you provide them with a written statement describing the research (optional)?** | | | | | | |
|  | | | | | | | | | | | |  | | | | | | | | | | | | | | | **YES** | | | **NO** | | | |
|  | | | | | | | | | | | |  | | | | | | | | | | | | | | |  |  |  | |  |  |  |
|  |  |  |  |  |  |  |  |  |  |  |  |  |  |  |  |  |  |  |  |  |  |  |  |  |  |  |  |  |  | |  |  |  |
|  |  |  |  |  |  |  |  |  |  |  |  |  |  |  |  |  |  |  |  |  |  |  |  |  |  |  |  |  |  | |  |  |  |
|  | | | | | | | | | | | |  | | | | | | | | | | | | | | |  |  |  | |  |  |  |
|  |  |  |  |  |  |  |  |  |  |  |  |  |  |  |  |  |  |  |  |  |  |  |  |  |  |  |  |  |  | |  |  |  |
|  |  |  |  |  |  |  |  |  |  |  |  |  |  |  |  |  |  |  |  |  |  |  |  |  |  |  |  |  |  | |  |  |  |
|  | | | | | | | | | | | |  | | | | | | | | | | | | | | |  |  |  | |  |  |  |
|  |  |  |  |  |  |  |  |  |  |  |  |  |  |  |  |  |  |  |  |  |  |  |  |  |  |  |  |  |  | |  |  |  |
|  |  |  |  |  |  |  |  |  |  |  |  |  |  |  |  |  |  |  |  |  |  |  |  |  |  |  |  |  |  | |  |  |  |
|  | | | | | | | | | | | |  | | | | | | | | | | | | | | |  |  |  | |  |  |  |
|  |  |  |  |  |  |  |  |  |  |  |  |  |  |  |  |  |  |  |  |  |  |  |  |  |  |  |  |  |  | |  |  |  |
|  |  |  |  |  |  |  |  |  |  |  |  |  |  |  |  |  |  |  |  |  |  |  |  |  |  |  |  |  |  | |  |  |  |
|  | | | | | | | | | | | |  | | | | | | | | | | | | | | |  |  |  | |  |  |  |
|  |  |  |  |  |  |  |  |  |  |  |  |  |  |  |  |  |  |  |  |  |  |  |  |  |  |  |  |  |  | |  |  |  |
|  |  |  |  |  |  |  |  |  |  |  |  |  |  |  |  |  |  |  |  |  |  |  |  |  |  |  |  |  |  | |  |  |  |
| *Table footnotes*   1. *If your answer is the same for all adult groups or all procedures, you can collapse your answer across the groups and/or procedures.* | | | | | | | | | | | | | | | | | | | | | | | | | | | | | | | | | |
| **8.4 Non-English-speaking or -reading adult subjects**. Will you enroll adult subjects who do not speak English or who lack fluency or literacy in English? | | | | | | | | | | | | | | | | | | | | | | | | | | | | | | | | | |
|  | **x** | | | | | | **No** | | |  | | | | | | | | | | | | | | | | | | | | | | | |
|  |  | | | | | | **Yes** | | | 🡪 If yes, describe the process you will use to ensure that the oral and written information provided to them during the consent process and throughout the study will be in a language readily understandable to them and (for written materials such as consent forms or questionnaires) at an appropriate reading/comprehension level. | | | | | | | | | | | | | | | | | | | | | | | |
|  |  | | | | | | | | |  |  |  |  |  |  |  |  |  |  |  |  |  |  |  |  |  |  |  |  |  |  |  |  |
|  | | | | | | | | | | | | |  | | | | | | | | | | | | | | | | | | | | |
|  | | | | | | | | | | | | | **a.** Interpretation. Describe how you will provide interpretation and when. Also, describe the qualifications of the interpreter(s) – for example, background, experience, language proficiency in English and in the other language, certification, other credentials, familiarity with the research-related vocabulary in English and the target language. | | | | | | | | | | | | | | | | | | | | |
|  | | | | | | | | | | | | | | | |  | | | | | | | | | | | | | | | | | |
|  | | | | | | | | | | | | | **b.** Translations. Describe how you will obtain translations of all study materials (not just consent forms) and how you will ensure that the translations meet the UW IRB’s requirement that translated documents will be linguistically accurate, at an appropriate reading level for the participant population, and culturally sensitive for the locale in which they will be used. | | | | | | | | | | | | | | | | | | | | |
|  | | | | | | | | | | | | | | | |  | | | | | | | | | | | | | | | | | |
| **8.5** **Barriers to written documentation of consent**. There are many possible barriers to obtaining written documentation of consent. Consider, for example, individuals who are functionally illiterate; do not read English well; or have sensory or motor impairments that may impede the ability to read and sign a consent form. | | | | | | | | | | | | | | | | | | | | | | | | | | | | | | | | | |
| **a.** Describe your plans (if any) for obtaining written documentation of consent from potential subjects who may have difficulty with the standard documentation process (that is, reading and signing a consent form). Skip this question if you are not obtaining written documentation of consent for any part of your research. | | | | | | | | | | | | | | | | | | | | | | | | | | | | | | | | | |
| *Examples of solutions: Translated consent forms; use of the Short Form consent process; reading the form to the person before they sign it; excluding individuals who cannot read and understand the consent form.* | | | | | | | | | | | | | | | | | | | | | | | | | | | | | | | | | |
|  | | | | | | Eligibility requirements for the study include written/spoken English. Those who are functionally illiterate will not be eligible. In the event that sensory or motor impairments are identified at the time of enrollment, such individuals likely would not meet the eligibility requirements of the study and would not be enrolled. | | | | | | | | | | | | | | | | | | | | | | | | | | | |
| **8.6 Deception**. Will you deliberately withhold information or provide false information to any of the subjects? *Note: “Blinding” subjects to their study group/condition/arm is not considered to be deception.* | | | | | | | | | | | | | | | | | | | | | | | | | | | | | | | | | |
|  | **x** | | **No** | | | | | | | |  | | | | | | | | | | | | | | | | | | | | | | |
|  |  | | **Yes** | | | | | | | | 🡪 If yes, describe what information and why. | | | | | | | | | | | | | | | | | | | | | | |
|  |  | | | | | | | | | | *Example: you may wish to deceive subjects about the purpose of the study.* | | | | | | | | | | | | | | | | | | | | | | |
|  | | | | | | | | | | | | |  | | | | | | | | | | | | | | | | | | | | |
|  | | | | | | | | | | | | | **a.** Will you debrief the subjects later? (Note: this is not required.) | | | | | | | | | | | | | | | | | | | | |
|  | | | | | | | | | | | | | | |  | | | | **No** | |  | | | | | | | | | | | | |
|  | | | | | | | | | | | | | | |  | | | | **Yes** | | | 🡪 If yes, describe how you will debrief the subjects. Upload any debriefing materials, including talking points or a script, to the **Consent Form and Recruitment Materials** SmartForm of ***Zipline***. | | | | | | | | | | | |
|  | | | | | | | | | | | | | | |  | | | | | | |  |  |  |  |  |  |  |  |  |  |  |  |
|  | | | | | | | | | | | | | | | | | | | | | | | |  | | | | | | | | | |
| **8.7 Cognitively impaired adults, and other adults unable to consent.** Do you plan to include such individuals in your research? | | | | | | | | | | | | | | | | | | | | | | | | | | | | | | | | | |
| *Examples: individuals with Traumatic Brain Injury (TBI) or dementia; individuals who are unconscious, or who are significantly intoxicated.* | | | | | | | | | | | | | | | | | | | | | | | | | | | | | | | | | |
|  | **x** | | | | | | | **No** | | | | 🡪 If no, go to [question](#q8point7) **8.8**. | | | | | | | | | | | | | | | | | | | | | |
|  |  | | | | | | | **Yes** | | | | 🡪 If yes, answer the following questions. | | | | | | | | | | | | | | | | | | | | | |
|  | | | | | | | | | | | | | | **a.** Rationale. Provide your rationale for including this population in your research. | | | | | | | | | | | | | | | | | | | |
|  | | | | | | | | | | | | | | | | | |  | | | | | | | | | | | | | | | |
|  | | | | | | | | | | | | | | **b.** Capacity for consent / decision making capacity. Describe the process you will use to determine whether a cognitively impaired individual is capable of consent decision making with respect to your research protocol and setting. | | | | | | | | | | | | | | | | | | | |
|  | | | | | | | | | | | | | |  | | | |  | | | | | | | | | | | | | | | |
|  | | | | | | | | | | | | | | | | | **b.1.** If you will have repeated interactions with the impaired subjects over a time period when cognitive capacity could increase or diminish, also describe how (if at all) you will reassess decision-making capacity and obtain consent during that time. | | | | | | | | | | | | | | | | |
|  | | | | | | | | | | | | | | | | | | | |  | | | | | | | | | | | | | |
|  | | | | | | | | | | | | | | **c.** Permission (surrogate consent). If you will include adults who cannot consent for themselves, describe your process for obtaining permission (“surrogate consent”) from a legally authorized representative (LAR). | | | | | | | | | | | | | | | | | | | |
|  | | | | | | | | | | | | | | *For research conducted in Washington State, see the* [***SOP: Legally Authorized Representative***](https://www.washington.edu/research/policies/sop-legally-authorized-representative-2/) *to learn which individuals meet the state definition of “legally authorized representative”.* | | | | | | | | | | | | | | | | | | | |
|  | | | | | | | | | | | | | |  | | | |  | | | | | | | | | | | | | | | |
|  | | | | | | | | | | | | | | **d**. Assent. Describe whether assent will be required of all, some, or none of the subjects. If some, indicate which subjects will be required to assent and which will not (and why not). Describe any process you will use to obtain and document assent from the subjects. | | | | | | | | | | | | | | | | | | | |
|  | | | | | | | | | | | | | |  | | | |  | | | | | | | | | | | | | | | |
|  | | | | | | | | | | | | | | **e.** Dissent or resistance. Describe how you will identify the subject’s objection or resistance to participation (including non-verbal) during the research, and what you will do in response. | | | | | | | | | | | | | | | | | | | |
|  | | | | | | | | | | | | | |  | | | |  | | | | | | | | | | | | | | | |
| **8.8 Consent-related materials**. Upload to the **Consent Forms and Recruitment Materials** SmartForm of ***Zipline*** all consent scripts/talking points, consent forms, debriefing statements, Information Statements, Short Form consent forms, parental permission forms, and any other consent-related materials you will use. | | | | | | | | | | | | | | | | | | | | | | | | | | | | | | | | | |
| - *Translations must be included. However, you are strongly encouraged to wait to provide them until you know that the IRB will approve the English versions.* - *Combination forms: It may be appropriate to combine parental permission with consent, if parents are subjects as well as providing permission for the participation of their children. Similarly, a consent form may be appropriately considered an assent form for older children.* - *For materials that cannot be uploaded: upload screenshots or written descriptions that are sufficient to enable the IRB to understand the types of data that will be collected and the nature of the experience for the participant. You may also provide URLs (website addresses) or written descriptions below. Examples of materials that usually cannot be uploaded: mobile apps; computer-administered test; licensed and restricted standardized tests.* | | | | | | | | | | | | | | | | | | | | | | | | | | | | | | | | | |

| **9 PRIVACY AND CONFIDENTIALITY** | | | | | | | | | |
| --- | --- | --- | --- | --- | --- | --- | --- | --- | --- |
| **9.1 Privacy protections.** Describe the steps you will take, if any, to address possible privacy concerns of subjects and potential subjects. | | | | | | | | | |
| *Privacy refers to the sense of being in control of access that others have to ourselves. This can be an issue with respect to recruiting, consenting, sensitivity of the data being collected, and the method of data collection.*  *Examples:*   - *Many subjects will feel a violation of privacy if they receive a letter asking them to participate in a study because they have ____ medical condition, when their name, contact information, and medical condition were drawn from medical records without their consent. Example: the IRB expects that “cold call” recruitment letters will inform the subject about how their information was obtained.* - *Recruiting subjects immediately prior to a sensitive or invasive procedures (e.g., in an outpatient surgery waiting room) will feel like an invasion of privacy to some individuals.* - *Asking subjects about sensitive topics (e.g. details about sexual behavior) may feel like an invasion of privacy to some individuals.* | | | | | | | | | |
|  | This research will gather several types of sensitive information, around which special care must be taken. We will take measures to ensure that this information is handled appropriately, safely, and only for the scope of this research. There are additional privacy and confidentiality concerns with reporting health-related information in the study.  **Loss of Privacy**  Participants will be informed that their information will be kept confidential. Participant confidentiality will be protected by using a study identification number on all records. Data collection forms used during the study will be surveys and identified only by assigned code number. Participants will complete sleep diaries through the web-based electronic data capture interface, which is password-protected and encrypted (no direct identifiers will be present among the electronic data). Research paper forms will be kept in a locked file cabinet in the PI’s office separate from the consent forms and code list, available only to authorized research colleagues assisting with data analysis. No participants will be identified in any report or publication about this study. Data collected via surveys will use REDCap, which is password protected and encrypted, with no direct identifies present among electronic data.  The protection of human subjects in the proposed study is ensured through control of access to the data, maintenance of locked files for data and consent forms, and careful training and supervision of all research personnel. Subject identifying information will be accessible only to study personnel with appropriate access privileges and will be kept in a locked office in a locked file, separate from other study materials. Electronic data will be stored on a secure server that requires password access form a computer with a registered domain to gain entry.  Risk of unauthorized disclosure of an individual’s data to others outside of the study personnel is possible. To protect information, we will keep subject names or any number that could identify them separate from study data. Subjects will be assigned codes and individual access to OPTIMISM web materials. | | | | | | | | |
| **9.2 Identification of individuals in publications and presentations**. Do you plan to use potentially identifiable information about subjects in publications and presentations, or is it possible that individual identities could be inferred from what you plan to publish or present? | | | | | | | | | |
|  | **x** | | **No** |  | | | | | |
|  |  | | **Yes** | 🡪 If yes, will you obtain subject consent for this use? | | | | | |
|  | | | | |  | | **Yes** |  | |
|  | | | | |  | | **No** | 🡪 If no, describe the steps you will take to protect subjects (or small groups of subjects) from being identifiable. | |
|  | | | | |  | |  |  |  |
|  | | | | | | | | |  |
| **9.3 State mandatory reporting.** Each state has reporting laws that require some types of individuals to report some kinds of abuse, and medical conditions that are under public health surveillance. These include:   - Child abuse - Abuse, abandonment, neglect, or financial exploitation of a vulnerable adult - Sexual assault - Serious physical assault - Medical conditions subject to mandatory reporting (notification) for public health surveillance   Are you or a member of your research team likely to learn of any of the above events or circumstances while conducting your research **AND** feel obligated to report it to state authorities? | | | | | | | | | |
|  | **x** | **No** | |  | | | | | |
|  |  | **Yes** | | 🡪 If yes, the UW IRB expects you to inform subjects of this possibility in the consent form or during the consent process, unless you provide a rationale for not doing so: | | | | | |
|  |  | | |  |  |  |  |  |  |
|  |  | | |  | |  | | | |
| **9.4 Retention of identifiers and data.** Check the box below to indicate your assurance that you will not destroy any identifiers (or links between identifiers and data/specimens) and data that are part of your research records until after the end of the applicable records retention requirements (e.g. Washington State; funding agency or sponsor; Food and Drug Administration) for your research. If you think it is important for your specific study to say something about destruction of identifiers (or links to identifiers) in your consent form, state something like “the link between your identifier and the research data will be destroyed after the records retention period required by state and/or federal law.” | | | | | | | | | |
| *This question can be left blank for conversion applications (existing paper applications that are being “converted” into a Zipline application.)*  *See the “Research Data” sections of the following website for UW Records management for the Washington State research rectords retention schedules that apply in general to the UW (not involving UW Medicine data):* [***http://f2.washington.edu/fm/recmgt/gs/research?title=R***](http://f2.washington.edu/fm/recmgt/gs/research?title=R)  *See the “Research Data and Records” information in Section 8 of this document for the retention schedules for UW Medicine Records:* [***http://www.uwmedicine.org/about/Documents/UWM-Records-Retention-Schedule-v1.6.pdf***](http://www.uwmedicine.org/about/Documents/UWM-Records-Retention-Schedule-v1.6.pdf) | | | | | | | | | |
|  | **x** | | **Confirm** | | | | | | |
| **9.5 Certificates of Confidentiality**. Are you planning to obtain a federal Certificate of Confidentiality for your research data? *NOTE: Answer “No” if your study is NIH funded, because all NIH-funded studies automatically have a Certificate.* | | | | | | | | | |
|  | **x** | **No** | |  | | | | | |
|  |  | **Yes** | |  | | | | | |
| **9.6 Data and specimen security protections**. Identify your data classifications and the security protections you will provide, referring to the [***ZIPLINE* GUIDANCE: Data and Security Protections**](https://www.washington.edu/research/policies/zipline-guidance-data-security-protections-2/) for the minimum requirements for each data classification level. ***You cannot answer this question without reading this document. Data security protections should not conflict with records retention requirements.*** | | | | | | | | | |
|  | **a.** Which level of protections will you apply to your data and specimens? If you will use more than one level, describe which level will apply to which data and which specimens. | | | | | | | | |
|  |  | Level 3 protections will be used. | | | | | | | |
|  | **b.** Use this space to provide additional information, details, or to describe protections that do not fit into one of the levels. If there are any protections within the level listed in 9.6.a which you will *not* follow, list those here. | | | | | | | | |
|  |  | A study participant file will be assembled that contains the consent form, contact information, and screening forms confirming eligibility criteria for each participant in this study. The PI will need identifying information in order to document informed consent and eligibility and to contact participants for reminders and appointments. Identifying information will be accessible only to study personnel and will be kept in a locked office in a locked file in the PI’s office, separate from data collected during the course of the study and separate from the link between study data and participant identity.  Study participants will be assigned a number in the order that they enroll in the study. The link between participant identity and study code number (the master list) will be kept in a locked office and a locked file, separate from data collected during the course of the study.  Data collection forms used during the study will be electronic using the REDCap platform, using only the study code numbers. The REDCap system is password-protected and encrypted.  Only members of the research team will have access to links and identifiers. De-identified study data is kept securely on a password-protected, encrypted electronic data management system. The study sponsor (NINR) is permitted to review data and identifying information on subjects. No other agencies or researchers will be permitted to access this information. | | | | | | | |

| **10 RISK / BENEFIT ASSESSMENT** | | | | | | | | |
| --- | --- | --- | --- | --- | --- | --- | --- | --- |
| **10.1 Anticipated risks**. Describe the reasonably foreseeable risks of harm, discomforts, and hazards to the subjects and others of the research procedures. For each harm, discomfort, or hazard:   - Describe the magnitude, probability, duration, and/or reversibility of the harm, discomfort, or hazard, AND - Describe how you will manage or reduce the risks. Do not describe data security protections here, these are already described in Question 9.6. | | | | | | | | |
| - *Consider possible physical, psychological, social, legal, and economic harms, including possible negative effects on financial standing, employability, insurability, educational advancement or reputation. For example, a breach of confidentiality might have these effects.* - *Examples of “others”: embryo, fetus, or nursing child; family members; a specific group.* - *Do not include the risks of non-research procedures that are already being performed.* - *If the study design specifies that subjects will be assigned to a specific condition or intervention, then the condition or intervention is a research procedure - even if it is a standard of care.* - *Examples of mitigation strategies: inclusion/exclusion criteria; applying appropriate data security measures to prevent unauthorized access to individually identifiable data; coding data; taking blood samples to monitor something that indicates drug toxicity.* - *As with all questions on this application, you may refer to uploaded documents.* | | | | | | | | |
|  | This is a minimal risk study and the risks of this study are related to:   - Potential loss of confidentiality and invasion of privacy: This research will gather several types of sensitive information, around which special care must be taken. We will take measures to ensure that this information is handled appropriately, safely, and only for the scope of this research. There are additional privacy and confidentiality concerns with reporting health-related information in the study. - Mild discomfort related to Actiwatch: The Actiwatch may irritate the skin and participants will have the option to wear it over a long sleeve shirt. Skin reactions of this kind are unusual, and the PI will be available to troubleshoot with participants as needed. - Surveys: Some questions in the questionnaires explore health history of medical and/or psychological illness, which may be upsetting to participants. Participants may choose not to answer any questions causing discomfort, and may stop questionnaires at any time. Other questions could result in discovery of symptoms of clinical depression or suicidality. Data will be reviewed by the research staff for any elevated depression or anxiety screenings and the participant will be contacted by the PI. The PI will offer community resources (help and referral lines, therapists and psychiatric providers, and support groups) and encourage the participant to contact her primary care provider or prenatal care provider for appropriate management and follow up.   All participants will be reminded that taking part in research is voluntary. No one will be mad at them if they say no or discontinue their participation. Participants can change their minds at any time. Participants will be assured, both verbally and in writing that they may withdraw from the study at any time. Prior to the conduct of any study-related procedures, the informed consent statement will be reviewed. Participant refusal to engage in study procedures will be honored and participation discontinued. A copy of the informed consent forms will be given to the participant during the T1 baseline visit, and the original will be placed in a locked file cabinet in the PI’s office.  **Actigraphy**  Participants will be instructed in use of the Actigraph including removal during bathing, swimming, and showering, and taught how to assess correct tightness of the Velcro monitor band. Participants will document periods when the Actigraph monitor is removed and replaced in the electronic diary. Participants will wear the Actigraph watch for 8 days at a time, at two different time points. Actigraphs will be collected via prepaid mailed envelope, at a drop-off locations (the UW School of Nursing), or by research staff in-person. Participants will be told they may wear the Actigraph over a long sleeve shirt to prevent irritation.  **Survey**  For the surveys, the research staff will ask participants to complete the questionnaires online and inform them that there are no “correct” or “right” answers. Participants will also be told that they do not have to answer any question that they do not want to.  **Social Risks**  To minimize embarrassment or anxiety about Actiwatch, participants may wear a long sleeve shirt over the watch.  **Usability Testing**  This optional procedure will include digital video and audio recording of participants interacting with the online intervention. Participants may feel uncomfortable knowing that they are being observed and recorded. This procedure will proceed in a room with a closed door, and the recording will be paused if a non-consented person (such as a family member) enters the room or otherwise may be recorded. Recordings will be edited to delete any inadvertent recording of non-consented persons, Although unlikely, there is the risk of loss of confidentiality or privacy from unauthorized access to the recordings. Recordings will be stored on password protected secure servers accessible only to authorized research staff. | | | | | | | |
| **10.2 Reproductive risks**. Are there any risks of the study procedures to men and women (who are subjects, or partner of subjects) related to pregnancy, fertility, lactation or effects on a fetus or neonate? | | | | | | | | |
| *Examples: direct teratogenic effects; possible germline effects; effects on fertility; effects on a woman’s ability to continue a pregnancy; effects on future pregnancies.* | | | | | | | | |
|  | | **x** | **No** | | 🡪 If no go to [question 10.3](#q10point3) | | | |
|  | |  | **Yes** | | 🡪 If yes, answer the following questions: | | | |
|  | | | | | **a. Risks**. Describe the magnitude, probability, duration and/or reversibility of the risks. | | | |
|  | |  | | |  |  | | |
|  | | | | | **b. Steps to minimize risk**. Describe the specific steps you will take to minimize the magnitude, probability, or duration of these risks. | | | |
| *Examples: inform the subjects about the risks and how to minimize them; require a pregnancy test before and during the study; require subjects to use contraception; advise subjects about banking of sperm and ova.*  *If you will require the use of contraception: describe the allowable methods and the time period when contraception must be used.* | | | | | | | | |
|  | |  | | |  |  | |  |
|  | | | | | **c. Pregnancy**. Describe what you will do if a subject (or a subject’s partner) becomes pregnant | | | |
| *For example; will you require the subject to immediately notify you, so that you can discontinue or modify the study procedures, discuss the risks, and/or provide referrals or counseling?* | | | | | | | | |
|  | |  | | |  |  | |  |
| **10.3 Unforeseeable risks**. Are there any research procedures that may have risks that are currently unforeseeable? | | | | | | | | |
| *Example: using a drug that hasn’t been used before in this subject population.* | | | | | | | | |
|  | | **x** | **No** | |  | | | |
|  | |  | **Yes** | | 🡪 If yes, identify the procedures. | | | |
|  | |  | | |  |  | |  |
| **10.4** **Subjects who will be under regional or general anesthesiology.** Will any research procedures occur while subjects-patients are under general or regional anesthesia, or during the 3 hours preceding general or regional anesthesia (supplied for non-research reasons)? | | | | | | | | |
|  | | **x** | **No** | |  | | |  |
|  | |  | **Yes** | | 🡪 If yes, check all the boxes that apply. | | |  |
|  | |  |  | | |  | Administration of any drug for research purposes |  |
|  | |  |  | | |  |  |  |
|  | |  |  | | |  |  |  |
|  | | | | | |  | Inserting an intra-venous (central or peripheral) or intra-arterial line for research purposes |  |
|  | | | | | |  |  |  |
|  | | | | | |  |  |  |
|  | | | | | |  | Obtaining samples of blood, urine, bone marrow or cerebrospinal fluid for research purposes |  |
|  | | | | | |  |  |  |
|  | | | | | |  |  |  |
|  | | | | | |  | Obtaining a research sample from tissue or organs that would not otherwise be removed during surgery |  |
|  | | | | | |  |  |  |
|  | | | | | |  |  |  |
|  | | | | | |  | Administration of a radio-isotope for research purposes** |  |
|  | | | | | |  |  |  |
|  | | | | | |  |  |  |
|  | | | | | |  | Implantation of an experimental device |  |
|  | | | | | |  |  |  |
|  | | | | | |  |  |  |
|  | | | | | |  | Other manipulations or procedures performed solely for research purposes (e.g., experimental liver dialysis, experimental brain stimulation) |  |
|  | | | | | |  |  |  |
|  | | | | | |  |  |  |
|  | | | | | |  | If you checked any of the boxes:  You must provide the name and institutional affiliation of a physician anesthesiologist who is a member of your research team or who will serve as a safety consultant about the interactions between your research procedures and the general or regional anesthesia of the subject-patients. If your procedures will be performed at a UW Medicine facility or affiliate, the anesthesiologist must be a UW faculty member. |  |
|  | | | | | |  |  |  |
|  | | | | | |  | *** If you checked the box about radio-isotopes: you are responsible for informing in advance all appropriate clinical personnel (e.g., nurses, technicians, anesthesiologists, surgeons) about the administration and use of the radio-isotope, to ensure that any personal safety issues (e.g., pregnancy) can be appropriately addressed. This is a condition of IRB approval.* |  |
| **10.5 Data and Safety Monitoring**. A Data and Safety Monitoring Plan (DSMP) is required for clinical trials (as defined by NIH). If required for your research, upload your DSMP to the **Supporting Documents** SmartForm in ***Zipline***. If it is embedded in another document you are uploading (for example, a Study Protocol, use the text box below to name the document that has the DSMP. | | | | | | | | |
|  | | See uploaded document DSMP OPTIMISM | | | | | | |
| **10.6 Un-blinding.** If this is a double-blinded or single-blinded study in which the participant and/or you do not know the group to which the participant is assigned: describe the circumstances under which un-blinding would be necessary, and to whom the un-blinded information would be provided. | | | | | | | | |
|  | | n/a | | | | | | |
| **10.7 Withdrawal of participants.** If applicable, describe the anticipated circumstances under which participants will be withdrawn from the research without their consent. Also, describe any procedures for orderly withdrawal of a participant, regardless of the reason, including whether it will involve partial withdrawal from procedures and any intervention but continued data collection or long-term follow-up. | | | | | | | | |
|  | | Participants may withdraw from the study at any time. Participants will also be asked to provide rationale behind their decision to withdraw, though they may choose not to provide rationale. The PI will ask if the participants are willing to continue with follow-up assessments (T2), but participants may choose to participate in data collection or not. | | | | | | |
| **10.8 Anticipated direct benefits to participants**. If there are any direct research-related benefits that some or all individual participants are likely to experience from taking part in the research, describe them below: | | | | | | | | |
| *Do not include benefits to society or others, and do not include subject payment (if any). Examples: medical benefits such as laboratory tests (if subjects receive the results); psychological resources made available to participants; training or education that is provided.* | | | | | | | | |
|  | | Benefits to participants include learning about their sleep patterns and strategies that may improve sleep self-management. | | | | | | |
| **10.9 Individual subjects findings.** | | | | | | | | |
|  | | **a**. Is it likely that your research will unintentionally discover a previously unknown condition such as a disease, suicidal intentions, or genetic predisposition? | | | | | | |
|  | | | **x** | **No** | |  | | |
|  | | |  | **Yes** | | 🡪 If yes, explain whether and how you would share the information with the subject. | | |
|  | | |  | | |  |  |  |
|  | | **b**. Do you plan to share the individual results of any of your study procedures or findings with the subjects – such as genetic test results, laboratory tests, etc.? | | | | | | |
|  | | *You should answer YES if your consent form says anything about sharing individual information with subjects.* | | | | | | |
|  | | | **x** | **No** | |  | | |
|  | | |  | **Yes** | | 🡪 If yes, complete and upload the [**SUPPLEMENT: Participant Results Sharing**](https://www.washington.edu/research/forms-and-templates/zipline-supplement-participant-results-sharing/) to the **Supporting Documents** SmartForm of ***Zipline*** | | |
|  | | |  | | |  | | |
| **10.10 Commercial products or patents**. Is it possible that a commercial product or patent could result from this study? | | | | | | | | |
|  | | **x** | **No** | |  | | | |
|  | |  | **Yes** | | 🡪 If yes, describe whether subjects might receive any remuneration/compensation and, if yes, how the amount will be determined. | | | |
|  | | | | |  | | | |
|  | |  | | |  |  | |  |

| **11 ECONOMIC BURDEN TO PARTICIPANTS** | |
| --- | --- |
| **11.1 Financial responsibility for research-related injuries.** Answer this question only if the lead researcher is not a UW student, staff member, or faculty member whose primary paid appointment is at the UW.  Describe who will be financially responsible for research-related injuries experienced by subjects, and any limitations. Describe the process (if any) by which participants may obtain treatment/compensation. | |
|  |  |
| **11.2 Costs to subjects**. Describe any research-related costs for which subjects and/or their health insurance may be responsible (examples might include: CT scan required for research eligibility screening; co-pays; surgical costs when a subject is randomized to a specific procedure; cost of a device; travel and parking expenses that will not be reimbursed). | |
|  | n/a |
| **11.3 Reimbursement for costs.** Describe any costs to subjects that will be reimbursed (such as travel expenses). | |
|  | n/a |

| **12 RESOURCES** | | |
| --- | --- | --- |
| **12.1 Faculty Advisor.** (For researchers who are students, fellows, or post-docs.) Provide the following information about your faculty advisor.   - Advisor’s name - Your relationship with your advisor (for example: graduate advisor; course instructor) - Your plans for communication/consultation with your advisor about progress, problems, and changes. | | |
|  | n/a | |
| **12.2 Study team communication**. Describe how you will ensure that each study team member is adequately trained and informed about the research procedures and requirements (including any changes) as well as their research-related duties and functions. | | |
|  |  | **There is no study team.** |
|  |  |  |
|  | Procedure manuals will be used and checklists for data collection sessions. Any research assistants will be trained by the PI and monitored for adherence to procedures. | |

| **13 OTHER APPROVALS, PERMISSIONS, and REGULATORY ISSUES** | | | | | | | | |
| --- | --- | --- | --- | --- | --- | --- | --- | --- |
| **13.1 Other regulatory approvals**. Identify any other regulatory approvals that are required for this research, by checking applicable boxes | | | | | | | | |
| *Do not attach the approvals unless requested by the IRB.* | | | | | | | | |
|  | **Approval** | | | | | | | **Research for which this is required** |
|  |  | | | Radiation Safety | | | | Procedures involving the use of radioactive materials or an ionizing radiation producing machine radiation, if they are conducted for research rather than clinical purposes. Approvals need to be attached to the Supporting Documents page in ***Zipline***. |
|  |  | | |  |  |  |  |  |
|  |  | | |  |  |  |  |  |
|  |  | | | Institutional Biosafety | | | | Procedures involving the transfer/administration of recombinant DNA, DNA/RNA derived from recombinant DNA, or synthetic DNA. |
|  |  | | |  |  |  |  |  |
|  |  | | |  |  |  |  |  |
|  |  | | | RDRC | | | | Procedures involving a radioactive drug or biological product that is not approved by the FDA for the research purpose and that is being used without an IND, for basic science research (not to determine safety and effectiveness, or for immediate therapeutic or diagnostic purposes). |
|  |  | | |  |  |  |  |  |
|  |  | | |  |  |  |  |  |
|  |  | | | ESCRO | | | | Procedures involving the use of some types of human embryonic stem cells. |
|  |  | | |  |  |  |  |  |
|  |  | | |  |  |  |  |  |
| **13.2** **Approvals and permissions.** Identify any other approvals or permissions that will be obtained. For example: from a school, external site/organization, funding agency, employee union, UW Medicine clinical unit. | | | | | | | | |
| *Do not attach the approvals and permissions unless requested by the IRB.* | | | | | | | | |
|  | | | n/a | | | | | |
| **13.3** **Financial Conflict of Interest.** Does any member of the team have a Financial Conflict of Interest (FCOI) in this research, as defined by [UW policy GIM 10](https://www.washington.edu/research/policies/gim-10-financial-conflict-of-interest-policy/)? | | | | | | | | |
|  | | **x** | | | **No** |  | | |
|  | |  | | | **Yes** | 🡪 If yes, upload the Conflict Management Plan for every team member who has a FCOI with respect to this research, to the **Supporting Documents** page of ***Zipline***. If it is not yet available, use the text box to describe whether the Significant Financial Interest has been disclosed already to the UW Office of Research. | | |
|  | |  | | | |  |  |  |
|  |  | | | | | |  | |
